# The Ras GTPase‐activating‐like protein IQGAP1 bridges Gasdermin D to the ESCRT system to promote IL‐1β release via exosomes

**DOI:** 10.15252/embj.2022110780

**Published:** 2022-11-14

**Authors:** Yun Liao, Xing Chen, William Miller‐Little, Han Wang, Belinda Willard, Katarzyna Bulek, Junjie Zhao, Xiaoxia Li

**Affiliations:** ^1^ Department of Inflammation and Immunity Cleveland Clinic Lerner Research Institute Cleveland OH USA; ^2^ Proteomics and Metabolomics Core Cleveland Clinic Lerner Research Institute Cleveland OH USA

**Keywords:** ESCRT, exosome, GSDMD, inflammasome, IQGAP1, Immunology

## Abstract

IL‐1β can exit the cytosol as an exosomal cargo following inflammasome activation in intestinal epithelial cells (IECs) in a Gasdermin D (GSDMD)‐dependent manner. The mechanistic connection linking inflammasome activation and the biogenesis of exosomes has so far remained largely elusive. Here, we report the Ras GTPase‐activating‐like protein IQGAP1 functions as an adaptor, bridging GSDMD to the endosomal sorting complexes required for transport (ESCRT) machinery to promote the biogenesis of pro‐IL‐1β‐containing exosomes in response to NLPR3 inflammasome activation. We identified IQGAP1 as a GSDMD‐interacting protein through a non‐biased proteomic analysis. Functional investigation indicated the IQGAP1‐GSDMD interaction is required for LPS and ATP‐induced exosome release. Further analysis revealed that IQGAP1 serves as an adaptor which bridges GSDMD and associated IL‐1β complex to Tsg101, a component of the ESCRT complex, and enables the packaging of GSDMD and IL‐1β into exosomes. Importantly, this process is dependent on an LPS‐induced increase in GTP‐bound CDC42, a small GTPase known to activate IQGAP1. Taken together, this study reveals IQGAP1 as a link between inflammasome activation and GSDMD‐dependent, ESCRT‐mediated exosomal release of IL‐1β.

## Graphical abstract

The adaptor protein IQGAP1 links inflammasome activation to cargo selection and exosome release via the interaction with Gasdermin D.

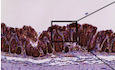

## Introduction

Exosomes are lipid bilayer‐enclosed extracellular vesicles of endosomal origin (Meldolesi, 2018; Mathieu *et al*, 2019; Kalluri & LeBleu, 2020). Initially thought to be a waste disposal system that cells utilize to discharge harmful products, a growing body of evidence indicates exosomes actually carry a wide range of bioactivities, which reflect the spectrum of cargo molecules enclosed in the lipid bilayer (Meldolesi, 2018; Mathieu *et al*, 2019; Kalluri & LeBleu, 2020). Importantly, exosomal cargo often features a selective set of proteins that are specifically enriched in exosomes. Befittingly, molecular mechanisms underlying the selective packaging of specific cargo during exosome biogenesis is a pivotal question in exosome research.

Several early studies have identified the endosomal sorting complexes required for transport (ESCRT) system as a key executor of exosome formation and cargo packaging (Razi & Futter, 2006; Raiborg & Stenmark, 2009; Wollert & Hurley, 2010; Colombo *et al*, 2013; Coulter *et al*, 2018). The classical model of exosome biogenesis proposes that the ESCRT system captures polyubiquitinated cargo proteins as it mediates the invagination of the endosomal membrane, eventually enclosing the polyubiquitinated target protein into exosome precursors, or intraluminal vesicles (ILVs) contained within the late endosome that have matured into multivesicular bodies (MVBs) (Meldolesi, 2018; Mathieu *et al*, 2019; Kalluri & LeBleu, 2020). However, evidence supporting this classical model primarily comes from studies which used integral membrane proteins as model cargos and were mostly concerned with cells at steady state. It is unclear how cytosolic proteins are selectively packaged into exosomes, especially in response to external stimuli.

Exosomal release is one of the secretory routes for interleunkin‐1β (IL‐1β) (Qu *et al*, 2007), which relies on non‐conventional pathways such as pyroptosis for secretion (van Niel *et al*, 2011). The release of IL‐1β requires the activation of inflammasomes which have long been shown to associate with the release of exosome or exosome‐like extracellular vesicles in a variety of cells (Lorey *et al*, 2017; Cypryk *et al*, 2018). These characteristics make IL‐1β a prototypic exosomal cargo that are selectively packaged in a stimulus dependent manner.

We have recently reported that the activation of NLRP3 inflammasome in IECs induces exosomal release of polyubiquitinated pro‐IL‐1β (Bulek *et al*, 2020). This process is dependent on a poorly understood non‐pyroptotic activity of GSDMD. In contrast to GSDMD's role in pyroptosis, where caspases release its lipid‐binding N‐terminal fragment to form pores on the plasma membrane, a full‐length GSDMD, engaged with a chaperone protein Hsp90 and co‐chaperone CDC37, recruits the E3 ligase, NEDD4, to catalyze polyubiquitination of pro‐IL‐1β, while the latter is released via CD63 positive exosomes. In this study, we delineate how this GSDMD‐dependent process is connected to the ESCRT system, the apparatus indispensable for membrane remodeling in classical model of exosome biogenesis.

## Results

### IQGAP1 interacts with GSDMD and mediates the production of GSDMD‐dependent pro‐IL‐1β‐containing sEVs

**Figure EV1:**
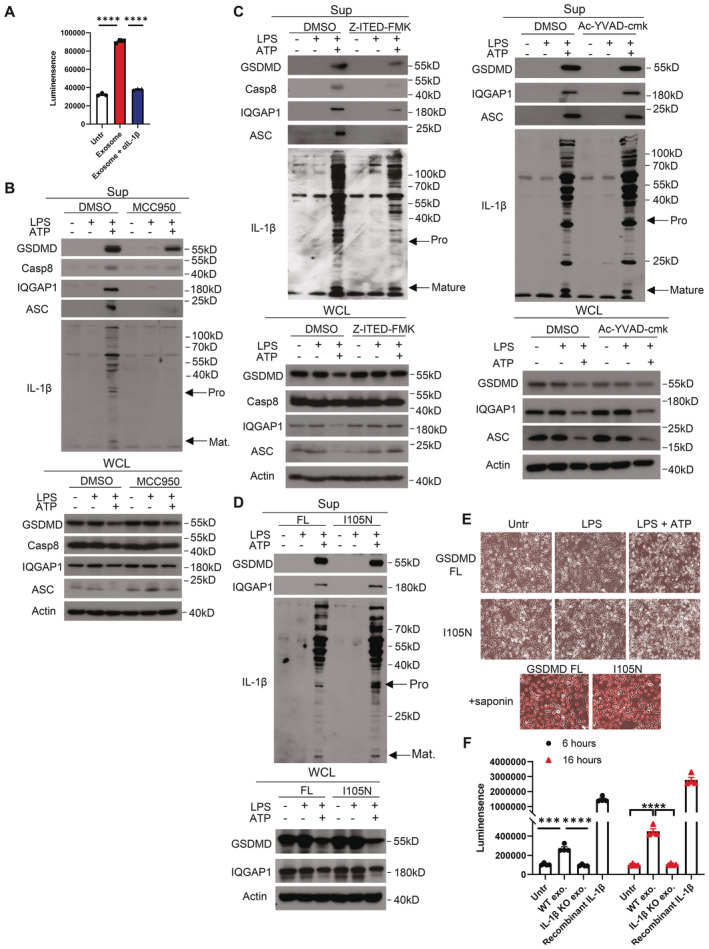
LPS and ATP‐induces nonpyroptotic NLRP3 inflammasome activation Exosomal proteins concentrated from 10^7^ exosomes were prepared from supernatants of YAMCs that were left untreated or treated with LPS (4 h) and ATP (30 min). HEK293 cells stably expressing IL‐1R1 were transfected with an NFκB luciferase reporter construct and stimulated with indicated exosome preparations in the presence or absence of anti‐IL‐1β overnight. Cell lysates were analyzed for luciferase activity. Data were presented as mean ± SEM.Western blot analysis of supernatants and whole‐cell lysates collected from YAMC cells with and without LPS stimulation for 4 h or LPS plus ATP (4 h plus 30 min) in the presence or absence of MCC950 (100 nM).Western blot analysis of supernatants and whole‐cell lysates collected from YAMC cells with and without LPS stimulation for 4 h or LPS plus ATP (4 h plus 30 min) in the presence or absence of Z‐IETD‐FMK (10 μM) or Ac‐YVAD‐cmk (10 μM).Western blot analysis of supernatants and whole‐cell lysates collected from GSDMD KO YAMC cells restored with GSDMD full length (FL) or I105N mutant. Cells were stimulated with or without LPS stimulation for 4 h or LPS plus ATP (4 h plus 30 min) as indicated.GSDMD KO YAMC cells expressing either full length GSDMD or I105N mutant were stimulated with or without LPS for 4 h or LPS plus ATP (4 h plus 30 min) in phenol red‐free DMEM supplemented with 1 μg/ml PI. As controls, the cells were treated with 0.05% saponin for 1 min in the presence of 1 μg/ml PI. Phase‐contrast picture were taken. Scale bar, 100 μm.Exosome preparations from colon explants of DSS‐treated *wild‐type* (WT) and *Il1b* ^−/−^ mice were subjected to IL‐1β bioactivity assay. Exosomal proteins concentrated from 10^7^ exosomes were prepared from supernatants. HEK293 cells stably expressing IL‐1R1 were transfected with an NFκB luciferase reporter construct and stimulated with indicated exosome preparations in the presence or absence of anti‐IL‐1β overnight. Cell lysates were analyzed for luciferase activity. *n* = 3 mice. Experiments were repeated twice. Data information: For panels A and F, data were presented as mean ± SEM. ***P < 0.001, ****P < 0.0001 by unpaired two‐tailed *t*‐test. Unless specified, all experiments were repeated three times with consistent results. The representative results are shown. Source data are available online for this figure.

Biologically active IL‐1β was secreted via small extracellular vesicles (sEVs) by young adult mouse colon cells (YAMCs) in response to LPS and ATP stimulation (Fig EV1A), which triggered the formation and release of sEVs containing GSDMD in complex with polyubiquitinated pro‐IL‐1β (Bulek *et al*, 2020). This process was abrogated by the NLRP3 inhibitor MCC950 (Fig EV1B), and attenuated by the caspase 8 inhibitor Z‐IETD‐FMK, but not by the caspase 1 inhibitor Ac‐YVAD‐cmk (Fig EV1C). These findings revealed a poorly understood pathway of exosome biogenesis that is driven by the NLRP3‐caspase 8 inflammasome. Since our previous study had shown that the pore‐forming protein GSDMD plays a critical role in the production of pro‐IL‐1β‐containing sEVs, we sought to further delineate this cellular process by characterizing novel GSDMD‐interacting proteins in YAMCs in response to LPS and ATP stimulation.

**Figure 1:**
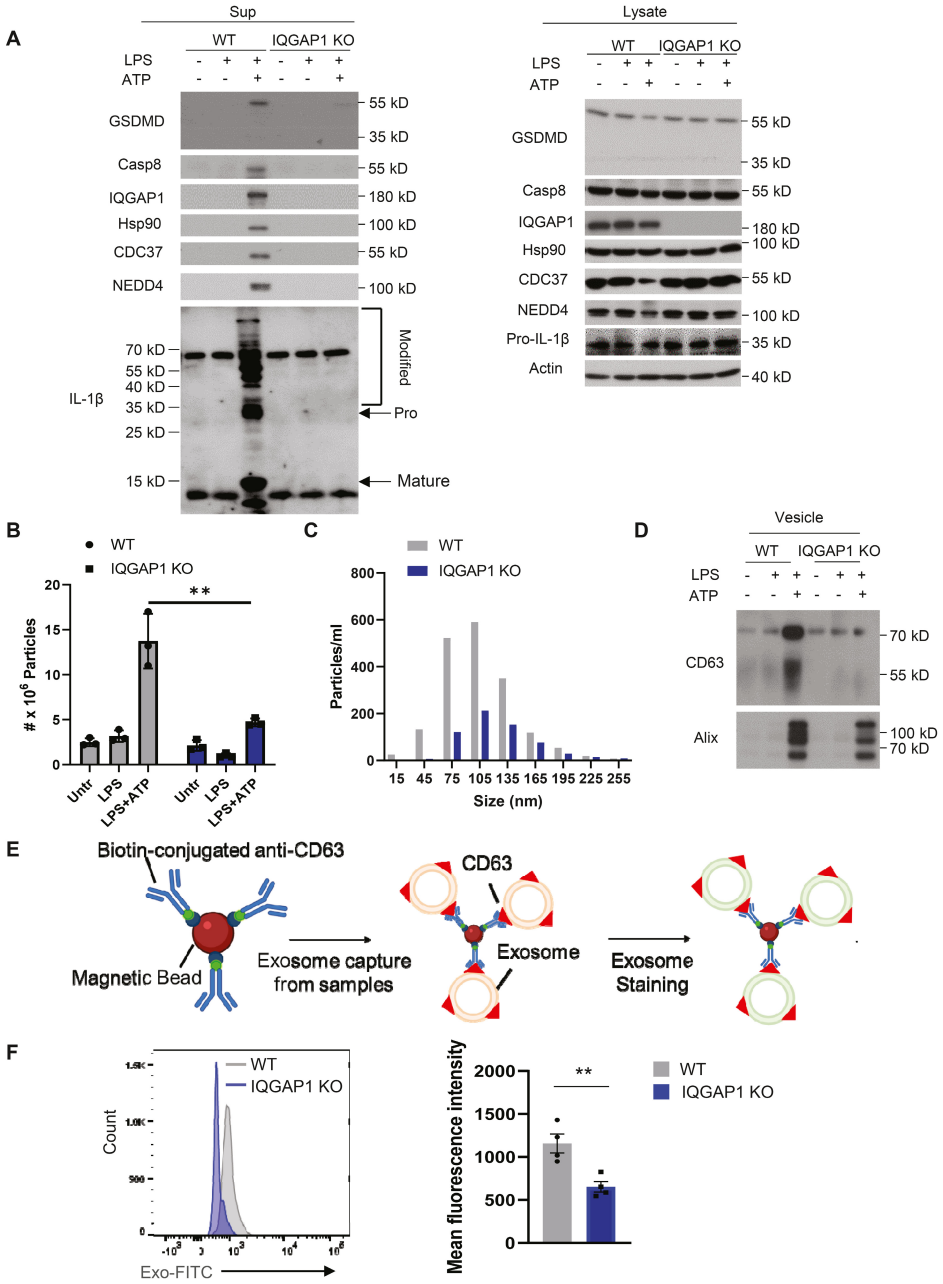
IQGAP1 promotes LPS + ATP‐induced exosome release Western blot analysis of supernatants and whole‐cell lysates collected from WT and IQGAP1 KO YAMC cells with and without LPS stimulation for 4 h or LPS plus ATP (4 h plus 30 min) treatment as indicated.Nanoparticle tracking analysis of supernatant of WT and IQGAP1 KO YAMC cells treated with LPS plus ATP. **P < 0.01 by unpaired two‐tailed *t*‐test (*n* = 3 biological repeats in one experiments).Size distributions for vesicles as analyzed in (B).Western blot analysis of enriched extracellular vesicles from supernatants of WT and IQGAP1 KO YAMC cells.Diagram illustrating the principle of flow cytometry‐based exosome analysis.Flow cytometry analysis for CD63^+^ exosomes from supernatants of LPS plus ATP‐treated WT and IQGAP1 KO supernatants. Quantification was done by mean fluorescence intensity. **P < 0.01 by unpaired two‐tailed *t*‐test (*n* = 4). Data information: For panels (B) and (F), data were presented as mean ± SEM. All experiments were biologically repeated for 3 times and yielded consistent results; the representative results are shown. Source data are available online for this figure.

**Figure EV2:**
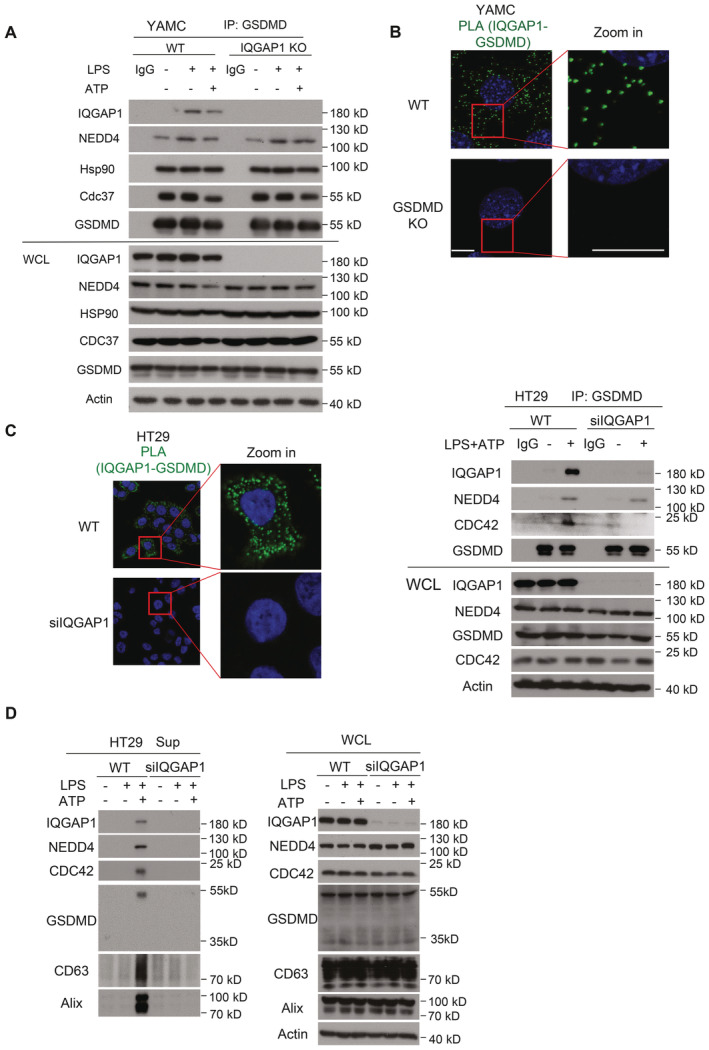
GSDMD interacts with IQGAP1 Endogenous GSDMD were immunoprecipitated from lysates of wild‐type (WT) and IQGAP1‐deficient (IQGAP1 KO) YAMC cells that had been treated as indicated. The precipitates were analyzed by Western blot.Proximity ligation assay (PLA) for IQGAP1‐GSDMD interaction in WT and GSDMD‐deficient (GSDMD KO) YAMC cells after LPS plus ATP treatment. Scale bar, 10 μm.HT‐29 cells were transfected with either scramble or IQGAP1‐targeting siRNA followed by stimulation with LPS for 4 h 48 h post transfection. After LPS stimulation, cells were further exposed to ATP for 30 min. Left panel: After ATP stimulation cells were subjected to PLA for endogenous IQGAP1 and GSDMD. Following PLA, cells were counter stained with DAPI and visualized under confocal microscope. Scale bar: 10 μm. Right panel: Lysates from treated and untreated cells were subjected to coimmunoprecipitation for GSDMD and probed for indicated proteins.Supernatants from cells treated as described in panel (A) were subjected to methanol treatment to precipitate total protein, which were then solubilized with Laemmli buffer and analyzed by Western blot. Data information: All experiments were repeated 3 times and yielded consistent results. The representative results are shown. Source data are available online for this figure.

Multiple lines of evidence support an interaction of IQGAP1 and GSDMD in multiple cell types. Our proteomic profiling study of LPS and ATP‐stimulated YAMCs showed IQGAP1 was an interacting partner of GSDMD (Bulek *et al*, 2020). Immunoprecipitation of GSDMD in cultured immortalized mouse bone marrow derived macrophages showed IQGAP1 was part of the bound protein complex (Gao *et al*, 2021). Similarly, we found that immunoprecipitation of GSDMD using lysates of LPS‐stimulated YAMCs also showed IQGAP1 was a major binding partner (Table EV1). Furthermore, we detected an interaction between IQGAP1 and GSDMD by proximity ligation assay in YAMCs (Fig EV2A and B) and HT29 cells (Fig EV2C).

Given the potential interaction of IQGAP1 and GSDMD, we tested if IQGAP1 functionally regulated the presence of the GSDMD complex in exosomes. To address this question, we subjected IQGAP1 deficient and wild‐type cells to LPS and ATP stimulation and analyzed exosome production as well as protein expression from treated and untreated cells. Analysis of exosome immunoblots showed that IQGAP1 deficiency was associated with complete loss of detection of (i) chaperoned GSDMD (in a complex with Hsp90‐CDC37), (ii) NEDD4, a ubiquitin E3 ligase for IL‐1β, and (iii) polyubiquitinated pro‐IL‐1β in the supernatant (Fig 1A). As a control, all of the complex member proteins were detected in the lysates of both the WT and KO cell lines with the exception of IQGAP1 which is absent in the KO line (Fig 1A). Consistently, nanoparticle tracking analysis (NTA) showed that IQGAP1 deficiency substantially reduced the release of vesicles ranging from 45 to 165 nm in diameter (Fig 1B and C). Importantly, these LPS + ATP‐induced, GSDMD‐ and IQGAP1‐dependent vesicles were positive for the endosomal markers CD63 and ALIX (Odorizzi, 2006; van Niel *et al*, 2011; Greening *et al*, 2015; Fig 1D), indicating their identity as exosomes. This IQGAP1‐dependent release of GSDMD, ALIX, NEDD4 and CD63 was further validated in HT29 cells (Fig EV2D).

To further quantify the abundance of released exosomes, we employed a flow‐cytometry based assay where CD63 positive vesicles were first captured by biotinylated anti‐CD63 antibody and streptavidin‐coated magnetic beads before being analyzed by flow cytometry (Fig 1E). Consistent with the reduced particle counts and diminished CD63 in the supernatant, flow cytometry analysis confirmed that, in response to LPS and ATP stimulation, IQGAP1‐deficient cells' exosome release was substantially reduced (Fig 1F). Taken together, the data indicate that IQGAP1 is a GSDMD‐interacting protein required for the production of GSDMD‐dependent pro‐IL‐1β‐containing exosomes.

### The IQ domain of IQGAP1 interacts with the C‐terminal domain of full‐length GSDMD

**Figure 2:**
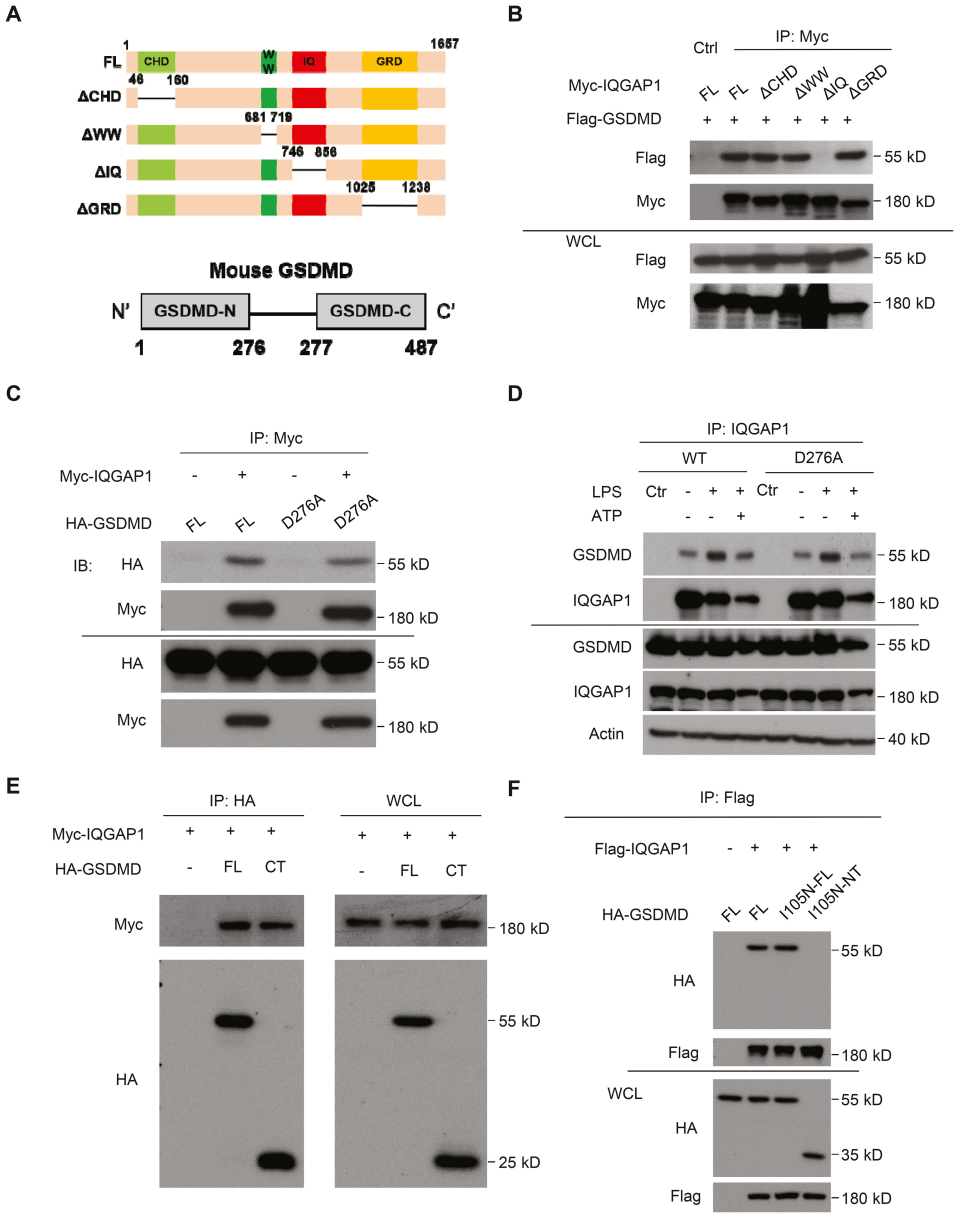
The IQ domain of IQGAP1 interacts with the C‐terminal domain of GSDMD Schematics of domain structure of IQGAP1 and GSDMD.Coimmunoprecipitation of GSDMD with full‐length and deletion mutants of IQGAP1 tested in HEK293 cells.Coimmunoprecipitation of GSDMD cleavage mutant D276A with IQGAP1 overexpressed in HEK293 cells.Coimmunoprecipitation of GSDMD with IQGAP1 in WT and cleavage mutant (D276A) restored GSDMD KO YAMC cells. Cells were treated as indicated.Coimmunoprecipitation of C‐terminal GSDMD (CT) with IQGAP1 overexpressed in HEK293 cells.Coimmunoprecipitation of pyroptosis mutants full‐length (I105N FL) and N‐terminal GSDMD defective of pyroptosis (I105N‐NT) with IQGAP1. Data information: All experiments were repeated 3 times and yielded consistent results; the representative results are shown. Source data are available online for this figure.

IQGAP1 is a large scaffold protein involved in the regulation of actin cytoskeleton, cell migration and movement of intracellular vesicles. Structurally, IQGAP1 contains defined structural domains of well‐characterized functions, consisting of a calponin homology domain (CHD), a WW domain, an IQ domain and a rasGAP‐related domain (GRD) (Fig 2A). On the other hand, GSDMD is comprised of an N‐terminal domain (p30) and a C‐terminal domain, joined by a linker sequence containing caspase cleavage sites (Fig 2A).

To further characterize the IQGAP1‐GSDMD complex, we performed structure function analysis to identify the domains that mediate this interaction. We first generated a set of deletion mutants of IQGAP1 which lacked CHD, WW, IQ, or GRD and tested their abilities to interact with full‐length GSDMD in co‐immunoprecipitation assay. Deletion of CHD, WW or GRD did not affect the formation of the IQGAP1‐GSDMD complex, whereas removal of 4 IQ motifs (IQ domain, residues 745–856 in mouse IQGAP1) abolished the interaction (Fig 2B). This indicated that the IQ domain was required for the interaction between IQGAP1 and GSDMD.

Next, we sought to map the GSDMD domain that interfaces with IQGAP1. Consistent with our previous report showing the cleavage of GSDMD was not required for its role in mediating the biogenesis of inflammasome‐induced exosomes, mutant GSDMD resistant to caspase cleavage (GSDMD D276A) was readily co‐precipitated with IQGAP1 (Fig 2C and D). Further analysis showed that the C‐terminal GSDMD was sufficient for the interaction with IQGAP1 (Fig 2E). Overexpression of N‐terminal GSDMD leads to pyroptotic cell death, which would prevent structure–function analysis of its ability to bind IQGAP1.

To investigate whether N‐GSDMD contributes to the interaction, we introduced a point mutation (I105N) to abolish GSDMD oligomerization, which negates the pyroptotic activity of N‐GSDMD (Kayagaki *et al*, 2015). GSDMD I105N showed comparable capacity in mediating the release of poly‐ubiquitinated pro‐IL‐1β (Fig EV1D), indicating that the pyroptotic activity of GSDMD is dispensable for exosomal IL‐1β release. Consistently, the production of polyubiquitinated pro‐IL‐1β did not associate with an increased in propidium iodide (PI) (Fig EV1E), a finding in line with our previous report (Bulek *et al*, 2020). Consistent with the functional data, full‐length GSDMD I105N co‐precipitated with IQGAP1 (Fig 2F). This interaction was abolished by the deletion of the C‐terminal domain, confirming the C‐terminal GSDMD was sufficient and necessary for the IQGAP1‐GSDMD interaction (Fig 2E and F). Thus, the data collectively showed that the IQ domain of IQGAP1 interacts with the C‐terminal domain of full‐length GSDMD.

### The IQGAP1‐GSDMD interaction mediates LPS + ATP‐induced exosomal IL‐1β release

**Figure 3:**
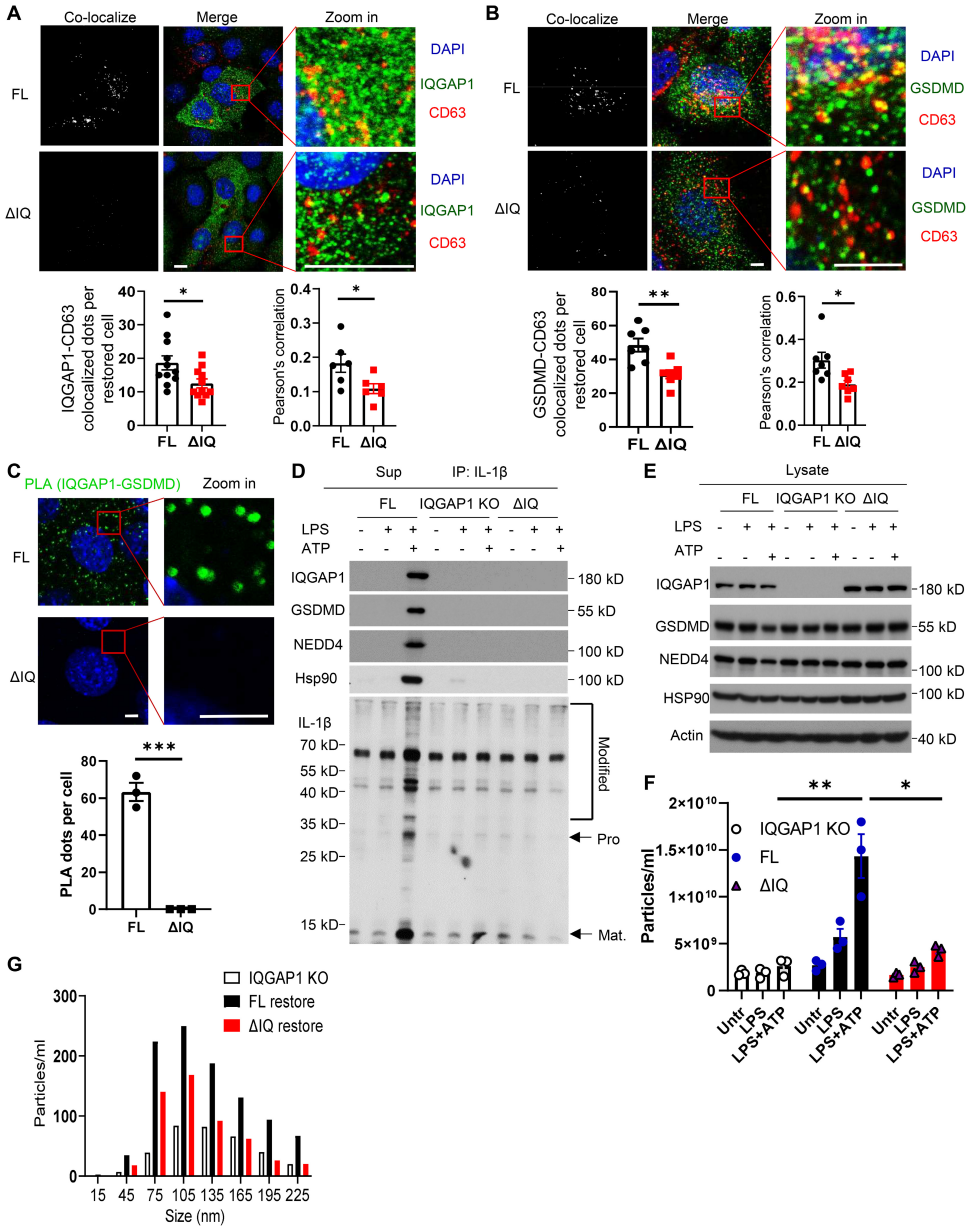
The IQ domain of IQGAP1 is required for LPS + ATP‐induced exosome release Imaging analysis for colocalization of CD63 with IQGAP1 in IQGAP1 (FL) and IQ domain truncation (ΔIQ) restored YAMC cell. Cells were treated with LPS plus ATP. The IQGAP1‐CD63 colocalization dots (shown by yellow) were counted, and Pearson's correlation coefficient was calculated for the quantification of colocalization. Data were presented as mean ± SEM. *P < 0.05 by unpaired two‐tailed student's *t*‐test. Scale bar, 10 μm.Imaging analysis for colocalization of CD63 with GSDMD in IQGAP1 full length (FL) and IQ domain truncation restored YAMC cell (ΔIQ) after LPS plus ATP treatment. The GSDMD‐CD63 colocalized dots were counted, and Pearson's correlation coefficient was calculated for quantification. Data were presented as mean ± SEM. *P < 0.05; **P < 0.01 by unpaired two‐tailed student's *t*‐test. Scale bar, 5 μm.Proximity ligation assay for IQGAP1 and GSDMD in IQGAP1 FL restored cells and ΔIQ restored cells. Data were presented as mean ± SEM. ***P < 0.001 by unpaired two‐tailed *t*‐test. Scale bar, 5 μm.Western blots analysis for supernatants collected from IQGAP1 KO YAMC cells, IQGAP1 full length, and IQ domain truncation restored IQGAP1 KO YAMC cells. Cells were treated as indicated, and the supernatants were subjected to IP with anti‐IL‐1β.Western blots analysis for whole‐cell lysate collected from panel D.ZetaView particle analysis for exosomes concentrated from supernatants collected from LPS or LPS and ATP‐treated cells as indicated. Data were presented as mean ± SEM. *P < 0.05; **P < 0.01 by unpaired two‐tailed student's *t*‐test.Vesicle size distributions for exosome samples collected from panel F. Data information: All experiments were biologically repeated for 3 times and yielded consistent results; the representative results are shown. Source data are available online for this figure.

Next, we sought to determine whether abrogation of the IQGAP1 and GSDMD interaction was sufficient to ablate LPS + ATP‐induced production of GSDMD‐dependent pro‐IL‐1β‐containing sEVs. Interestingly, both full‐length IQGAP1 and GSDMD showed co‐localization with CD63 positive puncta (late endosomes) in LPS and ATP‐stimulated cells (Fig 3A and B), suggesting that both proteins were distributed on the late endosomes. Interestingly, deletion of IQ motif appeared to redistribute IQGAP1 away from CD63 positive puncta (Fig 3A and B). In IQGAP1‐deficient cells restored with ΔIQ IQGAP1, the co‐localization between GSDMD and CD63 was also noticeably reduced (Fig 3A and B). The data suggest that interaction with IQGAP1 might be required for GSDMD to localize to the late endosome.

To determine the role of IQGAP1‐GSDMD interaction in exosome production, we restored IQGAP1‐deficient YAMC cells with an empty vector, a wild‐type full‐length IQGAP1 cDNA (FL) or mutant IQGAP1 with deletion in its IQ domain (ΔIQ). Consistent with a loss of interaction with GSDMD (Figs 2B and 3C), cells restored with ΔIQ IQGAP1 did not release chaperoned GSDMD complex or ubiquitinated pro‐IL‐1β into the supernatant (Fig 3D and E). Furthermore, nanoparticle tracking analysis showed that ΔIQ IQGAP1 failed to restore LPS + ATP‐induced release of extracellular vesicles (Fig 3F and G). Taken together, the data suggest that the IQGAP1‐GSDMD interaction is required for LPS + ATP‐induced exosomal IL‐1β release.

### IQGAP1 recruits Tsg101 to engage the ESCRT system for exosomal IL‐1β release

**Figure 4:**
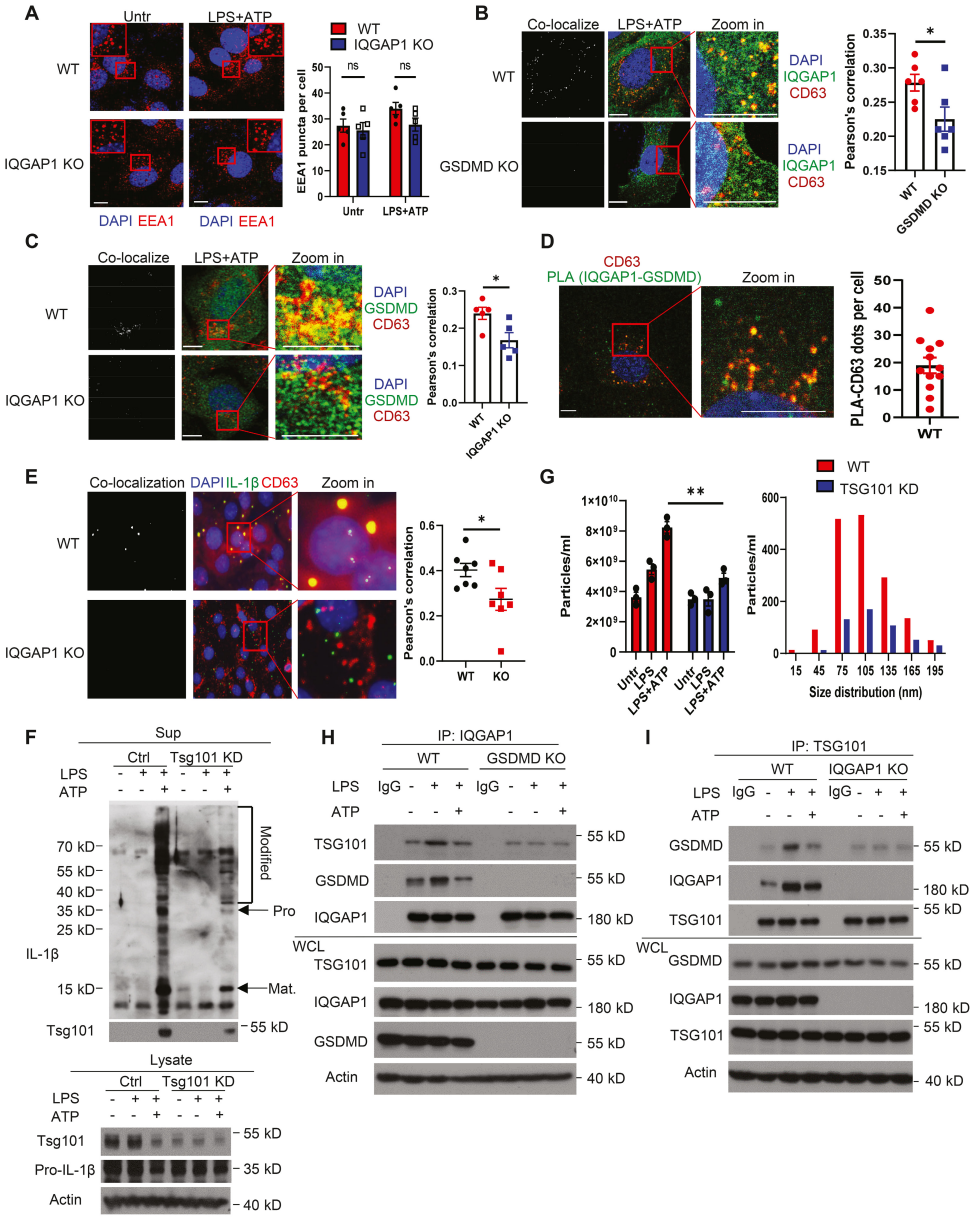
IQGAP1 recruits Tsg101 to GSDMD+ late endosome to drive exosomal IL‐1β release Imaging analysis for early endosome (shown by marker EEA1) change in WT and IQGAP1 KO YAMC cells upon indicated treatments. Quantification was done by counting the EEA1 puncta from each cell. Five field views of cells' puncta were counted. Three independent experiments were repeated, and data were shown as mean ± SEM. Ns denotes not significant by unpaired two‐tailed *t*‐test. Scale bar, 10 μm.Colocalization of CD63 and IQGAP1 in YAMC and GSDMD KO YAMC cells. Yellow dots indicated the co‐localization signal. Data were presented as mean ± SEM. *P < 0.05 by two tailed unpaired *t*‐test. Scale bar, 10 μm.Colocalization of CD63 and GSDMD in YAMC and IQGAP1 KO YAMC cells. Data were presented as mean ± SEM. *P < 0.05 by two tailed unpaired *t*‐test. Scale bar, 10 μm.Colocalization of CD63 with IQGAP1‐GSDMD complex, as measured by PLA, shown by the yellow dots after LPS and ATP treatment.Colocalization of CD63 and pro‐IL‐1β in YAMC and IQGAP1 KO YAMC cells. Scale bar, 10 μm. Data were shown as mean ± SEM. *P < 0.05 by two tailed unpaired *t*‐test.Western blot analysis of extracellular vesicle secretion from WT and TSG101 knockdown cells upon indicated treatments. Secreted proteins were pulled down by IL‐1β in the supernatant and the indicated proteins were probed for.Total particle count and size distribution for exosomes collected from WT and TSG101 knockdown cell supernatants with indicated treatment. Data were presented as mean ± SEM. *P < 0.05 by two tailed unpaired *t*‐test.Western blot analysis for interaction of TSG101 with IQGAP1 in WT and GSDMD KO cells by pulling down IQGAP1 under indicated treatments.Coimmunoprecipitation of GSDMD with TSG101 in WT and IQGAP1 KO cells by pulling down TSG101 under indicated treatments. Data information: All experiments were biologically repeated for 3 times and yielded consistent results; the representative results are shown. Source data are available online for this figure.

Our observation thus far led us to ask how GSDMD and IQGAP1 act in concert to promote exosomal IL‐1β release. We have previously shown that LPS stimulation instigates the Hsp90‐CDC37 chaperoned GSDMD protein to recruit the E3 ligase NEDD4 (Bulek *et al*, 2020), a critical biochemical event required for the biogenesis of GSDMD‐dependent exosomes. Notably, IQGAP1 deficiency did not impair the formation of the chaperoned GSDMD complex or the recruitment of NEDD4 (Fig EV1A), suggesting that the IQGAP1 is unlikely to participate in these upstream events. In addition, IQGAP1 deficiency did not reduce the number of EEA1+ early endosomes in LPS‐stimulated YAMC cells (Fig 4A). Instead, our imaging analysis showed both IQGAP1 and GSDMD localized to CD63 positive puncta in response to LPS and ATP stimulation and their distribution to late endosome were also mutually dependent (Fig 4B and C). In addition, proximity ligation analysis indicated that the IQGAP1‐GSDMD interaction occurred at CD63 positive late endosomes (Fig 4D). Furthermore, pro‐IL‐1β also localized to CD63 puncta in LPS and ATP‐stimulated cells in an IQGAP1‐dependent manner (Fig 4E). Collectively, the evidence alludes to the late endosome as the site of regulation where GSDMD and IQGAP1 coordinate to promote exosomal IL‐1β release.

**Figure EV3:**
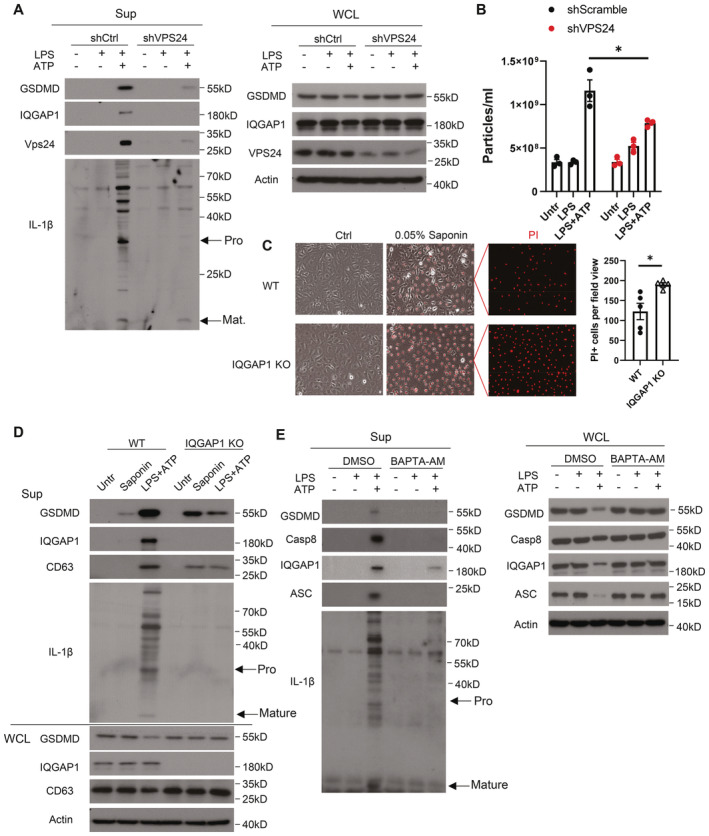
ESCRT is required for exosomal pro‐IL‐1β release YAMCs were transfected with either scramble or Vps24‐targeting siRNA followed by stimulation with LPS for 4 h 48 h post transfection. After LPS stimulation, cells were further exposed to ATP for 30 min. Supernatants from cells treated described in panel (A) were subjected to methanol treatment to precipitate total protein, which were then solubilized with Laemmli buffer and analyzed by Western blot.Nanoparticle tracking analysis of supernatant of scramble siRNA or Vps24 targeting siRNA‐transfected YAMC cells treated with LPS plus ATP. Data were presented as mean ± SEM *P < 0.05 by unpaired two‐tailed *t*‐test.WT and IQGAP1 KO YAMC cells were left unstimulated and treated with 0.05% saponin for 1 min in phenol red‐free DMEM supplemented with 1 μg/ml PI. Five field views were counted. Data were shown as mean ± SEM. *P < 0.05 by unpaired two‐tailed *t*‐test. Scale bar: 100 μm.Western blot analysis of supernatants and whole‐cell lysates collected from YAMC cells treated with 0.05% saponin (for 1 min) or LPS (4 h) plus ATP (30 min).Western blot analysis of supernatants and whole‐cell lysates collected from YAMC cells with and without LPS stimulation for 4 h or LPS plus ATP (4 h plus 30 min) in the presence or absence of BAPTA‐AM (6 μM). Data information: All experiments were repeated 3 times and yielded consistent results. The representative results are shown. Source data are available online for this figure.

A key step for exosome biogenesis at the late endosome in the classical model is the activation of the ESCRT system. In particular, Tsg101, a component of ESCRT‐1, plays a vital role in recognizing cargo proteins and initiating membrane remodeling. This ultimately promotes maturation of MVBs via the invagination of late endosomal membrane that gives rise to ILVs (Razi & Futter, 2006; Colombo *et al*, 2013). In our system, Tsg101 knockdown greatly reduced LPS + ATP‐induced release of polyubiquitinated pro‐IL‐1β and extracellular vesicles (Fig 4F and G), suggesting this GSDMD‐ and IQGAP1‐dependent exosomal IL‐1β release relies on the ESCRT system. Notably, IQGAP1 has been previously shown to bind to Tsg101 (Morita *et al*, 2007; Dolnik *et al*, 2014). We did, indeed, detect enhancement of IQGAP1‐Tsg101 complex formation in response to LPS stimulation and Tsg101 readily pulled down endogenous GSDMD (Fig 4H and I). Furthermore, knockdown of Vps24, a component of ESCRT‐3 that catalyzes the last step of membrane remodeling during ILV formation, suppressed the release of polyubiquitinated pro‐IL‐1β and extracellular vesicles (Fig EV3A and B), further demonstrating a critical role of the ESCRT system in this process.

In addition to catalyzing exosome biogenesis, the ESCRT system also plays a fundamental role in plasma membrane repair (Jimenez *et al*, 2014). A recent study directly implicated IQGAP1 as a key adaptor protein required for ESCRT‐dependent maintenance of plasma membrane integrity (Claude‐Taupin *et al*, 2021). Consistent with this prior report, unstimulated IQGAP1‐deficient YAMCs appeared more sensitive to plasma membrane damage induced by low‐dose saponin, as shown by increased PI uptake and LDH release compared to wild‐type cells (Fig EV3C). Notably, the membrane damage induced by low dose saponin did not lead to the release of polyubiquitinated pro‐IL‐1β in unstimulated YAMCs (Fig EV3D). This result underscores the fact that the exosomal pro‐IL‐1β release, driven by NLRP3‐inflammasome activation, is an active process involving cargo protein selection, rather than a passive discharge of cytoplasmic contents. Furthermore, the data suggest that LPS and ATP stimulation is required to engage the ESCRT system for exosome biogenesis. In support of this notion, chelation of intracellular Ca^2+^, which triggers ESCRT activation (Scheffer *et al*, 2014), limited LPS and ATP‐induced exosome production as well as the release of polyubiquitinated pro‐IL‐1β and GSDMD (Fig EV3E). Taken together, the data support a model where the IQGAP1‐GSDMD complex recruits Tsg101 to engage the ESCRT system for exosomal IL‐1β release.

### GSDMD sustains LPS‐induced CDC42 to promote sEVs production

**Figure 5:**
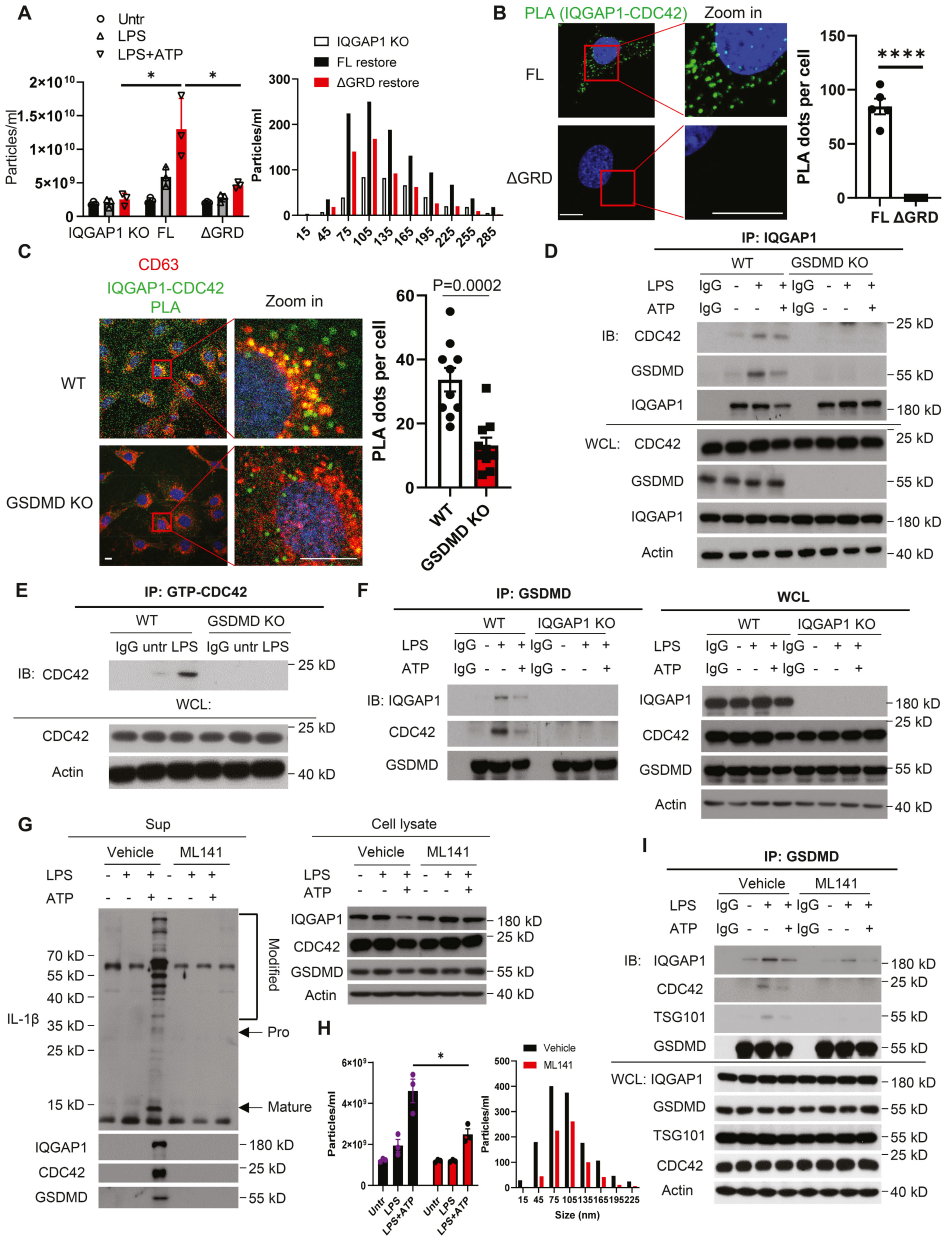
Active CDC42 promotes IQGAP1‐ and GSDMD‐mediated exosomal IL‐1β release Total particle count and size distribution of exosomes secreted in the supernatant from IQGAP1 KO (IQ1 KO), IQGAP1 full‐length restored (FL), and GRD domain truncation restored (ΔGRD) YAMC cells upon LPS or LPS plus ATP treatment. Data were presented as mean ± SEM. *P < 0.05 by two‐tailed unpaired *t*‐test.PLA assay for IQGAP1 and CDC42 interaction in IQGAP1 FL restored cell and ΔGRD restored cell. The green dots showed the interaction of IQGAP1 and CDC42. Data were presented as mean ± SEM. ****P < 0.0001 by unpaired two‐tailed *t*‐test. Scale bar, 10 μm.Colocalization of CD63 with IQGAP1‐CDC42 complex (shown by PLA signal) in WT and GSDMD KO cells under LPS treatment. Quantification was performed by counting PLA green dots. Data were presented as mean ± SEM. Two‐tailed unpaired *t*‐test was used to calculate the *P* value. Scale bar, 10 μm.Coimmunoprecipitation analysis of endogenous IQGAP1 with CDC42 in wild‐type and GSDMD KO cells upon LPS or LPS plus ATP treatment.Coimmunoprecipitation analysis of GTP‐bound CDC42 in indicated cells following LPS treatment.Coimmunoprecipitation analysis of endogenous GSDMD with CDC42 in wild‐type and IQGAP1 KO cells upon LPS or LPS plus ATP treatment.Western blots for exosome secretion upon CDC42 GTPase inhibition. Cells were pretreated with and without 10 μM ML141 for 24 h to fully block its GTPase activity, followed by LPS and ATP treatment as indicated.Particle analysis and size distribution for exosomes collected from cells with the same treatment as panel (G). Data were presented as mean ± SEM. *P < 0.05 by unpaired two‐tailed *t*‐test.Coimmunoprecipitation analysis of endogenous GSDMD with CDC42 in vehicle or ML141 (10 μM for 24 h) treated cells following LPS or LPS plus ATP treatment. Data information: All experiments were biologically repeated for 3 times and consistent results were yielded. The representative results are shown. Source data are available online for this figure.

While our data thus far showed IQGAP1 may serve as a bridge that connects GSDMD and the inflammasome to the ESCRT system, it remains unclear how IQGAP1 is activated in this process. The subcellular localization and effector function of IQGAP1 is regulated by Rho family GTPases that bind to its GRD domain (White *et al*, 2012). We found that although the GRD domain was dispensable for the IQGAP1‐GSDMD interaction (Fig 2B), it was required for IQGAP1 to mediate LPS + ATP‐induced exosome production (Fig 5A), indicating that a Rho GTPase plays a critical role in IQGAP1‐mediated exosome production.

Through a screening using co‐immunoprecipitation, we identified CDC42 as the Rho GTPase which formed a complex with IQGAP1 in LPS and ATP treatment. Proximity ligation assay subsequently validated the formation of IQGAP1‐CDC42 complex in a GRD‐dependent manner in LPS + ATP‐ treated YAMC cells (Fig 5B). Notably, the IQGAP1‐CDC42 complex predominantly localized to CD63 positive puncta (Fig 5C), where GSDMD was found to interact with IQGAP1 (Fig 4D). This data hinted at a possible connection between GSDMD and the IQGAP1‐CDC42 complex formed in response to LPS + ATP stimulation.

Indeed, GSDMD‐deficiency substantially reduced the interaction between IQGAP1 and CDC42 (Fig 5C and D), revealing a critical role of GSDMD in promoting the IQGAP1‐CDC42 interaction. Of note, CDC42, like all other small GTPases, adopts an active conformation only when it is bound to GTP but not GDP (Jaffe & Hall, 2005); and IQGAP1 preferentially interacts with the GTP‐bound, active CDC42 (White *et al*, 2012). Interestingly, while GSDMD co‐precipitated with CDC42 in an IQGAP1‐dependent manner, LPS stimulation elevated the abundance of GTP‐bound, active CDC42 in a GSDMD‐dependent manner (Fig 5E and F). This suggests a functionally critical interplay among GSDMD, IQGAP1 and GTP‐bound CDC42. Because the abrogation of the IQGAP1‐CDC42 interaction abolished GSDMD‐dependent exosome production (Fig 5A), we posited that the active, GTP‐bound CDC42 is vital to the GSDMD‐dependent biogenesis of IL‐1β‐containing exosomes.

To test this, we employed a non‐competitive reversible CDC42 inhibitor to transiently depress the level of GTP‐bound CDC42 and examined its impact on LPS and ATP‐induced exosomal IL‐1β release (Hong *et al*, 2013). Pharmacological suppression of active CDC42 level decreased the production of GSDMD‐ and pro‐IL‐1β‐containing exosomes in YAMC cells (Fig 5G and H). It also disrupted the recruitment of TSG101 to the GSDMD‐IQGAP1 complex (Fig 5I). These results considered, the data suggest that CDC42 serves as a molecular switch and enables IQGAP1 to bridge GSDMD to TSG101 during inflammasome‐triggered exosome biogenesis.

### IQGAP1 drives GSDMD‐dependent exosomal IL‐1β release *in vivo*

**Figure 6:**
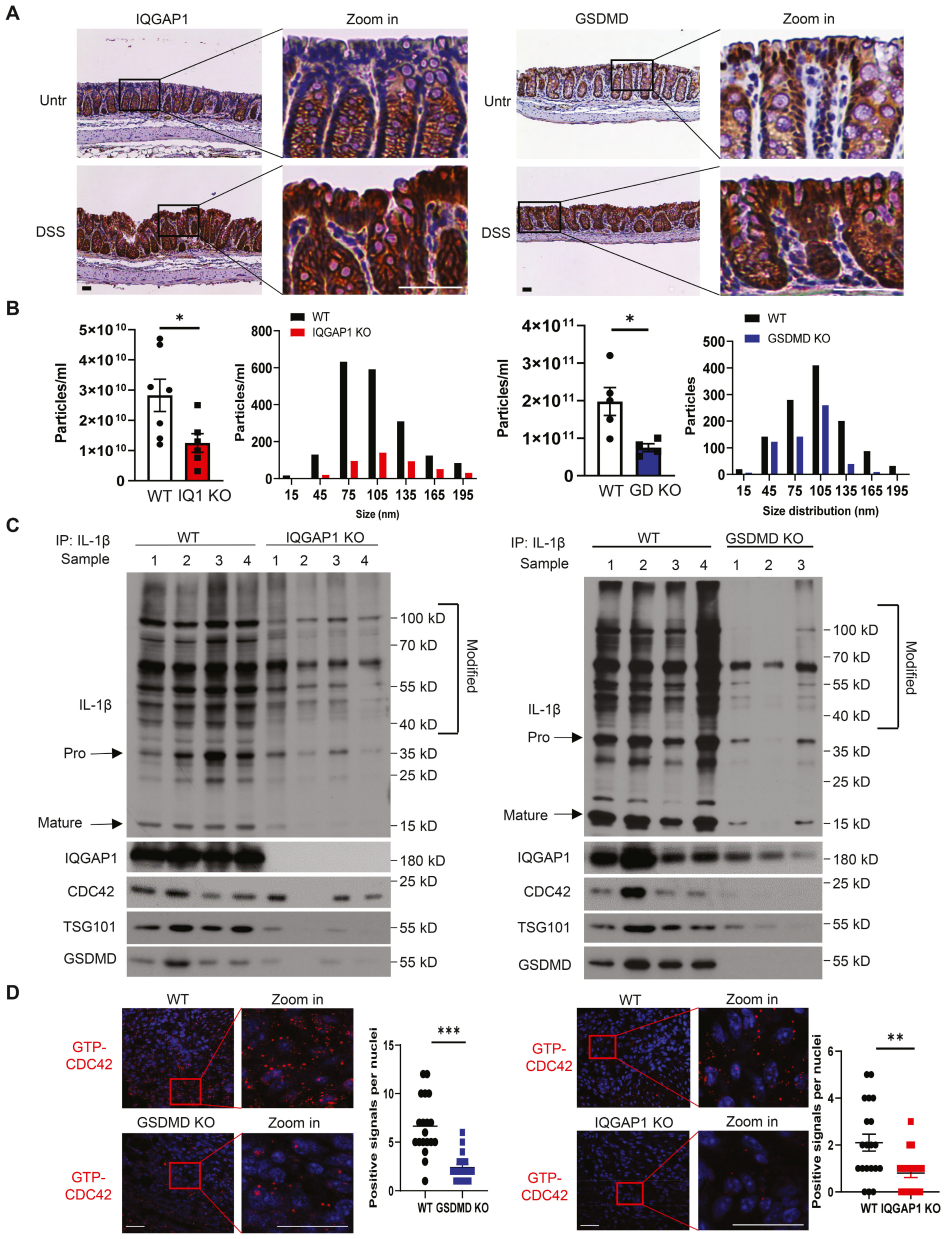
IQGAP1 drives GSDMD‐dependent exosomal IL‐1β release *in vivo* Histology staining for IQGAP1 and GSDMD in naive and inflamed (3 days of DSS‐challenge) colon tissues. Scale bar, 100 μm.Total particle count and size distribution for exosomes collected from colon explant of DSS‐treated IQGAP1 or GSDMD‐knockout mice and their respective controls. *n* = 5–7. Data were presented as mean ± SEM. *P < 0.05 by two‐tailed unpaired *t*‐test.Western blot analysis for supernatant collected from colon explants of DSS‐treated *wild‐type* (WT), *Gsdmd* ^−/−^ (GSDMD KO) and Iqgap1^−/−^ (IQGAP1 KO) mice. Supernatant was first pulled down by IL‐1β antibody, and samples were subjected to Western blot analysis. The indicated proteins were probed.Immunofluorescent staining with anti‐GTP‐CDC42 in DSS‐treated colons from *Gsdmd* ^+/+^ (WT) and *Gsdmd* ^−/−^ (GSDMD KO), *IQGAP1* ^+/+^ and *IQGAP1* ^−/−^ mice. Data were presented as mean ± SEM. **P < 0.01; ***P < 0.001 by unpaired two‐tailed *t*‐test. Data information: All experiments were repeated twice with consistent results. Representative results are shown. Source data are available online for this figure.

We have previously reported GSDMD is highly expressed in IECs during intestinal inflammation. In addition, we have reported IEC‐derived GSDMD mediates the release of pro‐IL‐1β containing exosomes from inflamed colon explants from dextran sulfate sodium (DSS)‐treated mice (Bulek *et al*, 2020). The pro‐IL‐1β containing exosomes produced by colon explants contain biologically active IL‐1β (Fig EV1F). Immunohistochemistry analysis showed both IQGAP1 and GSDMD were highly expressed in inflamed IECs (Fig 6A). Importantly, colon explants from DSS‐treated *Iqgap1* ^−/−^ mice had a major reduction in exosome production compared to that from DSS‐treated control mice, a phenotype reminiscent of the *Gsdmd* ^−/−^ mice (Fig 6B). Western analyses also revealed a substantial decrease in polyubiquitinated IL‐1β, GSDMD, CDC42, TSG101 in IQGAP1‐ deficient colon explants from DSS‐treated mice (Fig 6C). Consistently, colon explant‐derived GSDMD‐dependent exosomes from DSS‐treated mice contained high levels of IQGAP1, CDC42 and TSG101 (Fig 6C). Furthermore, the abundance of GTP‐bound CDC42, as detected by a conformation‐specific antibody, was noticeably reduced in the GSDMD‐ and IQGAP1‐deficient colon from DSS‐treated mice compared to that from the wild‐type (Fig 6D). Collectively, these results indicate that, through the activation of CDC42 and the involvement of the ESCRT system, IQGAP1 plays an essential role in driving GSDMD‐dependent exosomal IL‐1β release *in vivo*.

## Discussion

Through a non‐biased proteomic profiling, we identified IQGAP1 as an interacting partner of GSDMD in IECs. IQGAP1 deficiency abolished LPS and ATP‐induced release of pro‐IL‐1β‐ and GSDMD‐containing extracellular vesicles from endosomal origin, phenocopying the GSDMD‐deficient cells. Structure function analysis showed that while the C‐terminal domain of GSDMD was sufficient for the interaction with IQGAP1, the IQGAP1‐GSDMD interaction relied on the IQ domain of IQGAP1. Functionally, abrogation of the IQGAP1‐GSDMD interaction reduced the production of exosomes and diminished the trafficking of both IQGAP1 and chaperoned GSDMD complex to late endosomes. Mechanistically, the GRD domain in IQGAP1 bound to the active CDC42 in response to LPS stimulation, enabling ESCRT to facilitate the release of exosomal IL‐1β. In support of this, transient inhibition of GTP‐bound active CDC42 also reduced the release of exosomes. Importantly, this IQGAP1‐directed release of GSDMD‐dependent exosomes were detected from the inflamed colon. Taken together, the study reveals IQGAP1 as a link between inflammasome activation and GSDMD‐dependent, ESCRT‐mediated exosomal release of IL‐1β.

Information gained from this study is illuminative for understanding stimulus‐induced, selective packing of cytosolic proteins during exosome biogenesis. We propose a revision to the classical model of exosome biogenesis based on these new findings: while LPS stimulation induces the chaperoned GSDMD to engage the caspase 8 inflammasome and pro‐IL‐1β (Bulek *et al*, 2020), LPS also activates the small GTPase CDC42 to form a complex with IQGAP1. Small GTPases, like CDC42, often function as a molecular switch. The active GTP‐bound form of CDC42 triggers conformational changes in IQGAP1, which may allow IQGAP1 to function as a scaffold bridging GSDMD and the associated inflammasome complex to Tsg101. Tsg101 is part of the ESCRT system that recruits poly‐ubiquitinated cargo proteins to late endosome during invagination (Raiborg & Stenmark, 2002; Blot *et al*, 2004). Consistently, we found that ablation of IQGAP1 prevented IL‐1β from relocating to CD63^+^ vesicles. Taken collectively, our findings reveal a crucial role for IQGAP1 in mediating the activation of ESCRT‐mediated cargo loading and membrane remodeling and packaging GSDMD and associated inflammasome into exosome precursor.

Prior reports showed that ESCRT‐dependent membrane repair negatively regulates pyroptosis downstream of GSDMD activation, and inhibition of ESCRT function enhances the non‐selective discharge of cellular contents including IL‐1β in macrophages (Rühl *et al*, 2018; Claude‐Taupin *et al*, 2021). In our study, we found that abrogation of IQGAP1 or knockdown of key ESCRT components blocked the release of exosomes containing GSDMD and pro‐IL‐1β in YAMCs. One important distinction between our experimental system and that described in the earlier reports is the type of stimuli used to engage the ESCRT system. Studies that focused on the repair function of ESCRT employed LPS transfection to activate caspase‐11, which cleaves GSDMD and prompts pore formation on the plasma membrane, leading to Ca^2+^ influx and ESCRT engagement at the plasma membrane. Instead, we used LPS and ATP to activate NLRP3 inflammasome, mobilizing intracellular Ca^2+^ from the ER and recruiting the ESCRT system to late endosome via IQGAP1. The differences in cell types and stimuli, which dictate how and where the ESCRT system is engaged, likely account for the divergent observations.

Our study has identified CDC42 as an important factor in driving inflammasome‐induced exosome release. Curiously, Rho family small GTPases, including CDC42, are widely implicated in inflammasome activation in barrier epithelial cells and often targeted by pathogens, especially at mucosal surfaces (Müller *et al*, 2010). Additionally, IQGAP1 is a target protein for the intracellular pathogen *Yersinia pestis*, whose virulent factor YoM binds to IQGAP1 and suppresses inflammasome activation (Chung *et al*, 2014). Furthermore, accumulating evidence suggests that re‐organization of the microtubules accompanies and enables the NLRP3 inflammasome activation (Misawa *et al*, 2013; Magupalli *et al*, 2020). Since, IQGAP1 and CDC42 are both intimately linked to the remodeling of cytoskeleton and intracellular trafficking, our findings also underscore a potential nexus between inflammasome components and machineries governing vesicle trafficking.

Of note, the calcium‐responsive protein calmodulin has been shown to inhibit the formation of IQGAP1‐CDC42 complex by directly binding to IQGAP1 in response to elevated intracellular Ca^2+^ (Joyal *et al*, 1997; Ho *et al*, 1999). We found that Ca^2+^ chelation indeed reduced the production of poly‐ubiquitinated pro‐ IL‐1β, implicating a critical role for Ca^2+^ signaling in the process. Intriguingly, calmodulin also binds to the IQ motif in IQGAP1 (Rhoads & Friedberg, 1997), the region required for the formation of IQGAP1‐GSDMD complex. Considering that ATP stimulation induces a transient spike followed by an oscillation in intracellular Ca^2+^ concentration (Evans & Sanderson, 1999), it is reasonable to speculate that calmodulin may dynamically modulate the formation of GSDMD‐IQGAP1‐CDC42 complex in tune with Ca^2+^ oscillation, which may be required to coordinate the exosome biogenesis with vesicle trafficking. However, whether and how calmodulin indeed participates in the LPS and ATP induced exosome biogenesis requires further investigation.

The data reported here also provide a potential explanation for how cytosolic proteins may be specifically distributed to the late endosome for subsequent inclusion in exosomes. Both IQGAP1 and GSDMD have been shown, however, to exhibit affinity towards phospholipids such as phosphatidylinositol 4,5‐bisphosphate (PIP2) and PIP3, admittedly in biological processes distinct from exosome biogenesis (Choi *et al*, 2013, 2016; Choi & Anderson, 2016; Liu *et al*, 2016). It is possible that LPS and ATP stimulation may activate the lipid binding activity of either protein, which can then tether to a microdomain on the late endosomal membrane. This process may help to bring the whole inflammasome complex along with pro‐IL‐1β to the late endosome, where they are enclosed into ILVs. In this sense, either IQGAP1 or GSDMD serves as an adaptor that bestows selectivity and stimulus‐dependency for exosomal IL‐1β release.

While our study begins to shed light on how inflammasome activation triggers selective packaging of exosomal contents for the secretion of IL‐1β as well as the whole inflammasome complex, the biological significance of this process requires more exploration. Exosomes are known to facilitate the expulsion of damaged molecules threatening to disrupt cellular homeostasis (Gurunathan *et al*, 2021). The resulting cleavage of GSDMD by activated inflammasomes is a terminal event leading to lytic cell death (Lieberman *et al*, 2019). Thus, the IQGAP1‐mediated inclusion of GSDMD and inflammasome into exosomes may be a mechanism for cells to survive inflammasome activation while producing inflammatory signals to activate necessary immune response.

As an interesting note, MLKL, the pore‐forming protein that drives necroptosis, was shown to regulate endosomal trafficking and extracellular vesicle biogenesis (Yoon *et al*, 2017), echoing the role of GSDMD, also a pore‐forming protein, in the production of exosome in response to inflammasome activation. This parallel implicates a general role for pore‐forming proteins in regulating intracellular trafficking of vesicles, which would contribute to a cytoprotective program by embedding potentially deleterious proteins with additional functions of containing and purging lethal biochemical cascades via exosomes.

Lastly, another question to be addressed by future study is how the enclosed IL‐1β may exit from exosomes once they are delivered into the extracellular space. Our understanding of exosomal cargo delivery in general is extremely limited, if at all. This remains an ongoing yet underexplored area of research. One tantalizing hypothesis is that extracellular inflammasome activity (Baroja‐Mazo *et al*, 2014; Franklin *et al*, 2014) along with other enzymes released during inflammatory response may facilitate the rupture of exosome membrane. Furthermore, it is also plausible that GSDMD will undergo a post‐release processing by activated caspase 8 and form pores in exosomes after being released to the extracellular space. Caspase 8 has been shown to exhibit proteolytic activity towards GSDMD at a low efficiency. This may have contributed to a lack of GSDMD processing inside the cells. However, a changing environment, especially an alteration in pH and oxidative stress in the extracellular space as is common in inflamed tissues, may facilitate and expedite GSDMD pore formation, leading to rupture of exosomes.

## Materials and Methods

### Animals

All animal experiments were conducted in accordance with IACUC guidelines at the Cleveland Clinic Lerner Research Institute. Adult (8‐week‐old to 16‐week‐old) male and female mice were used. All mice were maintained in a specific pathogen‐free facility under a strict 12‐h light/dark cycle and were fed the same standard autoclaved chow diet. *Iqgap1* ^−/−^ mice (Li *et al*, 2000) on 129S4/Sv background were revived from cryopreserved material purchased from the Jackson Laboratory (#025451). *Gsdmd* ^−/−^ mice on C57BL/6 background were kind gift from Dr. Vishva M. Dixit (Genentech, South San Francisco, CA, USA) and have been described previously (Kayagaki *et al*, 2015). 129S1/SvImJ (stock # 002448) and C57BL/6J (stock # 000664) purchased from Jackson Laboratory were used to generate heterozygous control mice. Experimental mice were produced by breeding heterozygous sire and dam to obtain littermate wild‐type and knockout mice. Wild‐type and knockout littermates of the same gender are co‐housed for experiments.

### Generation of knockout and knockdown cell lines

The immortalized mouse colonic epithelial cell line, YAMC, was a gift from Dr. Robert H. Whitehead at Vanderbilt University (Whitehead *et al*, 1993). To generate *IQGAP1* knockout YAMC cell line, two pairs of guide RNA sequences were used to knock out IQGAP1 (pair 1: 5′‐ TAAAGCACGTCTTGGTACGT‐3′; pair 2: 5′‐GAGTCTACCTTGCCAAGCTA‐3′). To generate GSDMD‐deficient HT29 cells, two pairs of gdRNA were used (pairs 1: 5′‐CACAAGCGTTCCACGAGCGA‐3′; pair 2: 5′‐ACGCGCACCCACAAGCGGGA‐3′). These guide RNA sequences were cloned into the pLenti‐Crispr v2 vector and transduced into target cells by lentiviral infection. To generate IQGAP1 knockdown HT29 cells, siRNAs purchased from Thermofisher (Cat: 4390824) were transfected into HT29 cells and 72 h after transfection the cells were used for experiments. To generate TSG101 knockdown cells, control and TSG101‐targeting shRNAs were delivered to YAMC cells by lentiviral infection. Two shRNA sequences were used to knockdown TSG101: 5′‐GCCTACTGTTTCTGCATCCTA‐3′ and 5′‐GCTATTGAAGACACTATCTTT‐3′. Infected cells were selected with puromycin for 1 week and then used for experiments. All knockout and knockdown cell lines were validated by Western blot to assess the ablation of targeted proteins.

### Plasmids

The mouse IQGAP1 Myc‐tagged plasmid was purchased from Origene #NM_016721. Mouse full length and truncation mutant *Iqgap1* and *Gsdmd* cDNAs were cloned into pLenti‐GFP vector for virus packaging and cell selection. Lentivirus was packaged using the restoration plasmids to infect YAMC cells. Seventy‐two hours after infection, GFP‐positive cells were sorted out as the restored cell. The protein expression of the restored cells was further validated by Western blot. Primers used for the generation of referenced constructs are listed in Table EV2.

### Histological analysis of DSS‐treated colon

Gender‐, age‐, background‐matched, and co‐housed mice were given 3% dextran sulfate sodium (DSS) (Colitis grade, MP Biomedicals #0216011025) in drinking water. In order to avoid extensive erosion of epithelium, mice were sacrificed and colons were collected after 3 days of DSS treatment. Excised colon was flushed with sterile PBS to remove fecal pellets. Next, the colons were opened longitudinally, fixed with 10% neutral buffered formalin overnight, and subjected to paraffin embedding for histological analysis.

### Colon explant culture

DSS‐treated colon tissues were rinsed with copious volume of cold sterile PBS and minced into 1 mm strips with sharp surgical blade. Minced colon strips were cultured in gentamycin‐supplemented DMEM medium overnight in the absence of serum. On the second day, cultured explants were treated with LPS for 4 h followed by 5 mM ATP for 30 min. The supernatant was collected for nanoparticle tracking analysis or exosome enrichment, followed by Western blotting.

### Exosome isolation and analysis

Exosomes were isolated as previously described (Bulek *et al*, 2020). Briefly, supernatant harvested from cell or colon explant culture was first centrifuged at 3,000 *g* for 10 min to remove the cell debris. The cleared supernatant was then subjected to size exclusion chromatography to enrich for extracellular vesicles using the qEV column (qEV, Izon Science, Christchurch, New Zealand) following the manufacturer's instructions. Fractions enriched with extracellular vesicles were then subjected to nanoparticle tracking using ZetaView or flow‐based assay to quantify the abundance of exosomes. Flow cytometry‐based semi‐quantification of exosome abundance was achieved with a combination of streptavidin‐decorated magnetic beads, biotinylated antimouse CD63, and a FITC‐conjugated hydrophobic dye for lipids. All reagents, except for antimouse CD63 (Biolegend #143918), were purchased as a kit from System Biosciences #EXOFLOW300A‐1. Exosomes from chromatography‐enriched fractions were captured and stained following manufacturers' instructions.

### Duolink proximity ligation assay

Duolink PLA assay (Sigma #DUO92014) was performed according to manufacturer's instruction. Briefly, cells were first seeded on cover slides 12 h prior to the treatment. After stimulation, cells were fixed with 4% paraformaldehyde (PFA) for 15 min at room temperature, and blocked at 37°C. After blocking, samples were incubated overnight with primary antibodies at 4°C. Next day, minus and plus probes were added to the sample and incubated at 37°C for 1 h, followed by ligation for 30 min at 37°C. Then the signal was amplified with polymerase at 37°C for 100 min. Samples were then counterstained with DAPI and signals were visualized under confocal microscope.

### Western blot and immunoprecipitation

Cells were lysed on ice using lysis buffer (0.5% Triton X‐100, 50 mM Tris–HCl (pH 7.4), 150 mM NaCl, 12.5 mM β‐glycerophosphate, 1.5 mM MgCl_2_, 10 mM NaF, 2 mM DTT, 2 mM sodium orthovanadate, 2 mM EGTA, and Protease Inhibitor Cocktail (Roche)) followed by centrifugation at 12,000 *g* for 20 min at 4°C. Supernatants were carefully collected for Western blot analysis or co‐immunoprecipitation. For immunoprecipitation, cell lysates or supernatants were incubated overnight at 4°C with targeting antibody and protein A/G Sepharose beads (GE Healthcare Life Sciences), followed by extensive washes with lysis buffer. Precipitates were eluted by SDS loading buffer and analyzed by the Western blot.

IL‐1β IP was performed as previously described (Duong *et al*, 2015). In brief, 5 ml of supernatants were incubated overnight at 4°C with 5 μg of biotinylated hamster monoclonal IgG1, clone B122 (Biolegend, San Diego, CA). On the next day, 25 μl of neutravidin agarose (Pierce Biotechnology, Waltham, MA) was added into each lysate. After 1 h incubation at 4°C, agarose resins were centrifuged, washed 3 times in lysis buffer, and heated in 30–40 μl 2× Laemmli buffer prior to Western blot analysis.

### Immunohistochemistry and immunofluorescence

Mouse tissue was fixed with 10% formalin overnight and kept in 70% ethanol at 4°C until processed into paraffin tissue blocks by the Imaging Core at Lerner Research Institute of Cleveland Clinic. Paraffin sections were subjected to heat‐induced epitope retrieval, as recommended by the antibody manufacturer before staining. De‐paraffinized, epitope‐retrieved sections were blocked with 10% normal goat serum (Life Technologies #50062Z) and then incubated with primary antibody overnight. Next day, the sections were washed with 0.05% Tween PBS followed by the incubation with corresponding secondary antibody and streptavidin‐HRP for immunohistochemistry or fluorescence‐conjugated secondary antibody for immunofluorescence staining. Staining was visualized with HRP‐substrate chromogen DAB (BD Pharmingen) or with confocal microscopy.

### Detection of IL‐1β biological activity

We adapted a reporter strategy employed by a widely used (Magupalli *et al*, 2020) commercial IL‐1β activity reporter cell line to measure IL‐1β biological activity. An NFκB luciferase reporter (Schindler & Baichwal, 1994) was transfected into a HEK293 cell line that stably overexpresses IL‐1R1. Six hours after transfection, concentrated exosome preparations were added to transfected cells, which were then incubated overnight. Stimulated cells were lysed, and cell lysates were subjected to luciferase activity assay using Promega Luciferase Activity Assay System (Cat # E1500). Exosomes used for bioactivity assay were prepared as described earlier with slight modifications. Enriched exosome preparations were subjected to further concentration using Amicon Ultra Centrifugal Filters, and the resultant concentrates were used to stimulate reporter cells.

### Antibodies

All the antibodies used in this study were listed in Table EV3.

### LC–MS/MS Method

IP and control IgG samples were fractionated on an SDS‐Page gel and entire lane cut into two bands, which were subjected to in‐gel digestion. Gel bands were divided into small pieces, washed with water, and dehydrated in acetonitrile. Bands were reduced with DTT and alkylated with iodoacetamide prior to in‐gel digestion, achieved by adding 5 μl of 10 ng/μl trypsin in 50 mM ammonium bicarbonate, and incubating overnight at room temperature. Formed peptides were extracted from polyacrylamide into two aliquots of 30 μl 50% acetonitrile with 5% formic acid. Extracts were combined and evaporated to < 10 μl by Speedvac and resuspended in 1% acetic acid to a final vol of ~ 30 μl for LC–MS analysis. Digested peptides were analyzed on a ThermoFisher Scientific UltiMate 3000 HPLC system (ThermoFisher Scientific, Bremen, Germany) interfaced with either a ThermoFisher Orbitrap Elite or ThermoFisher Scientific Orbitrap Fusion Lumos Tribrid mass spectrometer (Thermo Scientific, Bremen, Germany). Liquid chromatography was performed prior to MS/MS analysis for peptide separation. The HPLC column used was a Dionex 15 cm × 75 μm Acclaim Pepmap C18, 2 μm, 100 Å reversed‐phase capillary chromatography column. 5 μl vol of peptide extracts were injected and eluted from the column by a 90 min acetonitrile/0.1% formic acid gradient at a flow rate of 0.30 μl/min and introduced to the mass spectrometer source on line. Digests were analyzed using data‐dependent multitask capability of the instrument acquiring full scan mass spectra on a Fourier transform (FT) orbitrap analyzer to determine peptide MWs and collision‐induced dissociation (CID) MS/MS product ion spectra with an ion‐trap analyzer to determine the aa sequence in successive instrument scans. In order to do label‐free quantitative and qualitative proteomic analysis, data were searched using X! Tandem (The GPM) and Sequest (bundled into Proteome Discoverer 2.2, Thermo Fisher Scientific, San Jose, CA). The database used in these searches corresponds to the mouse SwissProtKB database. The parameters used for these searches include enzyme specific trypsin with a maximum of two missed cleavages, carbamidomethyl (C) as a fixed modification, oxidation of methionine and protein N‐terminal acetylation as variable modifications, peptide mass tolerance of 10 ppm and fragment ion mass tolerance of 0.6 Da. Scaffold (version Scaffold 4.8.9, Proteome Software Inc., Portland, OR) was used to validate MS/MS‐based peptide and protein identifications. Peptide identifications were accepted if they could be established at > 0.0% probability by the Peptide Prophet algorithm (Keller *et al*, 2002) with Scaffold delta‐mass correction. Protein identifications were accepted if they could be established at > 99.9% probability and contained at least two identified peptides. Protein probabilities were assigned by the Protein Prophet algorithm (Nesvizhskii *et al*, 2003). Proteins that contained similar peptides and could not be differentiated based on MS/MS analysis alone were grouped to satisfy the principles of parsimony. Label‐free spectral counting was used to determine relative differences in the IgG control and IP samples.

### Statistics and reproducibility

Statistical significance was determined using the *Student*'s *t*‐test when comparing the mean of two groups. Differences shown in the figures were presented as the mean ± SEM. All tests were two sided, and *P* value < 0.05 was considered to be statistically significant. All the statistics were performed using Graphpad Prism 8.0. All the image quantifications were done by the Image‐Pro (version 7.0). All Western blots were repeated independently at least three times with similar results and the representative results were shown. Unless specified otherwise, all imaging experiments were repeated independently for at least 3 times, and quantification was done based on 5 high magnification field views.

## Author contributions

**Yun Liao:** Data curation; formal analysis; validation; investigation; methodology. **Xing Chen:** Formal analysis; investigation; methodology. **William Miller‐Little:** Investigation. **Han Wang:** Data curation; formal analysis; methodology. **Belinda Willard:** Methodology. **Katarzyna Bulek:** Conceptualization; resources; formal analysis; supervision; validation; investigation; methodology; writing – original draft; project administration; writing—review and editing. **Junjie Zhao:** Conceptualization; formal analysis; validation; investigation; writing—original draft; writing—review and editing. **Xiaoxia Li:** Conceptualization; supervision; funding acquisition; investigation; writing—original draft; project administration; writing—review and editing.

## Disclosure and competing interests statement

The authors declare that they have no conflict of interest.

## Supporting information



Expanded View Figures PDF

Table EV1

Table EV2

Table EV3

Source Data for Expanded View

PDF+

Source Data for Figure 1

Source Data for Figure 2

Source Data for Figure 3

Source Data for Figure 4

Source Data for Figure 5

Source Data for Figure 6

## Data Availability

This study includes no data that would need to be deposited in external repositories.
