# Supplementary material for: The Ras GTPase‐activating‐like protein IQGAP1 bridges Gasdermin D to the ESCRT system to promote IL‐1β release via exosomes
Source: EMBO J. 2022 Nov 14;42(1):e110780. doi: 10.15252/embj.2022110780 (PMC9811620; doi:10.15252/embj.2022110780)
Supplement: Supplementary file 1 — Expanded View Figures PDF [file EMBJ-42-e110780-s012.pdf]

Expanded View Figures

Figure EV1. LPS and ATP-induces nonpyroptotic NLRP3 inflammasome activation.

A Exosomal proteins concentrated from 10^7 exosomes were prepared from supernatants of YAMCs that were left untreated or treated with LPS (4 h) and ATP (30 min). HEK293 cells stably expressing IL-1R1 were transfected with an NFκB luciferase reporter construct and stimulated with indicated exosome preparations in the presence or absence of anti-IL-1β overnight. Cell lysates were analyzed for luciferase activity. Data were presented as mean ± SEM.

B Western blot analysis of supernatants and whole-cell lysates collected from YAMC cells with and without LPS stimulation for 4 h or LPS plus ATP (4 h plus 30 min) in the presence or absence of MCC950 (100 nM).

C Western blot analysis of supernatants and whole-cell lysates collected from YAMC cells with and without LPS stimulation for 4 h or LPS plus ATP (4 h plus 30 min) in the presence or absence of Z-IETD-FMK (10 μM) or Ac-YVAD-cmk (10 μM).

D Western blot analysis of supernatants and whole-cell lysates collected from GSDMD KO YAMC cells restored with GSDMD full length (FL) or I105N mutant. Cells were stimulated with or without LPS stimulation for 4 h or LPS plus ATP (4 h plus 30 min) as indicated.

E GSDMD KO YAMC cells expressing either full length GSDMD or I105N mutant were stimulated with or without LPS for 4 h or LPS plus ATP (4 h plus 30 min) in phenol red-free DMEM supplemented with 1 μg/ml PI. As controls, the cells were treated with 0.05% saponin for 1 min in the presence of 1 μg/ml PI. Phase-contrast picture were taken. Scale bar, 100 μm.

F Exosome preparations from colon explants of DSS-treated *wild-type* (WT) and *Il1b*$^{-/-}$ mice were subjected to IL-1β bioactivity assay. Exosomal proteins concentrated from 10^7 exosomes were prepared from supernatants. HEK293 cells stably expressing IL-1R1 were transfected with an NFκB luciferase reporter construct and stimulated with indicated exosome preparations in the presence or absence of anti-IL-1β overnight. Cell lysates were analyzed for luciferase activity. $n = 3$ mice. Experiments were repeated twice.

Data information: For panels A and F, data were presented as mean ± SEM. ***$P < 0.001$, ****$P < 0.0001$ by unpaired two-tailed t-test. Unless specified, all experiments were repeated three times with consistent results. The representative results are shown.
Source data are available online for this figure.

 © 2022 The Authors

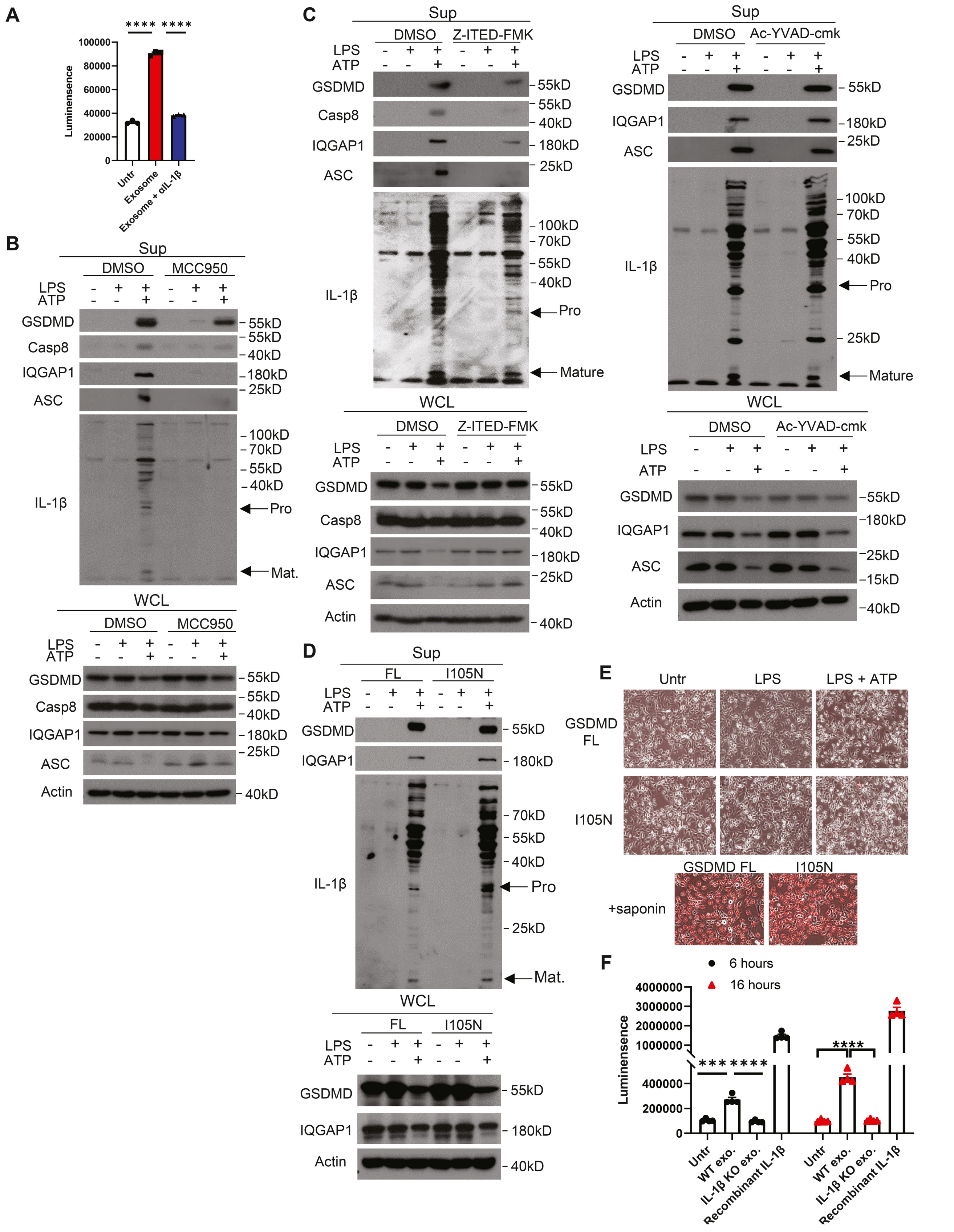
A
B
C
D
E
F
Luminensence
Untr
Exosome
Exosome + αIL-1β
Sup
WCL
DMSO
MCC950
Z-ITED-FMK
Ac-YVAD-cmk
FL
I105N
LPS
ATP
GSDMD
Casp8
IQGAP1
ASC
IL-1β
Actin
Pro
Mature
Mat.
Untr
LPS
LPS + ATP
GSDMD FL
I105N
+saponin
6 hours
16 hours
WT exo.
IL-1β KO exo.
Recombinant IL-1β

Figure EV1.

Figure EV2. GSDMD interacts with IQGAP1.

A Endogenous GSDMD were immunoprecipitated from lysates of wild-type (WT) and IQGAP1-deficient (IQGAP1 KO) YAMC cells that had been treated as indicated. The precipitates were analyzed by Western blot.

B Proximity ligation assay (PLA) for IQGAP1-GSDMD interaction in WT and GSDMD-deficient (GSDMD KO) YAMC cells after LPS plus ATP treatment. Scale bar, 10 μm.

C HT-29 cells were transfected with either scramble or IQGAP1-targeting siRNA followed by stimulation with LPS for 4 h 48 h post transfection. After LPS stimulation, cells were further exposed to ATP for 30 min. Left panel: After ATP stimulation cells were subjected to PLA for endogenous IQGAP1 and GSDMD. Following PLA, cells were counter stained with DAPI and visualized under confocal microscope. Scale bar: 10 μm. Right panel: Lysates from treated and untreated cells were subjected to coimmunoprecipitation for GSDMD and probed for indicated proteins.

D Supernatants from cells treated as described in panel (A) were subjected to methanol treatment to precipitate total protein, which were then solubilized with Laemmli buffer and analyzed by Western blot.

Data information: All experiments were repeated 3 times and yielded consistent results. The representative results are shown.
Source data are available online for this figure.

 © 2022 The Authors

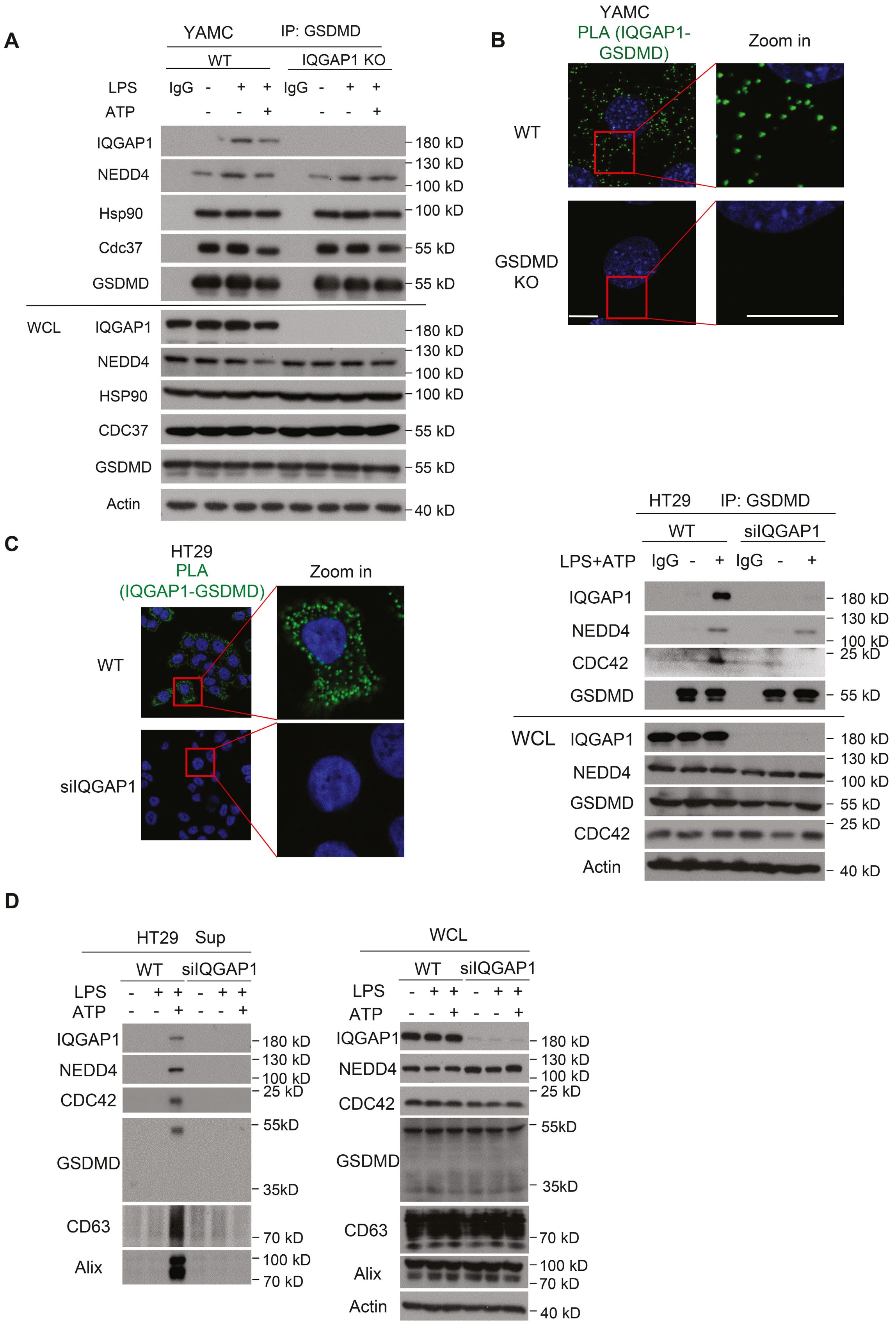
A
YAMC IP: GSDMD
WT
IQGAP1 KO
LPS
ATP
IgG
IQGAP1
NEDD4
Hsp90
Cdc37
GSDMD
WCL
HSP90
CDC37
Actin
180 kD
130 kD
100 kD
55 kD
40 kD
B
YAMC
PLA (IQGAP1-GSDMD)
Zoom in
WT
GSDMD KO
C
HT29
PLA (IQGAP1-GSDMD)
siIQGAP1
HT29 IP: GSDMD
LPS+ATP
CDC42
25 kD
D
HT29 Sup
CD63
Alix
55kD
35kD
70 kD

Figure EV2.

Figure EV3. ESCRT is required for exosomal pro-IL-1β release.

A YAMCs were transfected with either scramble or Vps24-targeting siRNA followed by stimulation with LPS for 4 h 48 h post transfection. After LPS stimulation, cells were further exposed to ATP for 30 min. Supernatants from cells treated described in panel (A) were subjected to methanol treatment to precipitate total protein, which were then solubilized with Laemmli buffer and analyzed by Western blot.

B Nanoparticle tracking analysis of supernatant of scramble siRNA or Vps24 targeting siRNA-transfected YAMC cells treated with LPS plus ATP. Data were presented as mean ± SEM $*P < 0.05$ by unpaired two-tailed *t*-test.

C WT and IQGAP1 KO YAMC cells were left unstimulated and treated with 0.05% saponin for 1 min in phenol red-free DMEM supplemented with 1 μg/ml PI. Five field views were counted. Data were shown as mean ± SEM. $*P < 0.05$ by unpaired two-tailed *t*-test. Scale bar: 100 μm.

D Western blot analysis of supernatants and whole-cell lysates collected from YAMC cells treated with 0.05% saponin (for 1 min) or LPS (4 h) plus ATP (30 min).

E Western blot analysis of supernatants and whole-cell lysates collected from YAMC cells with and without LPS stimulation for 4 h or LPS plus ATP (4 h plus 30 min) in the presence or absence of BAPTA-AM (6 μM).

Data information: All experiments were repeated 3 times and yielded consistent results. The representative results are shown.
Source data are available online for this figure.

 © 2022 The Authors

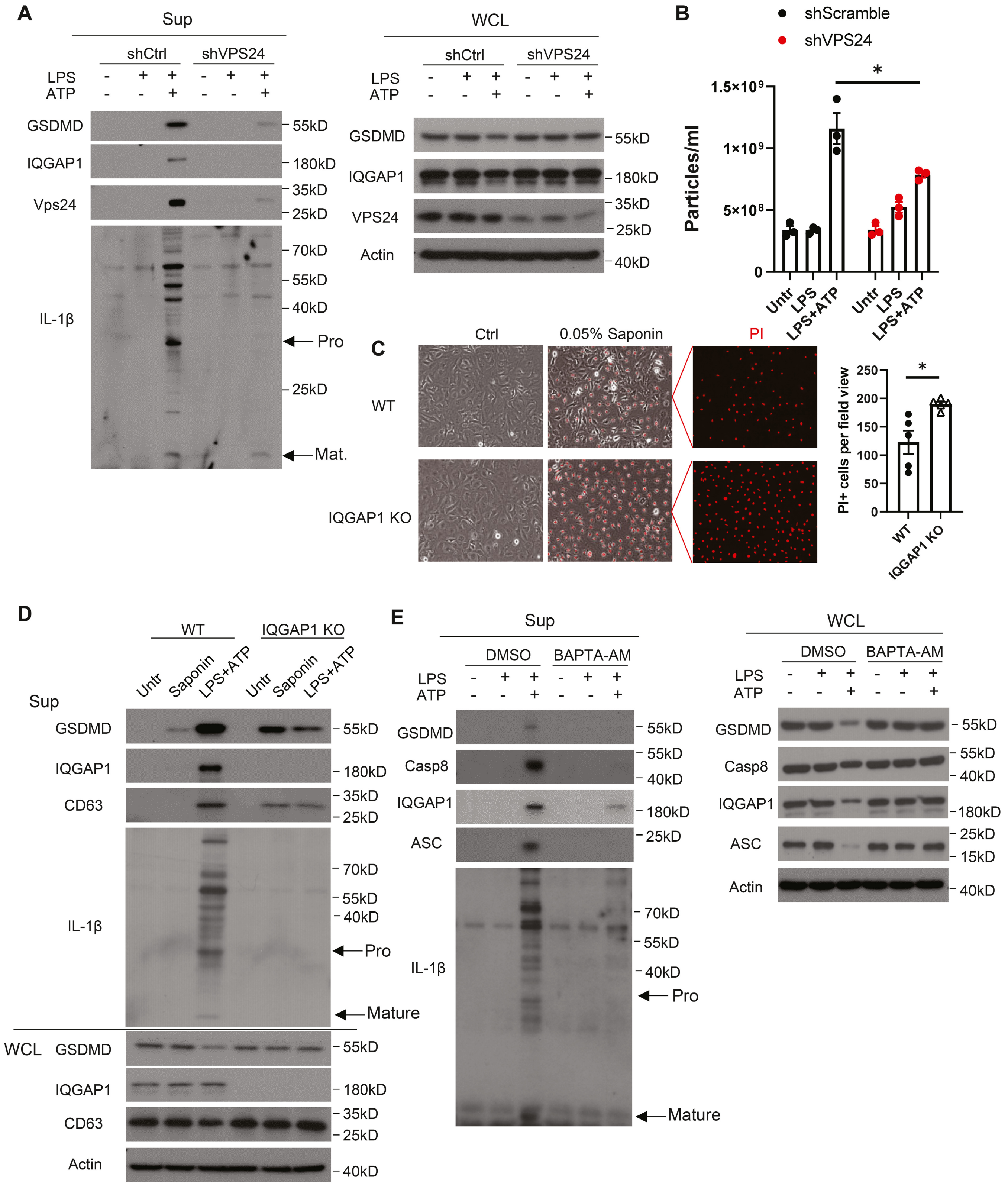
A
Sup
shCtrl
shVPS24
LPS
ATP
GSDMD
IQGAP1
Vps24
IL-1β
55kD
180kD
35kD
25kD
70kD
55kD
40kD
Pro
25kD
Mat.
WCL
shCtrl
shVPS24
LPS
ATP
GSDMD
IQGAP1
VPS24
Actin
55kD
180kD
35kD
25kD
40kD
B
shScramble
shVPS24
Particles/ml
1.5×10⁹
1×10⁹
5×10⁸
0
Untr
LPS
LPS+ATP
Untr
LPS
LPS+ATP
C
Ctrl
0.05% Saponin
PI
WT
IQGAP1 KO
PI+ cells per field view
250
200
150
100
50
0
WT
IQGAP1 KO
D
WT
IQGAP1 KO
Untr
Saponin
LPS+ATP
Untr
Saponin
LPS+ATP
Sup
GSDMD
IQGAP1
CD63
IL-1β
55kD
180kD
35kD
25kD
70kD
55kD
40kD
Pro
Mature
WCL
GSDMD
IQGAP1
CD63
Actin
55kD
180kD
35kD
25kD
40kD
E
Sup
DMSO
BAPTA-AM
LPS
ATP
GSDMD
Casp8
IQGAP1
ASC
IL-1β
55kD
55kD
40kD
180kD
25kD
70kD
55kD
40kD
Pro
Mature
WCL
DMSO
BAPTA-AM
LPS
ATP
GSDMD
Casp8
IQGAP1
ASC
Actin
55kD
55kD
40kD
180kD
25kD
15kD
40kD

Figure EV3.
