# Supplementary material for: The Ras GTPase‐activating‐like protein IQGAP1 bridges Gasdermin D to the ESCRT system to promote IL‐1β release via exosomes
Source: EMBO J. 2022 Nov 14;42(1):e110780. doi: 10.15252/embj.2022110780 (PMC9811620; doi:10.15252/embj.2022110780)
Supplement: Supplementary file 2 — Table EV1 [file EMBJ-42-e110780-s006.docx]

| **[M+H]** | **m/z** | **z** | **Δm(ppm)** | **Residues** | **Sequence** |
| --- | --- | --- | --- | --- | --- |
|  |  |  |  |  |  |
| 2,610.27 | 871.0964 | 3 | -0.01547 | 2-25 | {M}ace-SAAEEVDGLGVVRPHYGSVLDNER(L) |
| 908.4721 | 455.2434 | 2 | -3.872 | 81-88 | (K)LGNFFSPK(V) |
| 3,113.68 | 779.4285 | 4 | 0.3802 | 202-231 | (K)IGGILANELSVDEAALHAAVIAINEAIDRR(V) |
| 3,116.47 | 1,039.83 | 3 | -0.7275 | 359-387 | (R)QSGQTDPLQKEEVQAGVDAANSAAQQYQR(R) |
| 2,893.45 | 965.4922 | 3 | -0.9477 | 407-431 | (K)TVLELMoNPEAQLPQVYPFAADLYQK(E) |
| 1,882.00 | 942.009 | 2 | -0.943 | 539-556 | (R)ILAIGLINEALDEGDAQK(T) |
| 2,054.08 | 685.701 | 3 | 0.5824 | 568-585 | (K)LEGVLAEVAQHYQDTLIR(A) |
| 1,723.84 | 862.925 | 2 | -1.24 | 723-738 | (R)EEIQSSISGVTAAYNR(E) |
| 1,789.85 | 895.9333 | 2 | 0.07988 | 902-916 | (R)SNQQLENDLNLMoDIK(I) |
| 3,000.47 | 1,001.16 | 3 | -0.6507 | 1001-1024 | (K)FMoDSVIFTLYNYASNQREEYLLLR(L) |
| 1,318.71 | 660.3625 | 2 | -2.305 | 1025-1035 | (R)LFQTALQEEIK(S) |
| 2,586.24 | 863.0889 | 3 | 0.8596 | 1240-1261 | (K)MoFLGDNAHLSIINEYLSQSYQK(F) |
| 2,867.36 | 956.7938 | 3 | 0.658 | 1398-1422 | (R)FQPGETLTEILETPATNEQEAEHQR(A) |
| 1,482.68 | 742.3462 | 2 | 7.478 | 1517-1528 | (K)ATFYGEQVDYYK(S) |

**Table EV1.** IQGAP1 peptides detected by mass spectrometric analysis of GSDMD co-precipitates
