# Supplementary material for: The Ras GTPase‐activating‐like protein IQGAP1 bridges Gasdermin D to the ESCRT system to promote IL‐1β release via exosomes
Source: EMBO J. 2022 Nov 14;42(1):e110780. doi: 10.15252/embj.2022110780 (PMC9811620; doi:10.15252/embj.2022110780)
Supplement: Supplementary file 3 — Table EV2 [file EMBJ-42-e110780-s004.docx]

| **Table EV2. Primers for constructing referenced plasmids** | |
| --- | --- |
| **GSDMD-HA** |  |
| Forward | 5'-ACGCATAAGCTTGCCACCATGCCATCGGCCTTTGAGAAAG-3' |
| Reverse | 5'-CGATATGGATCCCTAAGCGTAATCTGGAACATCGTATGGGTAACAAGGTTTCTGGCCTAGACTTG-3' |
|  |  |
| **GSDMD-flag** |  |
| Forward | 5'-ACGCATAAGCTTGCCACCATGCCATCGGCCTTTGAGAAAG-3' |
| Reverse | 5'-CGATATGGATCCCTACTTGTCGTCATCGTCTTTGTAGTCACAAGGTTTCTGGCCTAGACTTG-3' |
|  |  |
| **GSDMD N-terminal-HA** | |
| Forward | 5'-ACGCATAAGCTTGCCACCATGCCATCGGCCTTTGAGAAAG |
| Reverse | 5'-CGATATGGATCCCTAAGCGTAATCTGGAACATCGTATGGGTAATCTGACAGGAGACTGAGCTGC-3' |
|  |  |
| **GSDMD C-terminal-HA** |  |
| Forward | 5'-ACGCATAAGCTTGCCACCATGGGGATTGATGAGGAGGAATTA-3' |
| Reverse | 5'-CGATATGGATCCCTAAGCGTAATCTGGAACATCGTATGGGTAACAAGGTTTCTGGCCTAGACTTG-3' |
|  |  |
| **GSDMD I105N** |  |
| Forward | 5'-GGGAGAAGGGAAAAATTCTGGTGGGGCTGC-3' |
| Reverse | 5'-GCAGCCCCACCAGAATTTTTCCCTTCTCCC-3' |
|  |  |
| **GSDMD D276A** |  |
| Forward | 5'-GTCTCCTGTCAGCTGGGATTGATGAGGAGG-3' |
| Reverse | 5'-CCTCCTCATCAATCCCAGCTGACAGGAGAC-3' |
|  |  |
| **IQGAP1 truncations** |  |
| **ΔCHD** |  |
| Forward | 5'-GCAGAACGTGGCTTATGAATACCTTTGTAAACTGGGCCTGGCTCCTCAGATTC-3' |
| Reverse | 5'-GAATCTGAGGAGCCAGGCCCAGTTTACAAAGGTATTCATAAGCCACGTTCTGC-3' |
|  |  |
| **ΔWW** |  |
| Forward | 5'-GAAGAAGAGACTGGCAGCAGGAGATGGCTTGATCACCAAGCTGCAAGCCTGC-3' |
| Reverse | 5'-GCAGGCTTGCAGCTTGGTGATCAAGCCATCTCCTGCTGCCAGTCTCTTCTTC-3' |
|  |  |
| **ΔIQ** |  |
| Forward | 5'-CCGAGAGCAGCTTTGGCTGGCCAACACTCTCATCAATGCTGAGGACCCGC-3' |
| Reverse | 5'-GCGGGTCCTCAGCATTGATGAGAGTGTTGGCCAGCCAAAGCTGCTCTCGG-3' |
|  |  |
| **ΔGRD** |  |
| Forward | 5'-CTAACCAGCGGGAGGAGTACCTGCTGCTGCGGAAGATGTTTCTGGGCGATAATGCCCACTTAAGC-3' |
| Reverse | 5'-GCTTAAGTGGGCATTATCGCCCAGAAACATCTTCCGCAGCAGCAGGTACTCCTCCCGCTGGTTAG-3' |
|  |  |
|  |  |
| **IQGAP1 pLenti-GFP restored primer** (ΔIQ, ΔGRD restoration used the same primer but different templates as generated above) | |
| Forward | 5'-GaagattctagagctagcgaattATGTCCGCCGCGGAGGAGGTTG-3' |
| Reverse | 5'-cagatccttgcggccgcggatcctaCTTGTCGTCATCGTCTTTGTAGTC TTAGTGATAAAAATAGAGTTCT-3' |
