# Supplementary material for: The Ras GTPase‐activating‐like protein IQGAP1 bridges Gasdermin D to the ESCRT system to promote IL‐1β release via exosomes
Source: EMBO J. 2022 Nov 14;42(1):e110780. doi: 10.15252/embj.2022110780 (PMC9811620; doi:10.15252/embj.2022110780)
Supplement: Supplementary file 4 — Table EV3 [file EMBJ-42-e110780-s001.docx]

| **Table EV3. Antibody used in this study** | | | | |
| --- | --- | --- | --- | --- |
| **Protein** | **Clone No.** | **Catlog No.** | **Company** | **Usage** |
| GSDMD | EPR19828 | ab209845 | Abcam | 1:1000 for WB/1:100 for staining |
| GSDMD | EPR20859 | ab219800 | Abcam | 1:1000 for WB/1:100 for staining |
| GSDMD | N.A. | A17308 | Abclonal | 1:1000 for WB detecting GSDMD FL and C terminal |
| GSDMD | 3F12-1B2 | H00079792-M01 | Abnova | 1:1000 for WB detecting human GSDMD |
| Caspase-8 | D35G2 | 4790S | Cell signaling | 1:1000 for WB |
| IQGAP1 | C-9 | sc-376021 | Santa Cruz | 1:1000 for WB/1:50 for staining/1:100 for immunprecipitation |
| IQGAP1 | D8K4X | 20648 | Cell signaling | 1:100 for staining |
| HSP90 | F-8 | sc-13119 | Santa Cruz | 1:1000 for WB |
| CDC37 | D11A3 | 4793 | Cell signaling | 1:1000 for WB |
| NEDD4 | N.A. | 2740 | Cell signaling | 1:1000 for WB |
| IL-1β | B122 | 503505 | Biolegend | 1:100 for immunoprecipitation |
| IL-1β | N.A. | AF-401 | R&D | 1:1000 for WB |
| Actin | 8H10D10 | 3700s | Cell signaling | 1:10000 for WB |
| CD63 | EPR21151 | ab217345 | Abcam | 1:1000 for WB |
| CD63 | NVG-2 | 143902 | Biolegend | 1:100 for staining |
| CD63 | NVG-2 | 143904 | Biolegend | 1:100 for staining |
| Alix | 3A9 | ab117600 | Abcam | 1:1000 for WB |
| Flag | M2 | F3165 | Sigma | 1:1000 for WB/1:1000 for immunoprecipitation |
| DYKDDDDK | D6W5B | 14793s | Cell signaling | 1:1000 for WB/1:1000 for immunoprecipitation/1:100 for staining |
| Myc-tag | 9B11 | 2276s | Cell signaling | 1:1000 for WB/1:1000 for immunoprecipitation |
| HA | HA-7 | H9658 | Sigma | 1:1000 for WB/1:1000 for immunoprecipitation |
| EEA1 | C45B10 | 3288s | Cell signaling | 1:100 for staining |
| TSG101 | N.A. | HPA006161 | Sigma | 1:1000 for WB/1:100 for staining |
| TSG101 | C-2 | sc-7964 | Santa Cruz | 1:100 for immunoprecipitation |
| CDC42 | N.A. | ab155940 | Abcam | 1:1000 for WB |
| CDC42-GTP | N.A. | 26905 | NewEast Biosciences | 1:100 for immunoprecipitation and 1:50 for staining |
