# Supplementary figures and images for: The Ras GTPase‐activating‐like protein IQGAP1 bridges Gasdermin D to the ESCRT system to promote IL‐1β release via exosomes (part 1 of 3)
Source: EMBO J. 2022 Nov 14;42(1):e110780. doi: 10.15252/embj.2022110780 (PMC9811620; doi:10.15252/embj.2022110780)

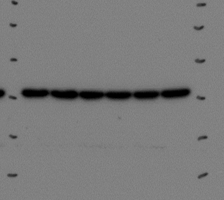

Supplement: Supplementary file 5 — Source Data for Expanded View [file EMBJ-42-e110780-s003.zip › Figure EV1/B/Actin_WCL.tif]

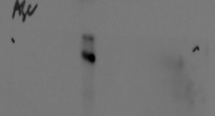

Supplement: Supplementary file 5 — Source Data for Expanded View [file EMBJ-42-e110780-s003.zip › Figure EV1/B/ASC.tif]

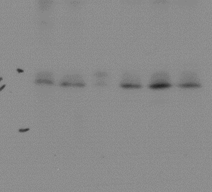

Supplement: Supplementary file 5 — Source Data for Expanded View [file EMBJ-42-e110780-s003.zip › Figure EV1/B/ASC_WCL.tif]

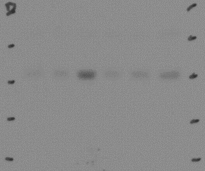

Supplement: Supplementary file 5 — Source Data for Expanded View [file EMBJ-42-e110780-s003.zip › Figure EV1/B/Casp8.tif]

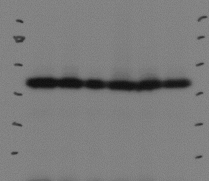

Supplement: Supplementary file 5 — Source Data for Expanded View [file EMBJ-42-e110780-s003.zip › Figure EV1/B/Casp8_WCL.tif]

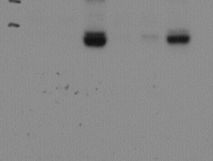

Supplement: Supplementary file 5 — Source Data for Expanded View [file EMBJ-42-e110780-s003.zip › Figure EV1/B/GSDMD.tif]

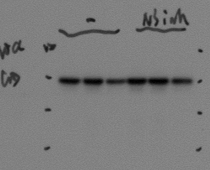

Supplement: Supplementary file 5 — Source Data for Expanded View [file EMBJ-42-e110780-s003.zip › Figure EV1/B/GSDMD_WCL.tif]

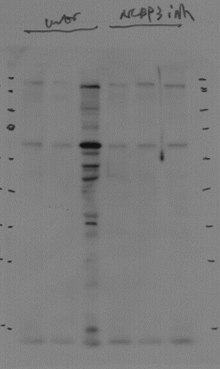

Supplement: Supplementary file 5 — Source Data for Expanded View [file EMBJ-42-e110780-s003.zip › Figure EV1/B/IL-1b.tif]

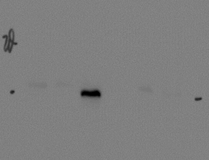

Supplement: Supplementary file 5 — Source Data for Expanded View [file EMBJ-42-e110780-s003.zip › Figure EV1/B/IQGAP1.tif]

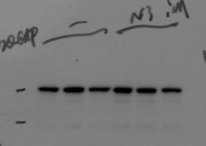

Supplement: Supplementary file 5 — Source Data for Expanded View [file EMBJ-42-e110780-s003.zip › Figure EV1/B/IQGAP1_WCL.tif]

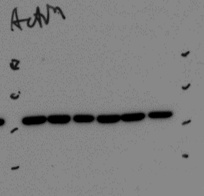

Supplement: Supplementary file 5 — Source Data for Expanded View [file EMBJ-42-e110780-s003.zip › Figure EV1/C/C_Casp1inh/Actin_WCL.tif]

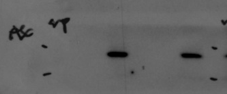

Supplement: Supplementary file 5 — Source Data for Expanded View [file EMBJ-42-e110780-s003.zip › Figure EV1/C/C_Casp1inh/ASC.tif]

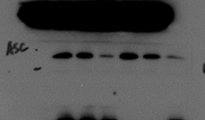

Supplement: Supplementary file 5 — Source Data for Expanded View [file EMBJ-42-e110780-s003.zip › Figure EV1/C/C_Casp1inh/ASC_WCL.tif]

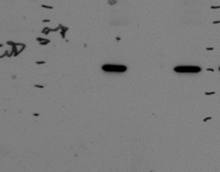

Supplement: Supplementary file 5 — Source Data for Expanded View [file EMBJ-42-e110780-s003.zip › Figure EV1/C/C_Casp1inh/GSDMD.tif]

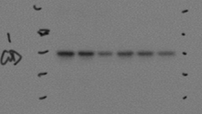

Supplement: Supplementary file 5 — Source Data for Expanded View [file EMBJ-42-e110780-s003.zip › Figure EV1/C/C_Casp1inh/GSDMD_WCL.tif]

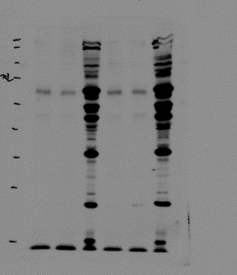

Supplement: Supplementary file 5 — Source Data for Expanded View [file EMBJ-42-e110780-s003.zip › Figure EV1/C/C_Casp1inh/IL-1b.tif]

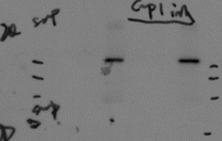

Supplement: Supplementary file 5 — Source Data for Expanded View [file EMBJ-42-e110780-s003.zip › Figure EV1/C/C_Casp1inh/IQGAP1.tif]

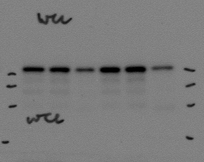

Supplement: Supplementary file 5 — Source Data for Expanded View [file EMBJ-42-e110780-s003.zip › Figure EV1/C/C_Casp1inh/IQGAP1_WCL.tif]

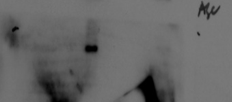

Supplement: Supplementary file 5 — Source Data for Expanded View [file EMBJ-42-e110780-s003.zip › Figure EV1/C/C_Casp8inh/ACS.tif]

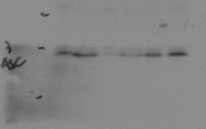

Supplement: Supplementary file 5 — Source Data for Expanded View [file EMBJ-42-e110780-s003.zip › Figure EV1/C/C_Casp8inh/ACS_WCL.tif]

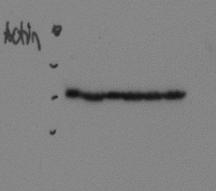

Supplement: Supplementary file 5 — Source Data for Expanded View [file EMBJ-42-e110780-s003.zip › Figure EV1/C/C_Casp8inh/Actin_WCL.tif]

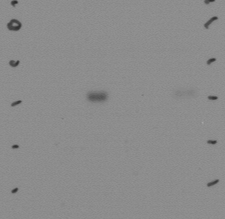

Supplement: Supplementary file 5 — Source Data for Expanded View [file EMBJ-42-e110780-s003.zip › Figure EV1/C/C_Casp8inh/Casp8.tif]

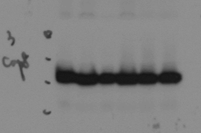

Supplement: Supplementary file 5 — Source Data for Expanded View [file EMBJ-42-e110780-s003.zip › Figure EV1/C/C_Casp8inh/Casp8_WCL.tif]

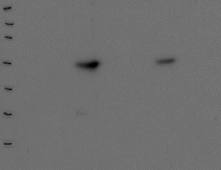

Supplement: Supplementary file 5 — Source Data for Expanded View [file EMBJ-42-e110780-s003.zip › Figure EV1/C/C_Casp8inh/GSDMD.tif]

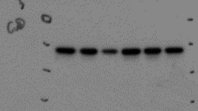

Supplement: Supplementary file 5 — Source Data for Expanded View [file EMBJ-42-e110780-s003.zip › Figure EV1/C/C_Casp8inh/GSDMD_WCL.tif]

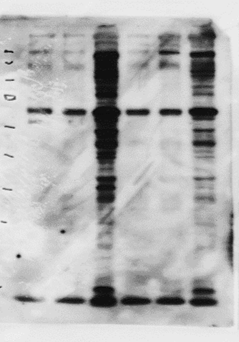

Supplement: Supplementary file 5 — Source Data for Expanded View [file EMBJ-42-e110780-s003.zip › Figure EV1/C/C_Casp8inh/IL-1b.tif]

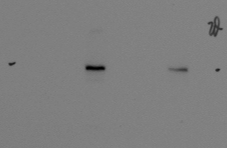

Supplement: Supplementary file 5 — Source Data for Expanded View [file EMBJ-42-e110780-s003.zip › Figure EV1/C/C_Casp8inh/IQGAP1.tif]

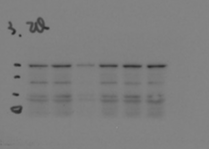

Supplement: Supplementary file 5 — Source Data for Expanded View [file EMBJ-42-e110780-s003.zip › Figure EV1/C/C_Casp8inh/IQGAP1_WCL.tif]

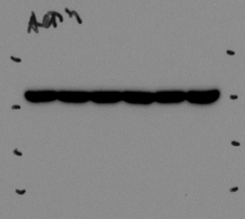

Supplement: Supplementary file 5 — Source Data for Expanded View [file EMBJ-42-e110780-s003.zip › Figure EV1/D/Actin_WCL.tif]

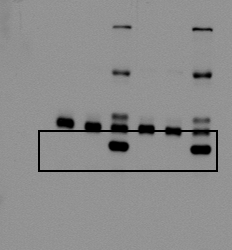

Supplement: Supplementary file 5 — Source Data for Expanded View [file EMBJ-42-e110780-s003.zip › Figure EV1/D/GSDMD.tif]

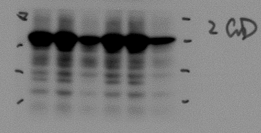

Supplement: Supplementary file 5 — Source Data for Expanded View [file EMBJ-42-e110780-s003.zip › Figure EV1/D/GSDMD_WCL.tif]

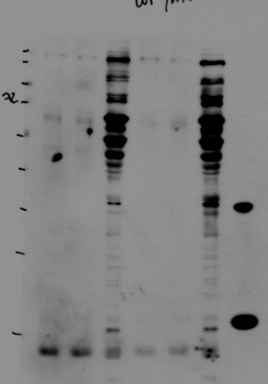

Supplement: Supplementary file 5 — Source Data for Expanded View [file EMBJ-42-e110780-s003.zip › Figure EV1/D/IL-1b.tif]

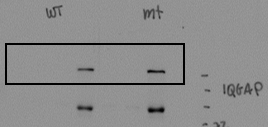

Supplement: Supplementary file 5 — Source Data for Expanded View [file EMBJ-42-e110780-s003.zip › Figure EV1/D/IQGAP1.tif]

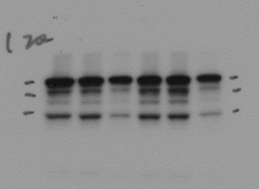

Supplement: Supplementary file 5 — Source Data for Expanded View [file EMBJ-42-e110780-s003.zip › Figure EV1/D/IQGAP1_WCL.tif]

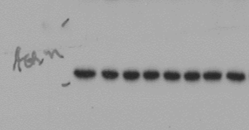

Supplement: Supplementary file 5 — Source Data for Expanded View [file EMBJ-42-e110780-s003.zip › Figure EV2/A/Actin_WCL.tif]

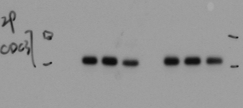

Supplement: Supplementary file 5 — Source Data for Expanded View [file EMBJ-42-e110780-s003.zip › Figure EV2/A/CDC37.tif]

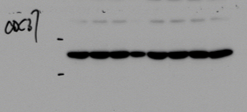

Supplement: Supplementary file 5 — Source Data for Expanded View [file EMBJ-42-e110780-s003.zip › Figure EV2/A/CDC37_WCL.tif]

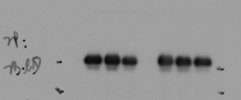

Supplement: Supplementary file 5 — Source Data for Expanded View [file EMBJ-42-e110780-s003.zip › Figure EV2/A/GSDMD.tif]

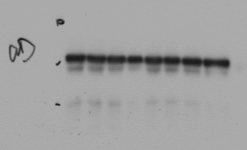

Supplement: Supplementary file 5 — Source Data for Expanded View [file EMBJ-42-e110780-s003.zip › Figure EV2/A/GSDMD_WCL.tif]

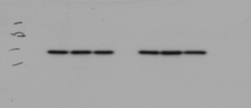

Supplement: Supplementary file 5 — Source Data for Expanded View [file EMBJ-42-e110780-s003.zip › Figure EV2/A/Hsp90.tif]

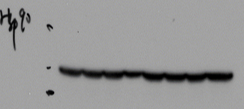

Supplement: Supplementary file 5 — Source Data for Expanded View [file EMBJ-42-e110780-s003.zip › Figure EV2/A/Hsp90_WCL.tif]

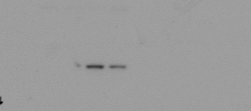

Supplement: Supplementary file 5 — Source Data for Expanded View [file EMBJ-42-e110780-s003.zip › Figure EV2/A/IQGAP1.tif]

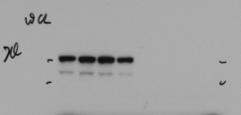

Supplement: Supplementary file 5 — Source Data for Expanded View [file EMBJ-42-e110780-s003.zip › Figure EV2/A/IQGAP1_WCL.tif]

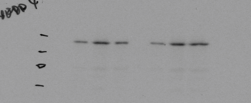

Supplement: Supplementary file 5 — Source Data for Expanded View [file EMBJ-42-e110780-s003.zip › Figure EV2/A/NEDD4.tif]

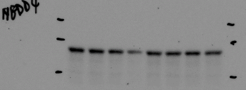

Supplement: Supplementary file 5 — Source Data for Expanded View [file EMBJ-42-e110780-s003.zip › Figure EV2/A/NEDD4_WCL.tif]

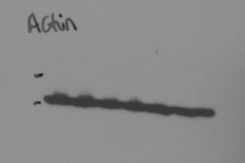

Supplement: Supplementary file 5 — Source Data for Expanded View [file EMBJ-42-e110780-s003.zip › Figure EV2/C/Actin_WCL.tif]

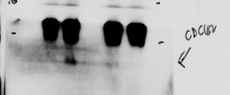

Supplement: Supplementary file 5 — Source Data for Expanded View [file EMBJ-42-e110780-s003.zip › Figure EV2/C/CDC42.tif]

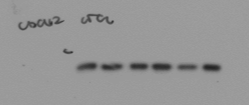

Supplement: Supplementary file 5 — Source Data for Expanded View [file EMBJ-42-e110780-s003.zip › Figure EV2/C/CDC42_WCL.tif]

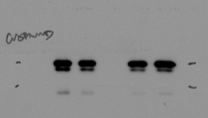

Supplement: Supplementary file 5 — Source Data for Expanded View [file EMBJ-42-e110780-s003.zip › Figure EV2/C/GSDMD.tif]

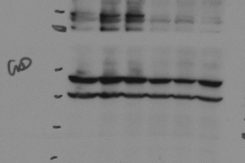

Supplement: Supplementary file 5 — Source Data for Expanded View [file EMBJ-42-e110780-s003.zip › Figure EV2/C/GSDMD_WCL.tif]

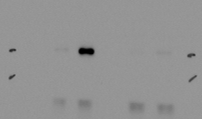

Supplement: Supplementary file 5 — Source Data for Expanded View [file EMBJ-42-e110780-s003.zip › Figure EV2/C/IQGAP1.tif]

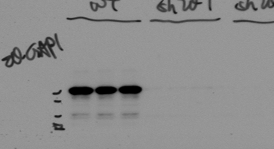

Supplement: Supplementary file 5 — Source Data for Expanded View [file EMBJ-42-e110780-s003.zip › Figure EV2/C/IQGAP1_WCL.tif]

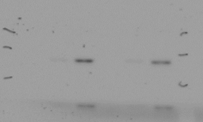

Supplement: Supplementary file 5 — Source Data for Expanded View [file EMBJ-42-e110780-s003.zip › Figure EV2/C/NEDD4.tif]

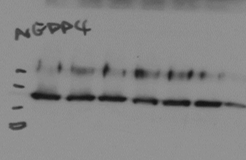

Supplement: Supplementary file 5 — Source Data for Expanded View [file EMBJ-42-e110780-s003.zip › Figure EV2/C/NEDD4_WCL.tif]

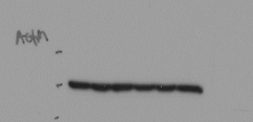

Supplement: Supplementary file 5 — Source Data for Expanded View [file EMBJ-42-e110780-s003.zip › Figure EV2/D/Actin_WCL.tif]

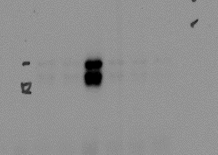

Supplement: Supplementary file 5 — Source Data for Expanded View [file EMBJ-42-e110780-s003.zip › Figure EV2/D/Alix.tif]

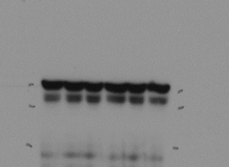

Supplement: Supplementary file 5 — Source Data for Expanded View [file EMBJ-42-e110780-s003.zip › Figure EV2/D/Alix_WCL.tif]

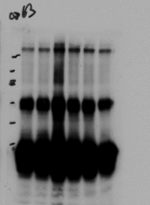

Supplement: Supplementary file 5 — Source Data for Expanded View [file EMBJ-42-e110780-s003.zip › Figure EV2/D/CD63.tif]

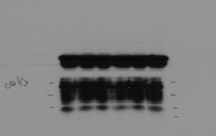

Supplement: Supplementary file 5 — Source Data for Expanded View [file EMBJ-42-e110780-s003.zip › Figure EV2/D/CD63_WCL.tif]

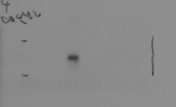

Supplement: Supplementary file 5 — Source Data for Expanded View [file EMBJ-42-e110780-s003.zip › Figure EV2/D/CDC42.tif]

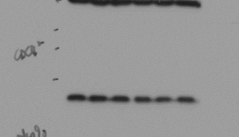

Supplement: Supplementary file 5 — Source Data for Expanded View [file EMBJ-42-e110780-s003.zip › Figure EV2/D/CDC42_WCL.tif]

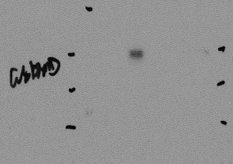

Supplement: Supplementary file 5 — Source Data for Expanded View [file EMBJ-42-e110780-s003.zip › Figure EV2/D/GSDMD.tif]

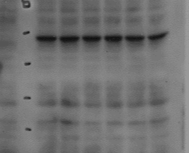

Supplement: Supplementary file 5 — Source Data for Expanded View [file EMBJ-42-e110780-s003.zip › Figure EV2/D/GSDMD_WCL.tif]

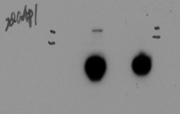

Supplement: Supplementary file 5 — Source Data for Expanded View [file EMBJ-42-e110780-s003.zip › Figure EV2/D/IQGAP1.tif]

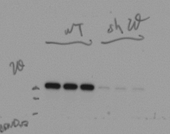

Supplement: Supplementary file 5 — Source Data for Expanded View [file EMBJ-42-e110780-s003.zip › Figure EV2/D/IQGAP1_WCL.tif]

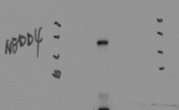

Supplement: Supplementary file 5 — Source Data for Expanded View [file EMBJ-42-e110780-s003.zip › Figure EV2/D/NEDD4.tif]

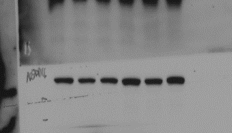

Supplement: Supplementary file 5 — Source Data for Expanded View [file EMBJ-42-e110780-s003.zip › Figure EV2/D/NEDD4_WCL.tif]

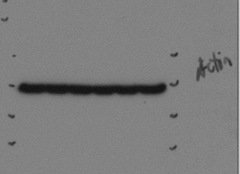

Supplement: Supplementary file 5 — Source Data for Expanded View [file EMBJ-42-e110780-s003.zip › Figure EV3/A/Actin_WCL.tif]

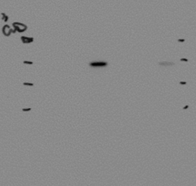

Supplement: Supplementary file 5 — Source Data for Expanded View [file EMBJ-42-e110780-s003.zip › Figure EV3/A/GSDMD.tif]

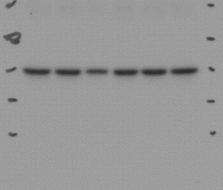

Supplement: Supplementary file 5 — Source Data for Expanded View [file EMBJ-42-e110780-s003.zip › Figure EV3/A/GSDMD_WCL.tif]

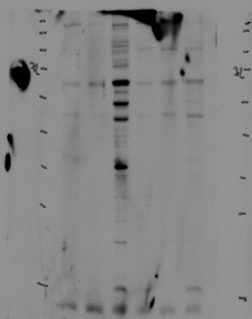

Supplement: Supplementary file 5 — Source Data for Expanded View [file EMBJ-42-e110780-s003.zip › Figure EV3/A/IL-1b.tif]

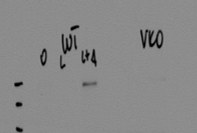

Supplement: Supplementary file 5 — Source Data for Expanded View [file EMBJ-42-e110780-s003.zip › Figure EV3/A/IQGAP1.tif]

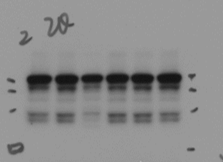

Supplement: Supplementary file 5 — Source Data for Expanded View [file EMBJ-42-e110780-s003.zip › Figure EV3/A/IQGAP1_WCL.tif]

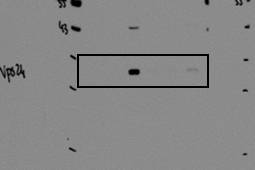

Supplement: Supplementary file 5 — Source Data for Expanded View [file EMBJ-42-e110780-s003.zip › Figure EV3/A/Vps24.tif]

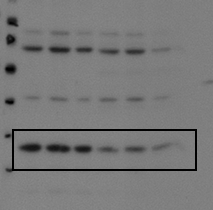

Supplement: Supplementary file 5 — Source Data for Expanded View [file EMBJ-42-e110780-s003.zip › Figure EV3/A/Vps24_WCL.tif]

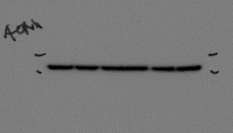

Supplement: Supplementary file 5 — Source Data for Expanded View [file EMBJ-42-e110780-s003.zip › Figure EV3/D/Actin_WCL.tif]

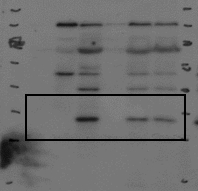

Supplement: Supplementary file 5 — Source Data for Expanded View [file EMBJ-42-e110780-s003.zip › Figure EV3/D/CD63.tif]

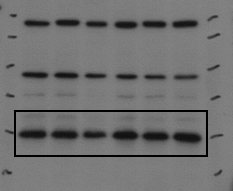

Supplement: Supplementary file 5 — Source Data for Expanded View [file EMBJ-42-e110780-s003.zip › Figure EV3/D/CD63_WCL.tif]

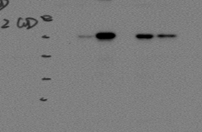

Supplement: Supplementary file 5 — Source Data for Expanded View [file EMBJ-42-e110780-s003.zip › Figure EV3/D/GSDMD.tif]

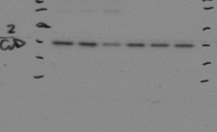

Supplement: Supplementary file 5 — Source Data for Expanded View [file EMBJ-42-e110780-s003.zip › Figure EV3/D/GSDMD_WCL.tif]

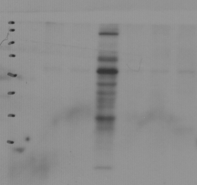

Supplement: Supplementary file 5 — Source Data for Expanded View [file EMBJ-42-e110780-s003.zip › Figure EV3/D/IL-1b.tif]

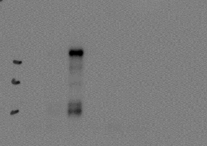

Supplement: Supplementary file 5 — Source Data for Expanded View [file EMBJ-42-e110780-s003.zip › Figure EV3/D/IQGAP1.tif]

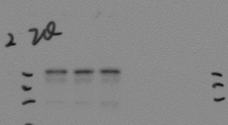

Supplement: Supplementary file 5 — Source Data for Expanded View [file EMBJ-42-e110780-s003.zip › Figure EV3/D/IQGAP1_WCL.tif]

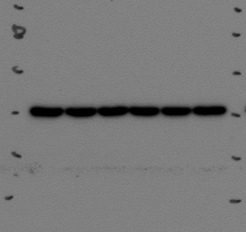

Supplement: Supplementary file 5 — Source Data for Expanded View [file EMBJ-42-e110780-s003.zip › Figure EV3/E/Actin_WCL.tif]

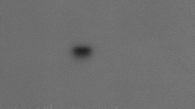

Supplement: Supplementary file 5 — Source Data for Expanded View [file EMBJ-42-e110780-s003.zip › Figure EV3/E/ASC.tif]

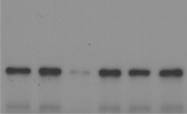

Supplement: Supplementary file 5 — Source Data for Expanded View [file EMBJ-42-e110780-s003.zip › Figure EV3/E/ASC_WCL.tif]

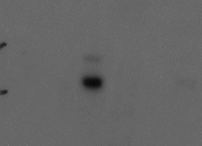

Supplement: Supplementary file 5 — Source Data for Expanded View [file EMBJ-42-e110780-s003.zip › Figure EV3/E/Casp8.tif]

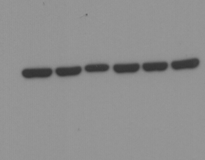

Supplement: Supplementary file 5 — Source Data for Expanded View [file EMBJ-42-e110780-s003.zip › Figure EV3/E/Casp8_WCL.tif]

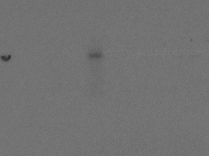

Supplement: Supplementary file 5 — Source Data for Expanded View [file EMBJ-42-e110780-s003.zip › Figure EV3/E/GSDMD.tif]

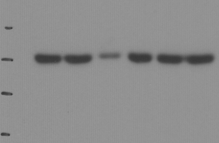

Supplement: Supplementary file 5 — Source Data for Expanded View [file EMBJ-42-e110780-s003.zip › Figure EV3/E/GSDMD_WCL.tif]

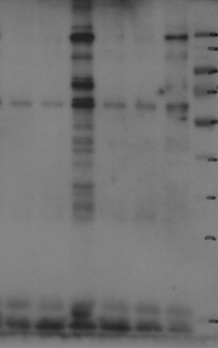

Supplement: Supplementary file 5 — Source Data for Expanded View [file EMBJ-42-e110780-s003.zip › Figure EV3/E/IL-1b.tif]

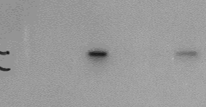

Supplement: Supplementary file 5 — Source Data for Expanded View [file EMBJ-42-e110780-s003.zip › Figure EV3/E/IQGAP1.tif]

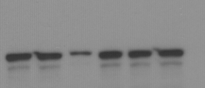

Supplement: Supplementary file 5 — Source Data for Expanded View [file EMBJ-42-e110780-s003.zip › Figure EV3/E/IQGAP1_WCL.tif]

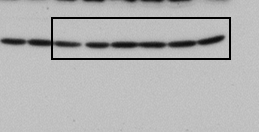

Supplement: Supplementary file 7 — Source Data for Figure 1 [file EMBJ-42-e110780-s002.zip › Figure 1/A/Actin_WCL.tif]

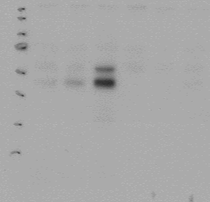

Supplement: Supplementary file 7 — Source Data for Figure 1 [file EMBJ-42-e110780-s002.zip › Figure 1/A/Casp8.tif]

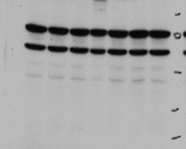

Supplement: Supplementary file 7 — Source Data for Figure 1 [file EMBJ-42-e110780-s002.zip › Figure 1/A/Casp8_WCL.tif]

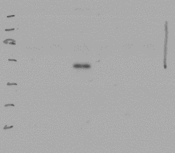

Supplement: Supplementary file 7 — Source Data for Figure 1 [file EMBJ-42-e110780-s002.zip › Figure 1/A/CDC37.tif]

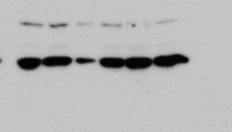

Supplement: Supplementary file 7 — Source Data for Figure 1 [file EMBJ-42-e110780-s002.zip › Figure 1/A/CDC37_WCL.tif]

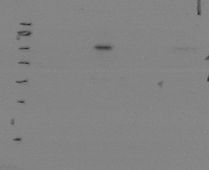

Supplement: Supplementary file 7 — Source Data for Figure 1 [file EMBJ-42-e110780-s002.zip › Figure 1/A/GSDMD.tif]

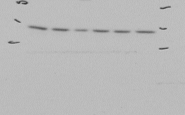

Supplement: Supplementary file 7 — Source Data for Figure 1 [file EMBJ-42-e110780-s002.zip › Figure 1/A/GSDMD_WCL.tif]
