# Supplementary figures and images for: The Ras GTPase‐activating‐like protein IQGAP1 bridges Gasdermin D to the ESCRT system to promote IL‐1β release via exosomes (part 2 of 3)
Source: EMBO J. 2022 Nov 14;42(1):e110780. doi: 10.15252/embj.2022110780 (PMC9811620; doi:10.15252/embj.2022110780)

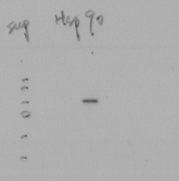

Supplement: Supplementary file 7 — Source Data for Figure 1 [file EMBJ-42-e110780-s002.zip › Figure 1/A/Hsp90.tif]

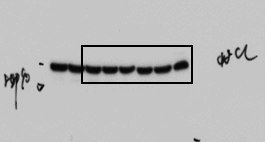

Supplement: Supplementary file 7 — Source Data for Figure 1 [file EMBJ-42-e110780-s002.zip › Figure 1/A/Hsp90_WCL.tif]

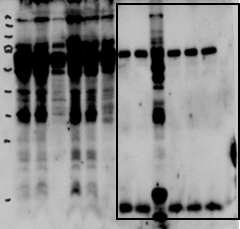

Supplement: Supplementary file 7 — Source Data for Figure 1 [file EMBJ-42-e110780-s002.zip › Figure 1/A/IL-1b.tif]

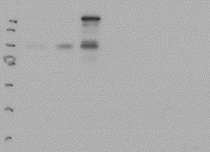

Supplement: Supplementary file 7 — Source Data for Figure 1 [file EMBJ-42-e110780-s002.zip › Figure 1/A/IQGAP1.tif]

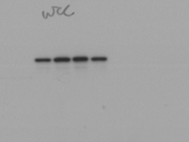

Supplement: Supplementary file 7 — Source Data for Figure 1 [file EMBJ-42-e110780-s002.zip › Figure 1/A/IQGAP1_WCL.tif]

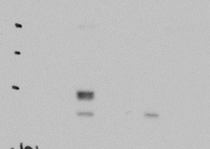

Supplement: Supplementary file 7 — Source Data for Figure 1 [file EMBJ-42-e110780-s002.zip › Figure 1/A/NEDD4.tif]

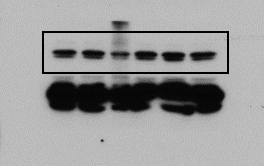

Supplement: Supplementary file 7 — Source Data for Figure 1 [file EMBJ-42-e110780-s002.zip › Figure 1/A/NEDD4_WCL.tif]

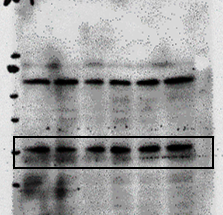

Supplement: Supplementary file 7 — Source Data for Figure 1 [file EMBJ-42-e110780-s002.zip › Figure 1/A/Pro-IL1b.tif]

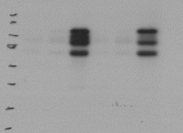

Supplement: Supplementary file 7 — Source Data for Figure 1 [file EMBJ-42-e110780-s002.zip › Figure 1/D/Alix.tif]

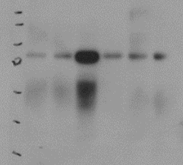

Supplement: Supplementary file 7 — Source Data for Figure 1 [file EMBJ-42-e110780-s002.zip › Figure 1/D/CD63.tif]

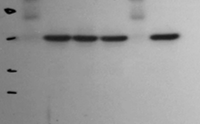

Supplement: Supplementary file 8 — Source Data for Figure 2 [file EMBJ-42-e110780-s013.zip › Figure 2/B/Flag.tif]

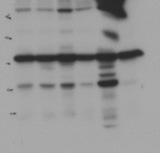

Supplement: Supplementary file 8 — Source Data for Figure 2 [file EMBJ-42-e110780-s013.zip › Figure 2/B/Flag_WCL.tif]

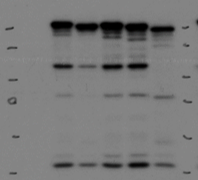

Supplement: Supplementary file 8 — Source Data for Figure 2 [file EMBJ-42-e110780-s013.zip › Figure 2/B/Myc.tif]

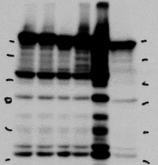

Supplement: Supplementary file 8 — Source Data for Figure 2 [file EMBJ-42-e110780-s013.zip › Figure 2/B/Myc_WCL.tif]

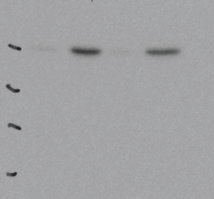

Supplement: Supplementary file 8 — Source Data for Figure 2 [file EMBJ-42-e110780-s013.zip › Figure 2/C/HA.tif]

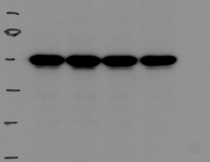

Supplement: Supplementary file 8 — Source Data for Figure 2 [file EMBJ-42-e110780-s013.zip › Figure 2/C/HA_WCL.tif]

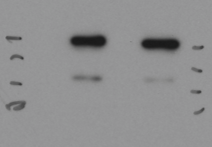

Supplement: Supplementary file 8 — Source Data for Figure 2 [file EMBJ-42-e110780-s013.zip › Figure 2/C/Myc.tif]

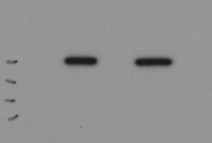

Supplement: Supplementary file 8 — Source Data for Figure 2 [file EMBJ-42-e110780-s013.zip › Figure 2/C/Myc_WCL.tif]

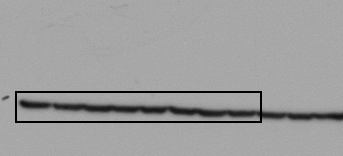

Supplement: Supplementary file 8 — Source Data for Figure 2 [file EMBJ-42-e110780-s013.zip › Figure 2/D/Actin_WCL.tif]

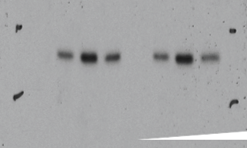

Supplement: Supplementary file 8 — Source Data for Figure 2 [file EMBJ-42-e110780-s013.zip › Figure 2/D/GSDMD.tif]

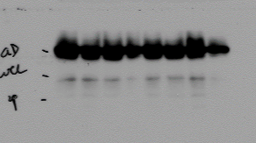

Supplement: Supplementary file 8 — Source Data for Figure 2 [file EMBJ-42-e110780-s013.zip › Figure 2/D/GSDMD_WCL.tif]

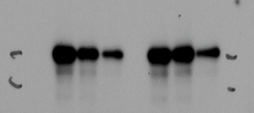

Supplement: Supplementary file 8 — Source Data for Figure 2 [file EMBJ-42-e110780-s013.zip › Figure 2/D/IQGAP1.tif]

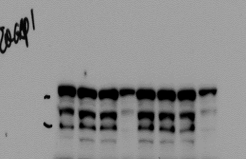

Supplement: Supplementary file 8 — Source Data for Figure 2 [file EMBJ-42-e110780-s013.zip › Figure 2/D/IQGAP1_WCL.tif]

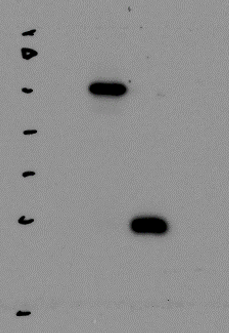

Supplement: Supplementary file 8 — Source Data for Figure 2 [file EMBJ-42-e110780-s013.zip › Figure 2/E/HA.tif]

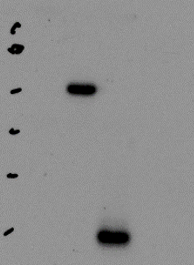

Supplement: Supplementary file 8 — Source Data for Figure 2 [file EMBJ-42-e110780-s013.zip › Figure 2/E/HA_WCL.tif]

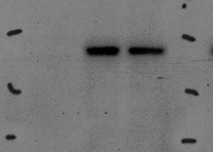

Supplement: Supplementary file 8 — Source Data for Figure 2 [file EMBJ-42-e110780-s013.zip › Figure 2/E/Myc.tif]

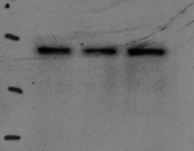

Supplement: Supplementary file 8 — Source Data for Figure 2 [file EMBJ-42-e110780-s013.zip › Figure 2/E/Myc_WCL.tif]

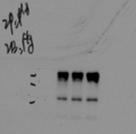

Supplement: Supplementary file 8 — Source Data for Figure 2 [file EMBJ-42-e110780-s013.zip › Figure 2/F/Flag.tif]

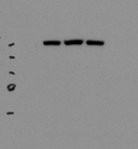

Supplement: Supplementary file 8 — Source Data for Figure 2 [file EMBJ-42-e110780-s013.zip › Figure 2/F/Flag_WCL.tif]

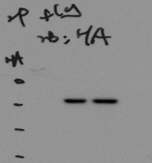

Supplement: Supplementary file 8 — Source Data for Figure 2 [file EMBJ-42-e110780-s013.zip › Figure 2/F/HA.tif]

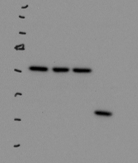

Supplement: Supplementary file 8 — Source Data for Figure 2 [file EMBJ-42-e110780-s013.zip › Figure 2/F/HA_WCL.tif]

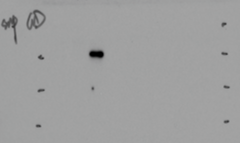

Supplement: Supplementary file 9 — Source Data for Figure 3 [file EMBJ-42-e110780-s010.zip › Figure 3/D/GSDMD.tif]

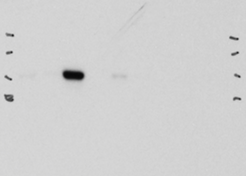

Supplement: Supplementary file 9 — Source Data for Figure 3 [file EMBJ-42-e110780-s010.zip › Figure 3/D/Hsp90.tif]

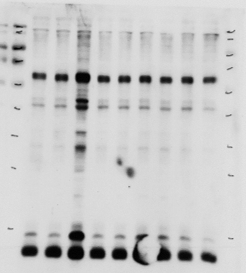

Supplement: Supplementary file 9 — Source Data for Figure 3 [file EMBJ-42-e110780-s010.zip › Figure 3/D/IL-1b.tif]

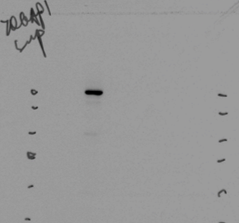

Supplement: Supplementary file 9 — Source Data for Figure 3 [file EMBJ-42-e110780-s010.zip › Figure 3/D/IQGAP1.tif]

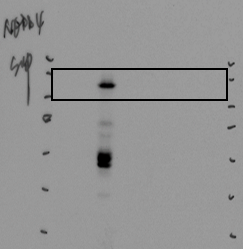

Supplement: Supplementary file 9 — Source Data for Figure 3 [file EMBJ-42-e110780-s010.zip › Figure 3/D/NEDD4.tif]

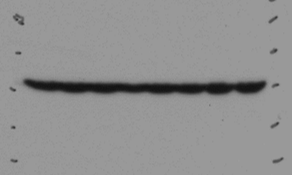

Supplement: Supplementary file 9 — Source Data for Figure 3 [file EMBJ-42-e110780-s010.zip › Figure 3/E/Actin.tif]

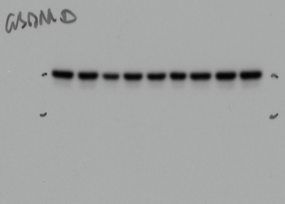

Supplement: Supplementary file 9 — Source Data for Figure 3 [file EMBJ-42-e110780-s010.zip › Figure 3/E/GSDMD.tif]

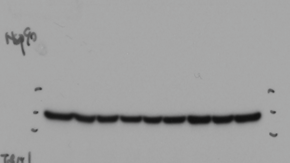

Supplement: Supplementary file 9 — Source Data for Figure 3 [file EMBJ-42-e110780-s010.zip › Figure 3/E/Hsp90.tif]

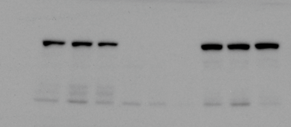

Supplement: Supplementary file 9 — Source Data for Figure 3 [file EMBJ-42-e110780-s010.zip › Figure 3/E/IQGAP1.tif]

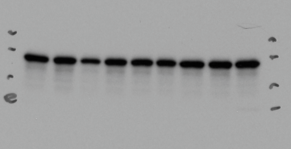

Supplement: Supplementary file 9 — Source Data for Figure 3 [file EMBJ-42-e110780-s010.zip › Figure 3/E/NEDD4.tif]

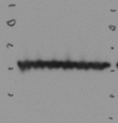

Supplement: Supplementary file 10 — Source Data for Figure 4 [file EMBJ-42-e110780-s009.zip › Figure 4/F/Actin.tif]

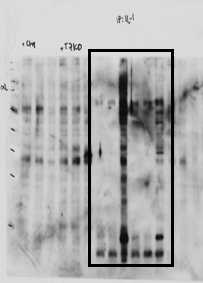

Supplement: Supplementary file 10 — Source Data for Figure 4 [file EMBJ-42-e110780-s009.zip › Figure 4/F/IL-1b.tif]

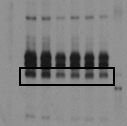

Supplement: Supplementary file 10 — Source Data for Figure 4 [file EMBJ-42-e110780-s009.zip › Figure 4/F/Pro-IL-1b.tif]

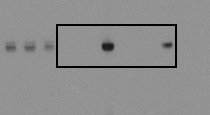

Supplement: Supplementary file 10 — Source Data for Figure 4 [file EMBJ-42-e110780-s009.zip › Figure 4/F/Tsg101.tif]

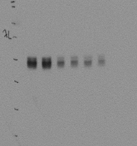

Supplement: Supplementary file 10 — Source Data for Figure 4 [file EMBJ-42-e110780-s009.zip › Figure 4/F/Tsg101_Lysate.tif]

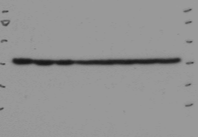

Supplement: Supplementary file 10 — Source Data for Figure 4 [file EMBJ-42-e110780-s009.zip › Figure 4/H/Actin.tif]

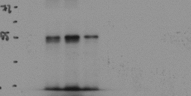

Supplement: Supplementary file 10 — Source Data for Figure 4 [file EMBJ-42-e110780-s009.zip › Figure 4/H/GSDMD.tif]

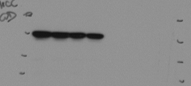

Supplement: Supplementary file 10 — Source Data for Figure 4 [file EMBJ-42-e110780-s009.zip › Figure 4/H/GSDMD_WCL.tif]

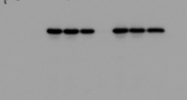

Supplement: Supplementary file 10 — Source Data for Figure 4 [file EMBJ-42-e110780-s009.zip › Figure 4/H/IQGAP1.tif]

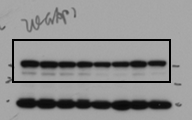

Supplement: Supplementary file 10 — Source Data for Figure 4 [file EMBJ-42-e110780-s009.zip › Figure 4/H/IQGAP1_WCL.tif]

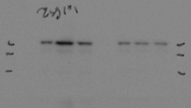

Supplement: Supplementary file 10 — Source Data for Figure 4 [file EMBJ-42-e110780-s009.zip › Figure 4/H/Tsg101.tif]

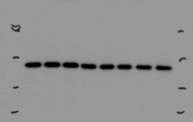

Supplement: Supplementary file 10 — Source Data for Figure 4 [file EMBJ-42-e110780-s009.zip › Figure 4/H/Tsg101_WCL.tif]

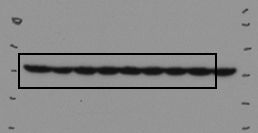

Supplement: Supplementary file 10 — Source Data for Figure 4 [file EMBJ-42-e110780-s009.zip › Figure 4/I/Actin_WCL.tif]

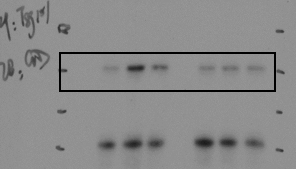

Supplement: Supplementary file 10 — Source Data for Figure 4 [file EMBJ-42-e110780-s009.zip › Figure 4/I/GSDMD.tif]

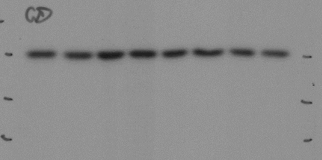

Supplement: Supplementary file 10 — Source Data for Figure 4 [file EMBJ-42-e110780-s009.zip › Figure 4/I/GSDMD_WCL.tif]

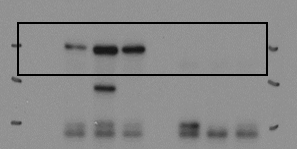

Supplement: Supplementary file 10 — Source Data for Figure 4 [file EMBJ-42-e110780-s009.zip › Figure 4/I/IQGAP1.tif]

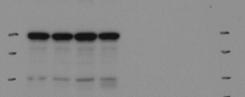

Supplement: Supplementary file 10 — Source Data for Figure 4 [file EMBJ-42-e110780-s009.zip › Figure 4/I/IQGAP1_WCL.tif]

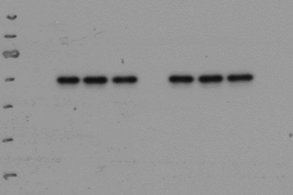

Supplement: Supplementary file 10 — Source Data for Figure 4 [file EMBJ-42-e110780-s009.zip › Figure 4/I/Tsg101.tif]

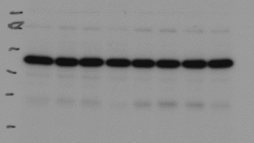

Supplement: Supplementary file 10 — Source Data for Figure 4 [file EMBJ-42-e110780-s009.zip › Figure 4/I/Tsg101_WCL.tif]

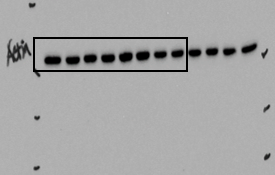

Supplement: Supplementary file 11 — Source Data for Figure 5 [file EMBJ-42-e110780-s005.zip › Figure 5/D/Actin_WCL.tif]

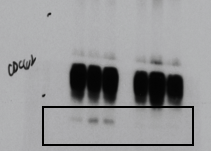

Supplement: Supplementary file 11 — Source Data for Figure 5 [file EMBJ-42-e110780-s005.zip › Figure 5/D/CDC42.tif]

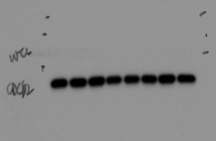

Supplement: Supplementary file 11 — Source Data for Figure 5 [file EMBJ-42-e110780-s005.zip › Figure 5/D/CDC42_WCL.tif]

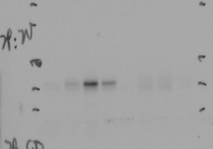

Supplement: Supplementary file 11 — Source Data for Figure 5 [file EMBJ-42-e110780-s005.zip › Figure 5/D/GSDMD.tif]

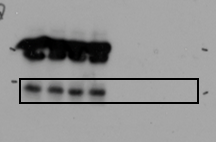

Supplement: Supplementary file 11 — Source Data for Figure 5 [file EMBJ-42-e110780-s005.zip › Figure 5/D/GSDMD_WCL.tif]

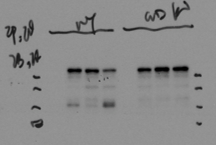

Supplement: Supplementary file 11 — Source Data for Figure 5 [file EMBJ-42-e110780-s005.zip › Figure 5/D/IQGAP1.tif]

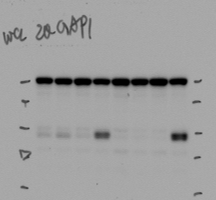

Supplement: Supplementary file 11 — Source Data for Figure 5 [file EMBJ-42-e110780-s005.zip › Figure 5/D/IQGAP1_WCL.tif]

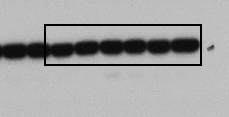

Supplement: Supplementary file 11 — Source Data for Figure 5 [file EMBJ-42-e110780-s005.zip › Figure 5/E/Actin_WCL.tif]

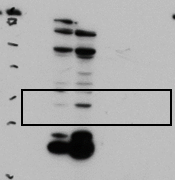

Supplement: Supplementary file 11 — Source Data for Figure 5 [file EMBJ-42-e110780-s005.zip › Figure 5/E/CDC42.tif]

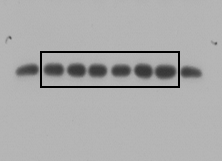

Supplement: Supplementary file 11 — Source Data for Figure 5 [file EMBJ-42-e110780-s005.zip › Figure 5/E/CDC42_WCL.tif]

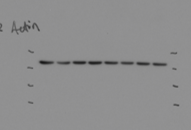

Supplement: Supplementary file 11 — Source Data for Figure 5 [file EMBJ-42-e110780-s005.zip › Figure 5/F/Actin_WCL.tif]

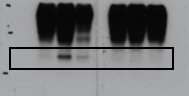

Supplement: Supplementary file 11 — Source Data for Figure 5 [file EMBJ-42-e110780-s005.zip › Figure 5/F/CDC42.tif]

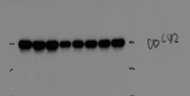

Supplement: Supplementary file 11 — Source Data for Figure 5 [file EMBJ-42-e110780-s005.zip › Figure 5/F/CDC42_WCL.tif]

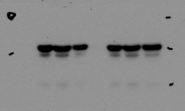

Supplement: Supplementary file 11 — Source Data for Figure 5 [file EMBJ-42-e110780-s005.zip › Figure 5/F/GSDMD.tif]

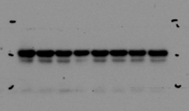

Supplement: Supplementary file 11 — Source Data for Figure 5 [file EMBJ-42-e110780-s005.zip › Figure 5/F/GSDMD_WCL.tif]

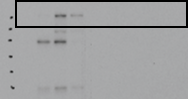

Supplement: Supplementary file 11 — Source Data for Figure 5 [file EMBJ-42-e110780-s005.zip › Figure 5/F/IQGAP1.tif]

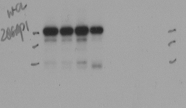

Supplement: Supplementary file 11 — Source Data for Figure 5 [file EMBJ-42-e110780-s005.zip › Figure 5/F/IQGAP1_WCL.tif]

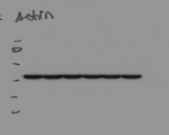

Supplement: Supplementary file 11 — Source Data for Figure 5 [file EMBJ-42-e110780-s005.zip › Figure 5/G/Actin_WCL.tif]

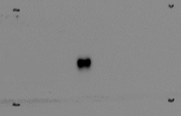

Supplement: Supplementary file 11 — Source Data for Figure 5 [file EMBJ-42-e110780-s005.zip › Figure 5/G/CDC42.tif]

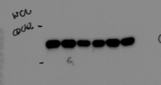

Supplement: Supplementary file 11 — Source Data for Figure 5 [file EMBJ-42-e110780-s005.zip › Figure 5/G/CDC42_WCL.tif]

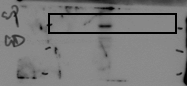

Supplement: Supplementary file 11 — Source Data for Figure 5 [file EMBJ-42-e110780-s005.zip › Figure 5/G/GSDMD.tif]

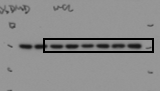

Supplement: Supplementary file 11 — Source Data for Figure 5 [file EMBJ-42-e110780-s005.zip › Figure 5/G/GSDMD_WCL.tif]

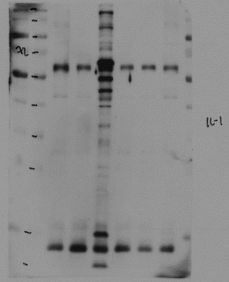

Supplement: Supplementary file 11 — Source Data for Figure 5 [file EMBJ-42-e110780-s005.zip › Figure 5/G/IL-1b.tif]

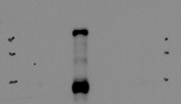

Supplement: Supplementary file 11 — Source Data for Figure 5 [file EMBJ-42-e110780-s005.zip › Figure 5/G/IQGAP1.tif]

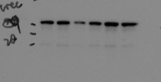

Supplement: Supplementary file 11 — Source Data for Figure 5 [file EMBJ-42-e110780-s005.zip › Figure 5/G/IQGAP1_WCL.tif]

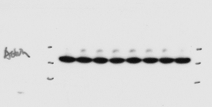

Supplement: Supplementary file 11 — Source Data for Figure 5 [file EMBJ-42-e110780-s005.zip › Figure 5/I/Actin_WCL.tif]

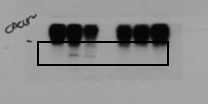

Supplement: Supplementary file 11 — Source Data for Figure 5 [file EMBJ-42-e110780-s005.zip › Figure 5/I/CDC42.tif]

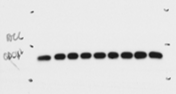

Supplement: Supplementary file 11 — Source Data for Figure 5 [file EMBJ-42-e110780-s005.zip › Figure 5/I/CDC42_WCL.tif]

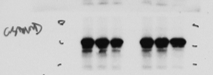

Supplement: Supplementary file 11 — Source Data for Figure 5 [file EMBJ-42-e110780-s005.zip › Figure 5/I/GSDMD.tif]

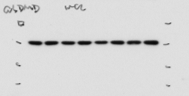

Supplement: Supplementary file 11 — Source Data for Figure 5 [file EMBJ-42-e110780-s005.zip › Figure 5/I/GSDMD_WCL.tif]

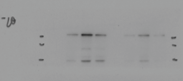

Supplement: Supplementary file 11 — Source Data for Figure 5 [file EMBJ-42-e110780-s005.zip › Figure 5/I/IQGAP1.tif]

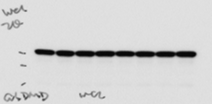

Supplement: Supplementary file 11 — Source Data for Figure 5 [file EMBJ-42-e110780-s005.zip › Figure 5/I/IQGAP1_WCL.tif]

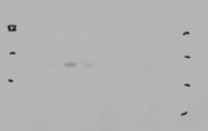

Supplement: Supplementary file 11 — Source Data for Figure 5 [file EMBJ-42-e110780-s005.zip › Figure 5/I/Tsg101.tif]

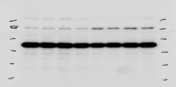

Supplement: Supplementary file 11 — Source Data for Figure 5 [file EMBJ-42-e110780-s005.zip › Figure 5/I/Tsg101_WCL.tif]

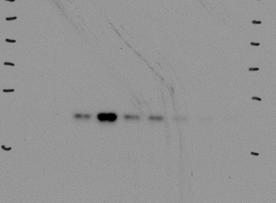

Supplement: Supplementary file 12 — Source Data for Figure 6 [file EMBJ-42-e110780-s011.zip › Figure 6/C/GSDMD KO/CDC42.tif]

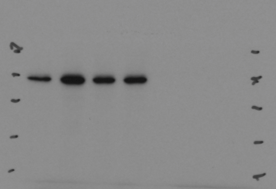

Supplement: Supplementary file 12 — Source Data for Figure 6 [file EMBJ-42-e110780-s011.zip › Figure 6/C/GSDMD KO/GSDMD.tif]

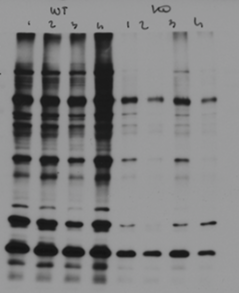

Supplement: Supplementary file 12 — Source Data for Figure 6 [file EMBJ-42-e110780-s011.zip › Figure 6/C/GSDMD KO/IL-1b.tif]

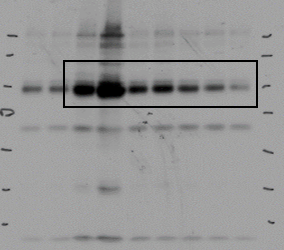

Supplement: Supplementary file 12 — Source Data for Figure 6 [file EMBJ-42-e110780-s011.zip › Figure 6/C/GSDMD KO/IQGAP1.tif]

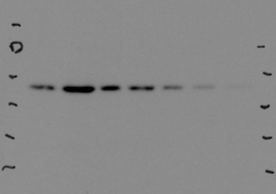

Supplement: Supplementary file 12 — Source Data for Figure 6 [file EMBJ-42-e110780-s011.zip › Figure 6/C/GSDMD KO/Tsg101.tif]

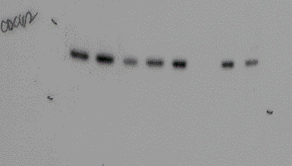

Supplement: Supplementary file 12 — Source Data for Figure 6 [file EMBJ-42-e110780-s011.zip › Figure 6/C/IQGAP1 KO/CDC42.tif]
