# Supplementary figures and images for: The Ras GTPase‐activating‐like protein IQGAP1 bridges Gasdermin D to the ESCRT system to promote IL‐1β release via exosomes (part 3 of 3)
Source: EMBO J. 2022 Nov 14;42(1):e110780. doi: 10.15252/embj.2022110780 (PMC9811620; doi:10.15252/embj.2022110780)

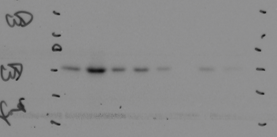

Supplement: Supplementary file 12 — Source Data for Figure 6 [file EMBJ-42-e110780-s011.zip › Figure 6/C/IQGAP1 KO/GSDMD.tif]

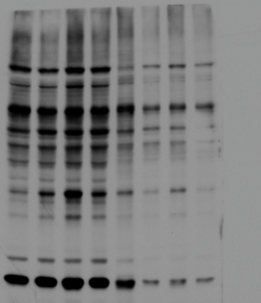

Supplement: Supplementary file 12 — Source Data for Figure 6 [file EMBJ-42-e110780-s011.zip › Figure 6/C/IQGAP1 KO/IL-1b.tif]

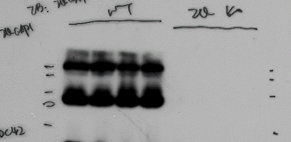

Supplement: Supplementary file 12 — Source Data for Figure 6 [file EMBJ-42-e110780-s011.zip › Figure 6/C/IQGAP1 KO/IQGAP1.tif]

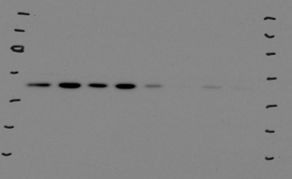

Supplement: Supplementary file 12 — Source Data for Figure 6 [file EMBJ-42-e110780-s011.zip › Figure 6/C/IQGAP1 KO/Tsg101.tif]
