# Supplementary material for: The Ras GTPase‐activating‐like protein IQGAP1 bridges Gasdermin D to the ESCRT system to promote IL‐1β release via exosomes
Source: EMBO J. 2022 Nov 14;42(1):e110780. doi: 10.15252/embj.2022110780 (PMC9811620; doi:10.15252/embj.2022110780)
Supplement: Supplementary file 13 [file EMBJ-42-e110780-s008.pdf]

THE EMBO JOURNAL

The Ras GTPase-activating-like protein IQGAP1 bridges Gasdermin D to the ESCRT system to promote IL-1β release via exosomes

Yun Liao, Xing Chen, William Miller-Little, Han Wang, Belinda Willard, Katarzyna Bulek, Junjie Zhao, and Xiaoxia Li
DOI: 10.15252/embj.2022110780

Corresponding author(s): Katarzyna Bulek (bulekk@ccf.org) , Xiaoxia Li (xli73337@gmail.com), Junjie Zhao (junjie.zhao@case.edu)

Review Timeline:

Submission Date:	28th Jan 22
Correspondence:	18th Mar 22
Editorial Decision:	31st Mar 22
Revision Received:	23rd Jul 22
Editorial Decision:	16th Aug 22
Revision Received:	24th Aug 22
Editorial Decision:	14th Sep 22
Revision Received:	23rd Sep 22
Accepted:	18th Oct 22

Editor: Karin Dumstrei

Transaction Report:

(Note: With the exception of the correction of typographical or spelling errors that could be a source of ambiguity, letters and reports are not edited. Depending on transfer agreements, referee reports obtained elsewhere may or may not be included in this compilation. Referee reports are anonymous unless the Referee chooses to sign their reports.)

Dear Katarzyna,

Thank you for submitting your manuscript to The EMBO Journal. I am sorry for the delay in getting back to you with a decision, but I have now received the two referee reports on the study.

As you can see from the comments below there is an interest in the study. However, the referees also raise significant concerns with the some of the data supporting the key conclusions. Referee #1 raises issues regarding the biological activity measurements of IL-1β as well as image quantifications. While referee #2 requests further insight into upstream events leading to IL-1 secretion.

As it is unclear if these issues can be resolved in a revised version, I think that it is most efficient to ask for a point-by-point response upfront to see what type of experiments you could introduce in a revised version to strengthen the conclusions. Based upon this point-by-point response I will then take the decision.

Looking forward to hearing back from you

With best wishes

Karin

Karin Dumstrei, PhD
Senior Editor
The EMBO Journal

Referee #1

In this study Liao et al., report that IQGAP1 bridges GSDMD to the ESCRT system to promote IL-1β release via exosomes. The proposal is that IQGAP1 function as a bridge connecting GSDMD to ESCRT machinery to promote ubiquitinated-IL-1β and/or pro-IL-1β containing exosomes in response to inflammasome activation. The authors also propose that IQGAP1-GSDMD interaction recruits TSG101 on GSDMD+ late endosome to drive exosomal IL-1β release. In this context CDC42 serves as a molecular switch and enables IQGAP1 to bridge GSDMD to TSG101 during inflammasome-triggered exosome biogenesis.

Some of the proposed relatioships seem to be shown in isolation of other mainstream processes of biologically-active v release. The exosome relationships while tantalizing are quite teneous, and it is difficult to assess how significant they are. In the absence of showing that this pathway releases biologically active IL-1β engaged in signaling at target cells (the authors have no assays for activity of IL-1β) the significance of these observations is unclear.

Major:

1. In the introduction, the authors sate that exosomes have been established as exit a mechanism of pyroptosis-associated release of IL-1β referencing a 2007 study. Much has happened since that time and gasdermin pores at the plasma membrane have been exceptionally well defined and accepted as the main pyroptotic mechanism of IL-1β release. The study, if it were to be published, needs to compare the exosomal cargo vs plasma membrane pore release to make sense

2. There are multiple problems with key data as outlined in points below, but the most important issue is the biological activity - if any - of the releasedIL-1β via this proposed exit pathway has not been addressed. The essays used are detecting IL-1β. But is it biologically active? This needs to be properly tested. Otherwise this is if anything a disposal pathway.

3. The interactions between IQGAP1 and GSDMD are shown by co-IPs nut no direct interaction has been established. Appropriate methods should be used to address and establish (rule in or out) that. Importantly, the IQGAP1 could be swept into large protein complexes with ESCRTs engaged in countering plasma membrane permeabilization, a well-established process.

4. Exosome and extracellular vesicles release accompany plasma membrane damage caused by GSDMD or other agents. This is a well known phenomenon, and yet the authors do not account or address any of that.

5. Others have shown that IQGAP1 in cooperation with TSG101 and other ESCRT protesin protects against GSDMD pores at the plasma membrane. It is possible that what the authors here are reporting what is actually a part of the previously published processes at the plasma membrane during GSDMD pore formation and pyroptotic plasmalemma permeabilization. This needs to be fully and formally addressed.

6. Further problems with key data: the 'endosomal localization' (?) in Fig. 4 is based on poor imaging and absence of quantification. (a) The only quantification is with EEA1 (early endosomal marker, not MVBs) in panel A, and not with CD63.

7. Along the same lines, the images in Fig4B with CD63 "show" some overlaps in yellow, but there are so many dots (background fluorescence ?) with IQGAP1 that one wonders if the authors were to flip by 180 degrees yellow and green images (randomizing) would the number of yellow dots remain the same... No quantification/statistics of any kind is a big issue here.

8. TSG101 is an upstream ESCRT in ESCRT machinery which recognizes the cargo protein but doesn't directly regulate the membrane remodeling. Did authors check other ESCRT proteins?

9. In the abstract the authors mention that IQGAP1 bridges GSDMD to ESCRT machinery to promote IL-1β containing exosomes in response to NLRP3 inflammasome activation but haven't shown any data on NLRP3 in this study.

10. Is IL-1β biologically active?

11. If increased in exosomes, how does IL-1β contact the receptors on cells to achieve its biliogical activity?

12. There are some known paradoxical relationship with GTP and GDP states known regarding IQGAPs and Rho GTPases. It seems that the authors may not be aware of those and believe that GTP state (typically associated with "active" small GTPases) is the ON state. This needs to be resolved. Also, IQGAPs are calmodcuin binding protins and Ca2+ may play a role...

Minor:
1. Please check the sizes of graphs in figure 3A
2. Figure 3E and F and figure 4F graphs are represented differently.
3. In figure 6A author haven't pointed out specific structure to look.
4. On any of the western blot author haven't shown the marker to represent the specific size of the proteins.

Referee #2

Liao et al. (the authors) identify IQGAP1 as a potential interaction partner of gasdermin-D (GSDMD). They show that interaction between IQGAP1 and the C-terminal region of GSDMD is required for secretion of exosomes from YAMC cells upon treatment with LPS+ATP. These exosomes(or the intracellular complexes preceding secretion) contain IQGAP1, GSDMD and among other components TSG101 and activated CDC42. These complexes assemble in CD63 positive endosomes. Ablation of TSG101 expression or inhibition of CDC42 reduced secretion of exosomes upon LPS&ATP treatment. Furthermore, stable assembly of the CDC42, IQGAP1, GSDMD complex requires presence of both IQGAP1 or GSDMD, suggesting a stabilizing function for either proteins. The authors finish their study with the demonstration that exosome secretion is reduced in colonic explants from IQGAP1 or GSDMD KO treated with DSS.

I think the data presented are very interesting and propose a non-canonical role for GSDMD in IL-1 secretion (besides pore formation). The paper is well written, and the experiments are very well controlled. I think most of the conclusion presented are substantiated by the data presented (except detailed below).

My major concern is regarding the upstream events leading to IL-1 secretion in exosomes. We know that stimulation of cells with ATP leads to (despite other events) opening of the pannexin-1 channel by P2X7 stimulation and ion fluxes across the plasma membrane, which are responsible for inflammasome activation. The authors show the presence of cleaved IL-1 beta in the supernatant but cleavage of GSDMD can't be observed (or at least not shown in the study). We don't know if YAMC do activate NLRP3 and if all the processes shown are dependent on inflammasome activation, although the authors claim this in the text. Calcium influx across pannexin-1 channels can be envisioned to lead to activation of CDC42. IQGAP1 is, after all, a calcium responsive protein. Some experiment I would suggest to address questions concerning this knowledge gap in the study:
1. Use MCC950 or 90 mM extracellular KCl to check if secretion of exosomes is dependent on NLRP3 activation
2. Use zVAD-fmk or qVD to check if secretion is dependent on caspase-1 activation
3. Use extracellular EDTA or BAPTA-AM to check if exosome secretion or IQGAP1/GSDMD interaction is dependent on calcium influx

Minor suggestions:
Figure 3: Can the authors show a PLA assay between IQGAP1 ΔIQ and GSDMD as an independent confirmation of abolished interaction?
Figure 4C/D: Quantification of PLA is missing. Is it possible to stain for pro-IL beta to see colocalization with CD63 in wildtype and IQGAP1 KO cells?

1st Editorial Decision **31st Mar 2022**

Dear Kate,

Thank you for sending me your detailed point-by-point response of what experiments you can incorporate to address the raised concerns. I have now had a chance to take a careful look at everything and I appreciate the proposed experiments. Given this, I would therefore like to invite you to submit a revised version.

Let me know if we need to discuss anything further.

When preparing your letter of response to the referees' comments, please bear in mind that this will form part of the Review Process File, and will therefore be available online to the community. For more details on our Transparent Editorial Process, please visit our website: https://www.embopress.org/page/journal/14602075/authorguide#transparentprocess

We generally allow three months as standard revision time. As a matter of policy, competing manuscripts published during this period will not negatively impact on our assessment of the conceptual advance presented by your study. However, we request that you contact the editor as soon as possible upon publication of any related work, to discuss how to proceed. Should you foresee a problem in meeting this three-month deadline, please let us know in advance and we may be able to grant an extension.

Thank you for the opportunity to consider your work for publication. I look forward to your revision.

with best wishes

Karin

Karin Dumstrei, PhD
Senior Editor
The EMBO Journal

Instructions for preparing your revised manuscript:

Please see attached document for helpful tips on how to prepare the revised version.

Guide For Authors: https://www.embopress.org/page/journal/14602075/authorguide

We realize that it is difficult to revise to a specific deadline. In the interest of protecting the conceptual advance provided by the work, we recommend a revision within 3 months (29th Jun 2022). However, we can also easily extend this timeframe if needed. Please discuss the revision progress ahead of this time with the editor if you require more time to complete the revisions. Use the link below to submit your revision:

https://emboj.msubmit.net/cgi-bin/main.plex

Referee #1:

In this study Liao et al., report that IQGAP1 bridges GSDMD to the ESCRT system to promote IL-1β release via exosomes. The proposal is that IQGAP1 function as a bridge connecting GSDMD to ESCRT machinery to promote ubiquitinated-IL-1β and/or pro-IL-1β containing exosomes in response to inflammasome activation. The authors also propose that IQGAP1-GSDMD interaction recruits TSG101 on GSDMD+ late endosome to drive exosomal IL-1β release. In this context CDC42 serves as a molecular switch and enables IQGAP1 to bridge GSDMD to TSG101 during inflammasome-triggered exosome biogenesis.

Some of the proposed relatioships seem to be shown in isolation of other mainstream processes of biologically-active v release. The exosome relationships while tantalizing are quite teneous, and it is difficult to assess how significant they are. In the absence of showing that this pathway releases biologically active IL-1β engaged in signaling at target cells (the authors have no assays for activity of IL-1β) the significance of these observations is unclear.

Major:

1. In the introduction, the authors sate that exosomes have been established as exit a mechanism of pyroptosis-associated

release of IL-1β referencing a 2007 study. Much has happened since that time and gasdermin pores at the plasma membrane have been exceptionally well defined and accepted as the main pyroptotic mechanism of IL-1β release. The study, if it were to be published, needs to compare the exosomal cargo vs plasma membrane pore release to make sense

2. There are multiple problems with key data as outlined in points below, but the most important issue is the biological activity - if any - of the releasedIL-1β via this proposed exit pathway has not been addressed. The essays used are detecting IL-1β. But is it biologically active? This needs to be properly tested. Otherwise this is if anything a disposal pathway.

3. The interactions between IQGAP1 and GSDMD are shown by co-IPs nut no direct interaction has been established. Appropriate methods should be used to address and establish (rule in or out) that. Importantly, the IQGAP1 could be swept into large protein complexes with ESCRTs engaged in countering plasma membrane permeabilization, a well-established process.

4. Exosome and extracellular vesicles release accompany plasma membrane damage caused by GSDMD or other agents. This is a well known phenomenon, and yet the authors do not account or address any of that.

5. Others have shown that IQGAP1 in cooperation with TSG101 and other ESCRT protesin protects against GSDMD pores at the plasma membrane. It is possible that what the authors here are reporting what is actually a part of the previously published processes at the plasma membrane during GSDMD pore formation and pyroptotic plasmalemma permeabilization. This needs to be fully and formally addressed.

6. Further problems with key data: the 'endosomal localization' (?) in Fig. 4 is based on poor imaging and absence of quantification. (a) The only quantification is with EEA1 (early endosomal marker, not MVBs) in panel A, and not with CD63.

7. Along the same lines, the images in Fig4B with CD63 "show" some overlaps in yellow, but there are so many dots (background fluorescence ?) with IQGAP1 that one wonders if the authors were to flip by 180 degrees yellow and green images (randomizing) would the number of yellow dots remain the same... No quantification/statistics of any kind is a big issue here.

8. TSG101 is an upstream ESCRT in ESCRT machinery which recognizes the cargo protein but doesn't directly regulate the membrane remodeling. Did authors check other ESCRT proteins?

9. In the abstract the authors mention that IQGAP1 bridges GSDMD to ESCRT machinery to promote IL-1β containing exosomes in response to NLRP3 inflammasome activation but haven't shown any data on NLRP3 in this study.

10. Is IL-1β biologically active?

11. If increased in exosomes, how does IL-1β contact the receptors on cells to achieve its biliogical activity?

12. There are some known paradoxical relationship with GTP and GDP states known regarding IQGAPs and Rho GTPases. It seems that the authors may not be aware of those and believe that GTP state (typically associated with "active" small GTPases) is the ON state. This needs to be resolved. Also, IQGAPs are calmodcuin binding protins and Ca2+ may play a role...

Minor:
1. Please check the sizes of graphs in figure 3A
2. Figure 3E and F and figure 4F graphs are represented differently.
3. In figure 6A author haven't pointed out specific structure to look.
4. On any of the western blot author haven't shown the marker to represent the specific size of the proteins.

Referee #2:

Liao et al. (the authors) identify IQGAP1 as a potential interaction partner of gasdermin-D (GSDMD). They show that interaction between IQGAP1 and the C-terminal region of GSDMD is required for secretion of exosomes from YAMC cells upon treatment with LPS+ATP. These exosomes(or the intracellular complexes preceding secretion) contain IQGAP1, GSDMD and among other components TSG101 and activated CDC42. These complexes assemble in CD63 positive endosomes. Ablation of TSG101 expression or inhibition of CDC42 reduced secretion of exosomes upon LPS&ATP treatment. Furthermore, stable assembly of the CDC42, IQGAP1, GSDMD complex requires presence of both IQGAP1 or GSDMD, suggesting a stabilizing function for either proteins. The authors finish their study with the demonstration that exosome secretion is reduced in colonic explants from IQGAP1 or GSDMD KO treated with DSS.

I think the data presented are very interesting and propose a non-canonical role for GSDMD in IL-1 secretion (besides pore formation). The paper is well written, and the experiments are very well controlled. I think most of the conclusion presented are substantiated by the data presented (except detailed below).

My major concern is regarding the upstream events leading to IL-1 secretion in exosomes. We know that stimulation of cells with ATP leads to (despite other events) opening of the pannexin-1 channel by P2X7 stimulation and ion fluxes across the plasma membrane, which are responsible for inflammasome activation. The authors show the presence of cleaved IL-1 beta in the supernatant but cleavage of GSDMD can't be observed (or at least not shown in the study). We don't know if YAMC do activate NLRP3 and if all the processes shown are dependent on inflammasome activation, although the authors claim this in the text. Calcium influx across pannexin-1 channels can be envisioned to lead to activation of CDC42. IQGAP1 is, after all, a calcium responsive protein. Some experiment I would suggest to address questions concerning this knowledge gap in the study:
1. Use MCC950 or 90 mM extracellular KCl to check if secretion of exosomes is dependent on NLRP3 activation
2. Use zVAD-fmk or qVD to check if secretion is dependent on caspase-1 activation
3. Use extracellular EDTA or BAPTA-AM to check if exosome secretion or IQGAP1/GSDMD interaction is dependent on calcium influx

Minor suggestions:
Figure 3: Can the authors show a PLA assay between IQGAP1 ΔIQ and GSDMD as an independent confirmation of abolished interaction?
Figure 4C/D: Quantification of PLA is missing. Is it possible to stain for pro-IL beta to see colocalization with CD63 in wildtype and IQGAP1 KO cells?

PART I: Key Conceptual and Technical Issues Raised by the Reviewers.

Conceptual Issues:

1. The biological activity of the IL-1β released through the exosomal pathway.

Compared to mature IL-1β (~17kDa), pro-IL-1β (~31kDa) retains a propeptide and therefore has a much lower affinity and potency for the IL-1 receptor (IL-1R). Pro-IL-1β is converted to mature IL-1β upon inflammasome activation via proteolytic cleavage by caspase 1 or caspase 8. Since our data showed that the it was the pro-IL-1β that was recognized by the ESCRT system as an exosomal cargo protein, it is natural astute for the Reviewer 1 to question whether the IL-1β released through the exosomal pathway is biologically active (major points **#2** and **#10**).

We actually found that *active caspase 8* as well as other inflammasome components were co-released with polyubiquitinated pro-IL-1β. These data were not included in the original submission for simplicity. Nevertheless, it is important to note that the exosomal release of IL-1β is driven by activated inflammasome capable of processing pro-IL-1β. Consistent with the presence of active caspase 8, we *did detect mature IL-1β* in LPS and ATP-induced exosomes from YAMC cells along with polyubiquitinated pro-IL-1β (**Fig.1A, 3D, 4F,** as indicated by prominent bands around 17kDa**)**. Collectively, the evidence strongly *supports the notion that exosomal IL-1β is biologically active*.

To further validate the bioactivity of exosomal IL-1β, we tested whether exosome preparations can activate the hallmark pathway of IL-1β signaling, NFκB, in a luciferase reporter cell line (**Fig. I** in this document**, and Fig. EV1A and F** in manuscript).

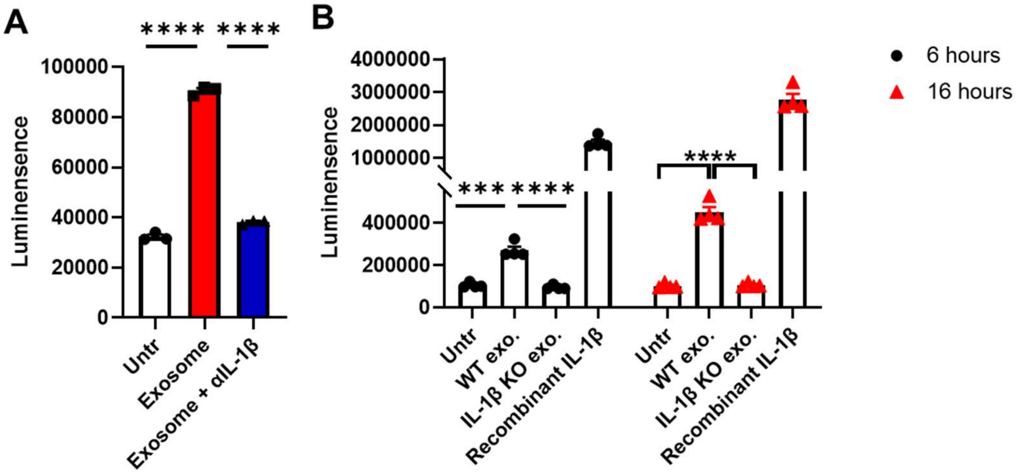

Figure. I A. Exosomal proteins concentrated from 10^7 exosomes were prepared from supernatants of YAMCs that were left untreated or treated with LPS (4hrs) and ATP (30min). HEK293 cells stably expressing IL-1R1 were transfected with an NFκB luciferase reporter construct and stimulated with indicated exosomal proteins in the presence or absence of anti-IL-1β overnight. Cell lysates were analyzed for luciferase activity. **B.** Exosome preparations from colon explants of DSS-treated *wild-type* (WT) and *Il1b-/-* mice were subjected to IL-1β bioactivity assay. Exosomal proteins concentrated from 10^7 exosomes were prepared from supernatants of the colon explants. HEK293 cells stably expressing IL-1R1 were transfected with an NFκB luciferase reporter construct and stimulated with indicated exosome preparations in the presence or absence of anti-IL-1β overnight. Cell lysates were analyzed for luciferase activity. ***, P<0.001 by unpaired two-tailed t test. All experiments were repeated 3 times with consistent results, the representative results are shown.

We enriched for exosomal proteins using Amicon Ultra Centrifugal Filters and utilized a commercial IL-1β activity reporter cell line to measure IL-1β biological activity (Magupalli et al., 2020). An NFκB luciferase reporter (Schindler & Baichwal, 1994) was transfected into a HEK293 cell line that stably overexpresses IL-1R1. Six hours after transfection, concentrated exosome preparations were added to transfected cells. Stimulated cells were lysed and cell lysates were subjected to luciferase activity assay. Exosome preparations from LPS and ATP-treated YAMCs induced about 4-fold increase in luciferase activity, which was abolished in the presence of anti-IL-1β neutralizing antibody (**Fig. IA**). Similarly, exosome preparations from DSS-challenged wild-type but not *Il1b* knockout mouse colon explants also induced over 5-fold increase in luciferase activity (**Fig. IB**).

2. Plasma membrane damage-independent release of exosomes.

As Reviewer 1 referenced in major points **#1** and **#4**, GSDMD-constituted pores in, and the subsequent rupture of plasma membrane was perceived as a major route of exit for IL-1β when GSDMD was first identified as a pyroptosis executor. However, our understanding has rapidly evolved over the last few years, with increasing evidence demonstrating that IL-1β can be released independent of plasma membrane rupture especially in non-myeloid cells such as neutrophils, dendritic cells as well as so-called hyperactivated macrophages (Evavold, Ruan et al., 2018) (Karmakar, Minns et al., 2020) (Zanoni, Tan et al., 2016).

To more conclusively determine whether formation of GSDMD pores contributes to exosomal release of IL-1β in YAMCs, we tested whether GSDMD I105N, a mutant that cannot constitute membrane pores (Kayagaki, Stowe et al., 2015), is capable of mediating the biogenesis of IL-1β-containing exosomes. The GSDMD I105N mutant is a perfect control for the purpose in that we have shown that this mutation does not affect GSDMD-IQGAP1 interaction (**Fig. 2F).** GSDMD I105N showed comparable capacity in mediating the release of poly-ubiquitinated pro-IL-1β (**Fig. IIA** in this document, **Fig. EV1D** in the manuscript), indicating that the pyroptotic activity of GSDMD is dispensable for exosomal IL-1β release. Consistently, the production of polyubiquitinated pro-IL-1β did not associate with an increased in propidium iodide (PI) (**Fig. IIB**, and **Fig. EV1E** in the manuscript), a finding in line with our previous report (Bulek, Zhao et al., 2020).

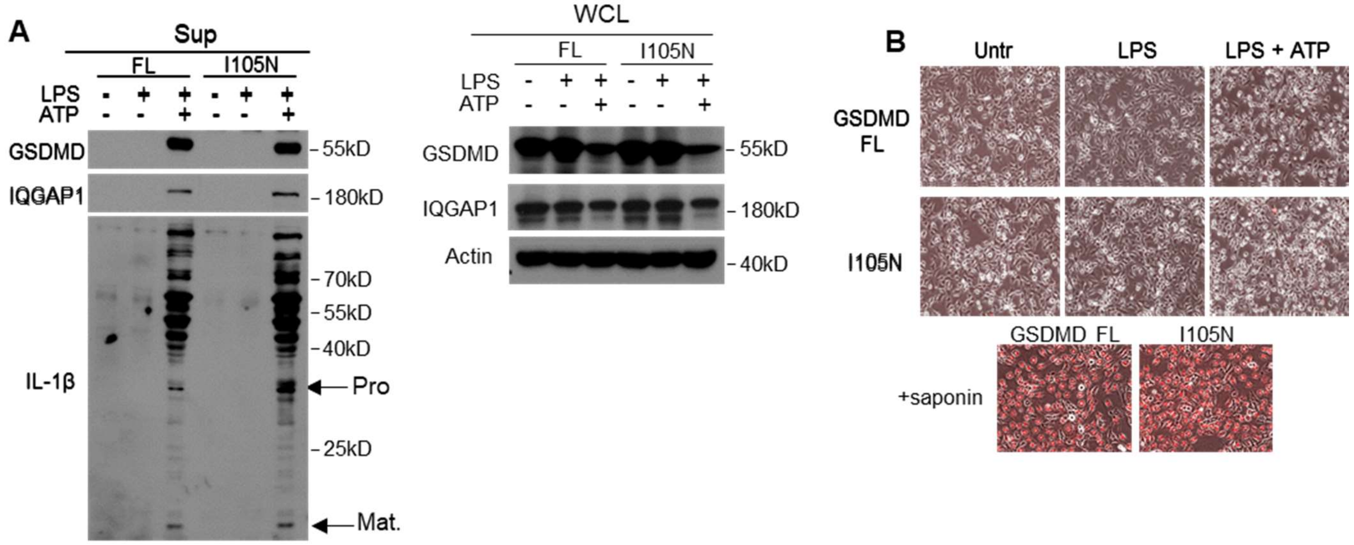

Figure. II A. Western blot analysis of supernatants and whole cell lysates collected from GSDMD KO YAMC cells expressing either wild-type of I105N GSDMD stimulated with or without LPS stimulation for 4 hours or LPS plus ATP (4 hours plus 30 minutes). **B.** GSDMD KO YAMC cells expressing either wild-type of I105N GSDMD were stimulated with or without LPS stimulation for 4 hours or LPS plus ATP (4 hours plus 30 minutes) n phenol red-free DMEM supplemented with 1 µg/ml PI.

3. The dual functions of ESCRT in mediating membrane repair and in exosome biogenesis.

As another point related to membrane rupture, Reviewer 1 referenced the recent discovery that ESCRT-mediated membrane repair, a process also assisted by IQGAP1, is triggered by GSDMD pores. The Reviewer thus questioned whether our observation is merely another rendition of the same cellular process (major point **#5**). We are aware of the referenced studies and have discussed in detail the similarity and distinction of these two pathways in the manuscript. We respectfully disagree with this alternative interpretation suggested by the Reviewer.

If the ESCRT system had been activated to promote membrane repair in response to LPS and ATP stimulation in the YAMC cells, blockade of its activation, by either knocking out IQGAP1 or knocking down Tsg101 as we did in the study, should have led to enhanced cell death and thereby more IL-1β release. *On the contrary, we instead found that inhibition of ESCRT prevented exosomal IL-1β release, thus conclusively ruling out the activation of ESCRT-mediated membrane repair in our experimental system.*

That said, our findings, in light of the prior reports, do beg the question of what determines the role of ESCRT (membrane repair vs. exosome formation). Of note, studies that analyzed the role of ESCRT in membrane repair (Claude-Taupin, Jia et al., 2021, Ruhl, Shkarina et al., 2018) invariably employed stimulations that directly damage the plasma membrane, including treatment with a gentle detergent (Claude-Taupin et al., 2021), overexpressing the N-terminal fragment of GSDMD(Ruhl et al., 2018), or transfection of LPS (Claude-Taupin et al., 2021, Ruhl et al., 2018) which directly activates caspase 11 independent of NLRP3 inflammasome.

Consistent with this prior report, unstimulated IQGAP1-deficient YAMCs appeared more sensitive to plasma membrane damage induced by low-dose saponin, as shown by increased PI uptake and LDH release compared to wild-type cells (**Fig. IIIA** in this document, and **Fig. EV3C**). Notably, the membrane damage induced by low dose saponin did not lead to the release of polyubiquitinated pro-IL-1β in unstimulated YAMCs (**Fig. IIIB** in this document and **Fig. EV3D**). This result underscores the fact that the exosomal pro-IL-1β release, driven by NLRP3-inflammasome activation, is an active process involving cargo protein selection, rather than a passive discharge of cytoplasmic contents. Furthermore, the data suggest that LPS and ATP stimulation is required to engage the ESCRT system for exosome biogenesis. The differences in cell types and stimuli, which dictate how and where the ESCRT system is engaged, likely account for the divergent observations.

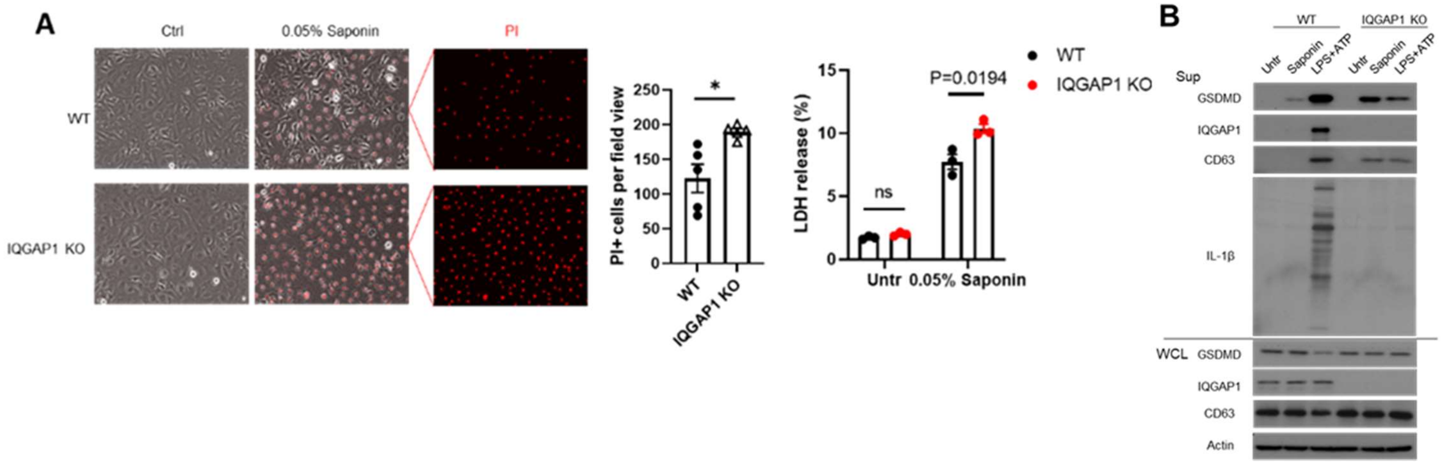

Figure III. A. WT and IQGAP1 KO YAMC cells were left unstimulated and treated with 0.05% saponin for 1min in in phenol red-free DMEM supplemented with 1 µg/ml PI. *, P<0.05 by unpaired two-tailed t test. **B.** Western blot analysis of supernatants and whole cell lysates collected from WT and IQGAP1 KO YAMC cells treated with 0.05% saponin (for 1min) or LPS (4 hours) and ATP (30 min).

4. The involvement of inflammasome in IQGAP1-GSDMD-mediated exosomal IL-1β release.

Both Reviewer 1 (major point **#9**) and Reviewer 2 (major points **#1** and **#2**) astutely noted that additional evidence is needed to substantiate our claim that exosomal IL-1β release is driven by the NLRP3 inflammasome. We agree with the reviewers wholeheartedly on this matter.

MCC950, a small molecule that inhibits NLRP3 oligomerization, we found that inhibition of NLRP3 inflammasome activation abolished the production of poly-ubiquitinated pro-IL-1β and sEVs production in response to LPS and ATP stimulation (**Fig. IVA-B** in this document, **Fig. EV1B**). Furthermore, we found that inhibition of caspase 8 but not caspases 1 attenuated the production of poly-ubiquitinated pro-IL-1β and sEVs (**Fig. IVC-D** in this document and **Fig. EV1C**). Taken together, the data indicate the LPS and ATP stimulation activated NLRP3-casapse 8 inflammasome in the YAMCs.

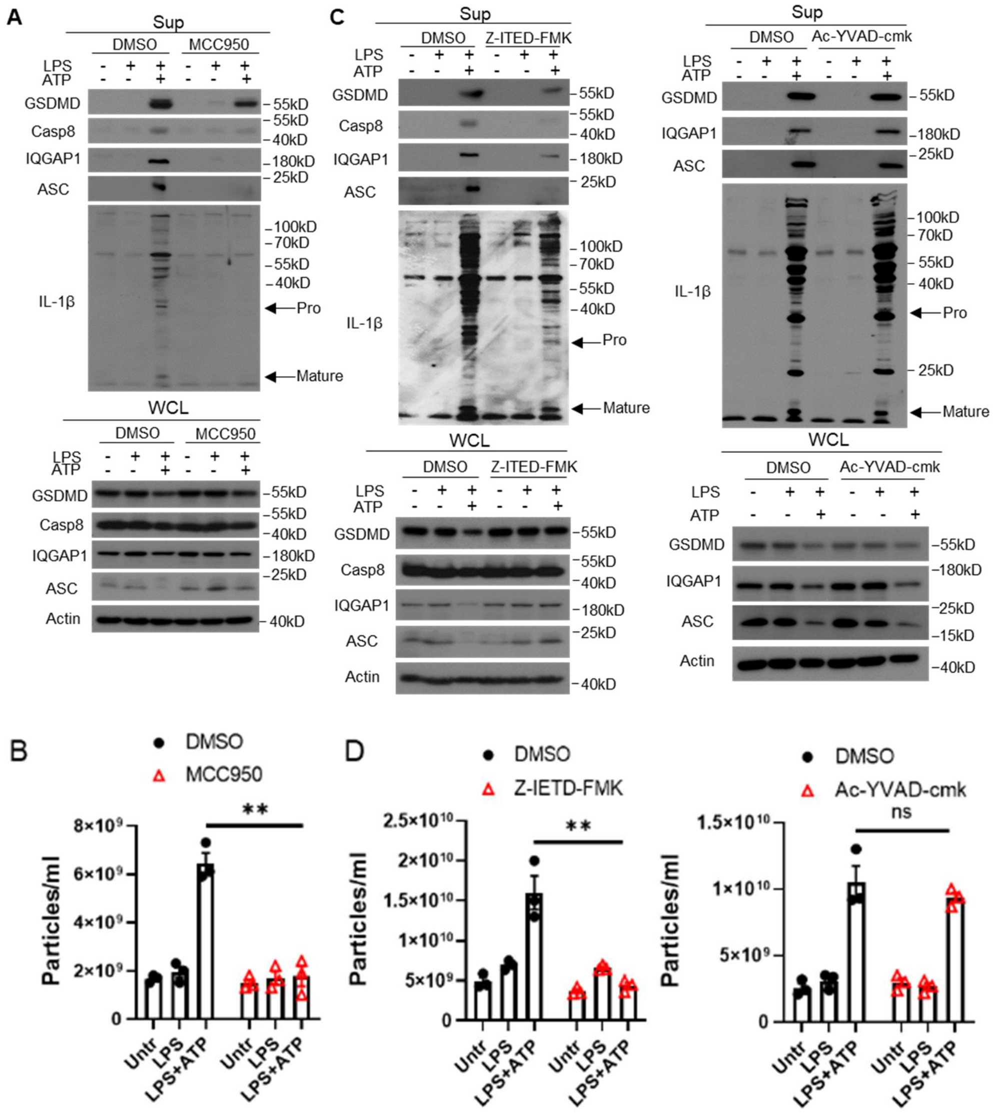

Figure IV. A-B. Western blot (A) and nanoparticle tracking (B) analyses of supernatants and whole cell lysates collected from YAMC cells with and without LPS stimulation for 4 hours or LPS plus ATP (4 hours plus 30 minutes) in the presence or absence of MCC950 (100nM). **C-D.** Western blot (C) and nanoparticle tracking (D) analyses (D) of supernatants and whole cell lysates collected from YAMC cells with and without LPS stimulation for 4 hours or LPS plus ATP (4 hours plus 30 minutes) in the presence or absence of Z-IETD-FMK (10μM) or Ac-YVAD-cmk (10μM).

5. The involvement of calcium signaling in IQGAP1-GSDMD-mediated exosomal IL-1β release.

Both Reviewer 1 (major point **#12**) and Reviewer 2 (major point **#3**) insightfully pointed out the crucial role of calcium signaling in the regulation of IQGAP1. Indeed, calcium flux is induced by ATP stimulation and critically contributes to NLRP3 inflammasome activation. We agree with both reviewers and would like to formally test the involvement of calcium signaling in IQGAP1-GSDMD-mediated exosomal IL-1β release.

Following the reviewer's suggestion, we evaluated the impact of calcium chelation on exosomal release of pro-IL-1β. We found that BAPTA-AM treatment decreased the production of polyubiquitinated pro-IL-1β (**Fig. V** in this document, **Fig. EV3E**). Intracellular calcium mobilization has been long recognized as a trigger for ESCRT activation (Scheffer, Sreetama et al., 2014), which signals through calcium-binding protein-apoptosis-linked gene (ALG). Thus, the data from BAPTA-AM treatment serves to corroborate the involvement of ESCRT system in LPS and ATP-induced exosomal IL-1β release.

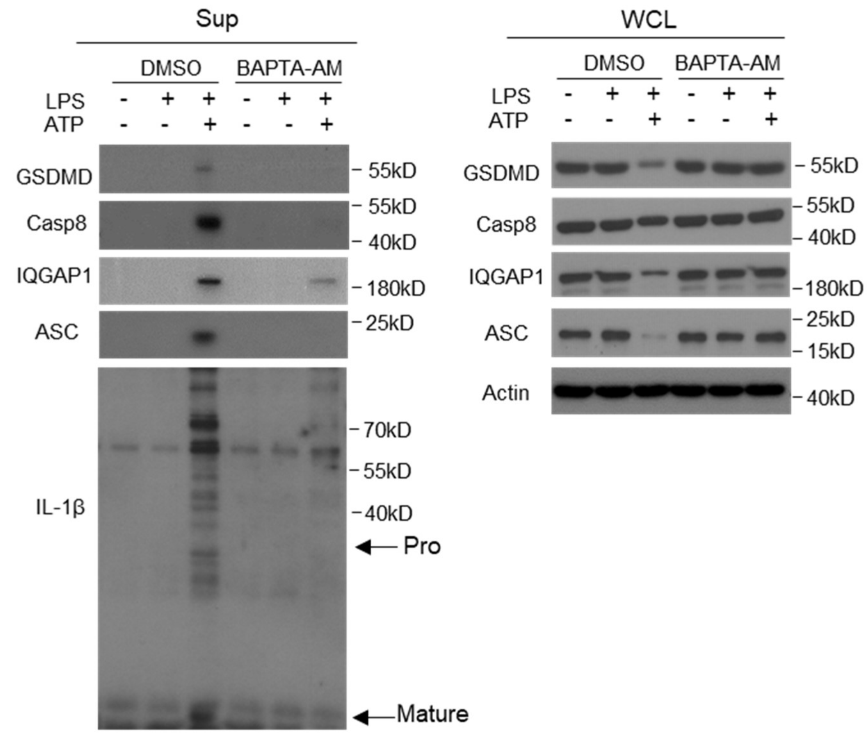

Figure V. Western blot analysis of supernatants and whole cell lysates collected from YAMC cells with and without LPS stimulation for 4 hours or LPS plus ATP (4 hours plus 30 minutes) in the presence or absence of BAPTA-AM **(**6μM**)**.

6. A direct interaction between IQGAP1 and GSDMD.

We identified the IQGAP1-GSDMD interaction using co-immunoprecipitation, which detects not only direct physical interaction but also complexes that are formed via mutual binding to a third component or even lipids. Reviewer 1 requested that we rule out or rule in a direct interaction between IQGAP1 and GSDMD to help discern at which stage IQGAP1 may be involved (major point **# 3**). In addition to co-immunoprecipitation, we showed additional evidence for the IQGAP1-GSDMD interaction using proximity ligation assay (**Fig. EV2**)

We agree with the reviewer that the presented interaction using co-immunoprecipitation data alone does not prove a direct interaction between IQGAP1 and GSDMD. We realized that this is one of the limitations of our study and will include this discussion in the text. Since IQGAP1 is 190kD, it will be technically challenging to test the direct interaction with the recombinant proteins. Since both proteins are required for the same function (IL1-β release), our results collectively support their functional interaction with either direct or indirect physical interaction in a complex.

7. Additional evidence of ESCRT-mediated membrane remodeling in IQGAP1-GSDMD-mediated exosomal IL-1β release.

We showed that Tsg101, which not only recognizes cargo substrate but also recruits downstream components of the ESCRT system, was required for IQGAP1-GSDMD-mediated exosomal IL-1β release. Reviewer contended that Tsg101 is an upstream factor that does not directly participate in membrane remodeling (major point **# 8**). To our knowledge there is very little information on any ESCRT-independent function of Tsg101, thus our data suggest that it is the ESCRT system that mediates the IQGAP1-GSDMD-mediated exosomal IL-1β release. However, we agree with the reviewer that additional evidence would further support its involvement in the process.

Knockdown of Vps24, a component of ESCRT-3 that catalyzes the last step of membrane remodeling during ILV formation, suppressed the release of polyubiquitinated pro-IL-1β and extracellular vesicles (**Fig. VI** in this document**, Fig. EV3A-B**), further demonstrating a critical role of the ESCRT system in this process.

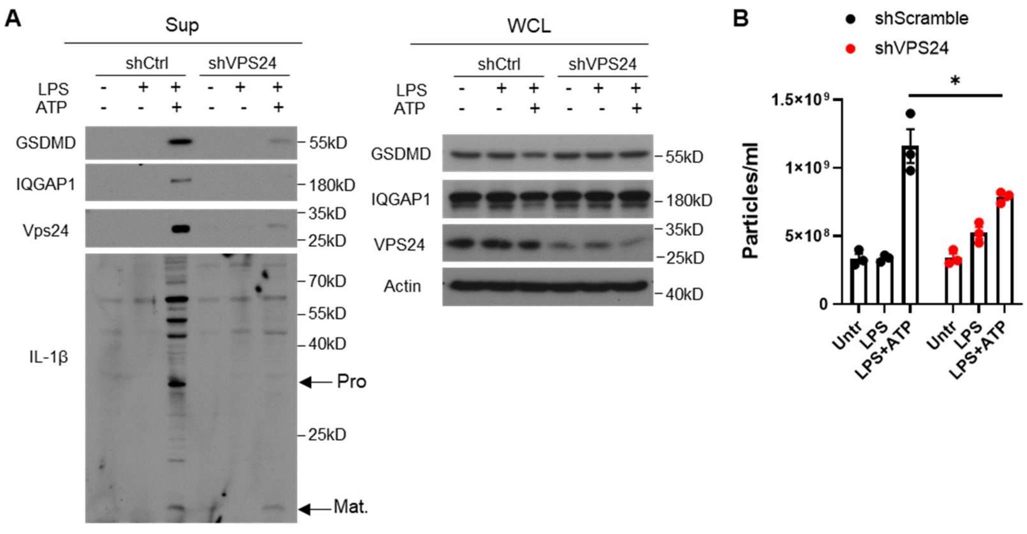

Figure VI. A. YAMCs were transfected with either scramble or Vsp24-targeting siRNA followed by stimulation with LPS for 4 hours 48 hours post transfection. After LPS stimulation, cells were further exposed to ATP for 30 minutes. Supernatants from cells treated described in panel A were subjected to methanol treatment to precipitate total protein, which were then solubilized with Laemmli buffer and analyzed by Western blot. **B.** Nanoparticle tracking analysis of supernatant of scramble siRNA or Vsp24 targeting siRNA transfected YAMC cells treated with LPS plus ATP. *, $P<0.05$ by unpaired two-tailed t test.

Technical Issues:

1. Image quality and quantification.

Reviewer 1 pointed out that the evidence we provided for endosomal localization of IQGAP1 and GSDMD is unsatisfactory, primarily because of inadequate quantification (major points **#6** and **#7**). We thank the reviewer for noting the deficiencies. We have re-analyzed our confocal images and specifically quantified the extent of signal co-localization with CD63. The reviewer also noted that the IQGAP1 signal appears to be excessively abundant and questioned whether the signal was autofluorescence. We have in fact had a control staining using IQGAP1 knockout cells and verified that the detected signals were indeed specific to IQGAP1 (**Fig. VII**)

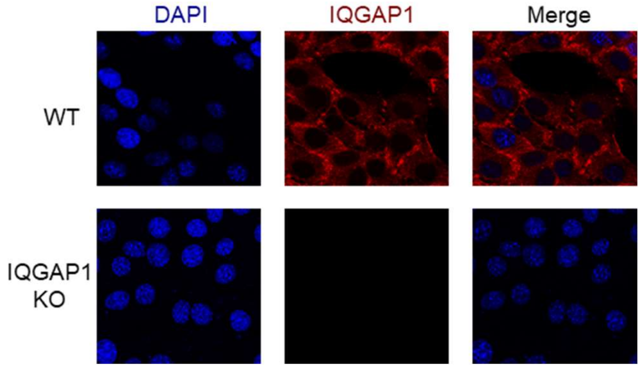

Figure VII. Wild-type and IQGAP1 knockout (KO) YAMC cells were stained for IQGAP1 (red) and imaged by confocal microscopy.

2. Additional PLA and immunofluorescent staining to validate interaction and localization of IL-1β.

Reviewer 2 requested additional proximal ligation assay (PLA) between IQGAP1 ΔIQ and GSDMD as an independent confirmation of abolished interaction. In addition, the reviewer also requested to visualize pro-IL-1β in colocalization with CD63 in wild-type and IQGAP1 KO cells. We performed the prescribed PLA and include the data in revision (**Figs. 3C**). The PLA confirmed the loss of interaction between IQGAP1 ΔIQ and GSDMD. We also performed the suggested staining and found that IQGAP1 deficiency alters pro-IL-1β trafficking to CD63+ puncta (**Fig. 4E**).

PART II: Point-by-point response

Referee #1

In this study Liao et al., report that IQGAP1 bridges GSDMD to the ESCRT system to promote IL-1β release via exosomes. The proposal is that IQGAP1 function as a bridge connecting GSDMD to ESCRT machinery to

promote ubiquitinated-IL-1β and/or pro-IL-1β containing exosomes in response to inflammasome activation. The authors also propose that IQGAP1-GSDMD interaction recruits TSG101 on GSDMD+ late endosome to drive exosomal IL-1β release. In this context CDC42 serves as a molecular switch and enables IQGAP1 to bridge GSDMD to TSG101 during inflammasome-triggered exosome biogenesis.

Some of the proposed relationships seem to be shown in isolation of other mainstream processes of biologically-active v release. The exosome relationships while tantalizing are quite teneous, and it is difficult to assess how significant they are. In the absence of showing that this pathway releases biologically active IL-1β engaged in signaling at target cells (the authors have no assays for activity of IL-1β) the significance of these observations is unclear.

Major:

1. In the introduction, the authors sate that exosomes have been established as exit a mechanism of pyroptosis-associated release of IL-1β referencing a 2007 study. Much has happened since that time and gasdermin pores at the plasma membrane have been exceptionally well defined and accepted as the main pyroptotic mechanism of IL-1β release. The study, if it were to be published, needs to compare the exosomal cargo vs plasma membrane pore release to make sense.

GSDMD-constituted pores in, and the subsequent rupture of plasma membrane was perceived as a major route of exit for IL-1β when GSDMD was first identified as a pyroptosis executor. However, our understanding has rapidly evolved over the last few years, with increasing evidence demonstrating that IL-1β can be released independent of plasma membrane rupture especially in non-myeloid cells such as neutrophils, dendritic cells as well as so-called hyperactivated macrophages (Evavold, Ruan et al., 2018) (Karmakar, Minns et al., 2020) (Zanoni, Tan et al., 2016).

To more conclusively determine whether formation of GSDMD pores contributes to exosomal release of IL-1β in YAMCs, we tested whether GSDMD I105N, a mutant that cannot constitute membrane pores (Kayagaki, Stowe et al., 2015), is capable of mediating the biogenesis of IL-1β-containing exosomes. The GSDMD I105N mutant is a perfect control for the purpose in that we have shown that this mutation does not affect GSDMD-IQGAP1 interaction (**Fig. 2F**). GSDMD I105N showed comparable capacity in mediating the release of poly-ubiquitinated pro-IL-1β (**Fig. EV1D**), indicating that the pyroptotic activity of GSDMD is dispensable for exosomal IL-1β release. Consistently, the production of polyubiquitinated pro-IL-1β did not associate with an increased in propidium iodide (PI) (**Fig. EV1E**), a finding in line with our previous report (Bulek, Zhao et al., 2020).

2. There are multiple problems with key data as outlined in points below, but the most important issue is the biological activity - if any - of the releasedIL-1β via this proposed exit pathway has not been addressed. The essays used are detecting IL-1β. But is it biologically active? This needs to be properly tested. Otherwise this is if anything a disposal pathway.

To further validate the bioactivity of exosomal IL-1β, we tested its ability to activate the hallmark pathway of IL-1β signaling, NFκB, in a luciferase reporter cell line. We prepared exosomal proteins from LPS and ATP-stimulated YAMC cells and colon explants to stimulate an NFκB reporter cell line. This assay provided a specific and robust readout for IL-1β activity, which demonstrated that exosomal proteins contain biologically active IL-1β (**Fig. EV1A and F**).

3. The interactions between IQGAP1 and GSDMD are shown by co-IPs nut no direct interaction has been established. Appropriate methods should be used to address and establish (rule in or out) that. Importantly, the IQGAP1 could be swept into large protein complexes with ESCRTs engaged in countering plasma membrane permeabilization, a well-established process.

We identified the IQGAP1-GSDMD interaction using co-immunoprecipitation, which detects not only direct physical interaction but also complexes that are formed via mutual binding to a third component or even lipids. Reviewer 1 requested that we rule out or rule in a direct interaction between IQGAP1 and GSDMD to help discern

at which stage IQGAP1 may be involved. In addition to co-immunoprecipitation, we showed additional evidence for the IQGAP1-GSDMD interaction using proximity ligation assay (**Fig. EV2A-C**)

We agree with the reviewer that the presented interaction using co-immunoprecipitation data alone does not prove a direct interaction between IQGAP1 and GSDMD. We realized that this is one of the limitations of our study and will include this discussion in the text. Since IQGAP1 is 190kD, it will be technically challenging to test the direct interaction with the recombinant proteins. Since both proteins are required for the same function (IL-1-β release), our results collectively support their functional interaction with either direct or indirect physical interaction in a complex.

Regarding whether IQGAP1 plays a role in countering membrane repair during NLRP3 inflammasome activation in our system, please see our responses to point #4 and 5.

4. Exosome and extracellular vesicles release accompany plasma membrane damage caused by GSDMD or other agents. This is a well-known phenomenon, and yet the authors do not account or address any of that.
5. Others have shown that IQGAP1 in cooperation with TSG101 and other ESCRT protein protects against GSDMD pores at the plasma membrane. It is possible that what the authors here are reporting what is actually a part of the previously published processes at the plasma membrane during GSDMD pore formation and pyroptotic plasma membrane permeabilization. This needs to be fully and formally addressed.

Since the questions #4 and 5 are related, we will address them together in this section.

We are aware of the referenced studies and have discussed in detail about the similarity and distinction of these pathways in the manuscript. For the reasons listed below, we respectfully disagree with the Reviewer about the membrane repairing mechanism as an alternative interpretation for our study.

If the ESCRT system had been activated to promote membrane repair in response to LPS and ATP stimulation in the YAMC cells, blockade of its activation, by either knocking out IQGAP1 or knocking down Tsg101 as we did in the study, should have led to enhanced cell death and thereby more IL-1β release. *On the contrary, we instead found that inhibition of ESCRT prevented exosomal IL-1β release, thus conclusively ruling out the activation of ESCRT-mediated membrane repair in our experimental system.*

That said, our findings, in light of the prior reports, do beg the question of what determines the role of ESCRT (membrane repair vs. exosome formation). Of note, studies that analyzed the role of ESCRT in membrane repair (Claude-Taupin, Jia et al., 2021, Ruhl, Shkarina et al., 2018) invariably employed stimulations that directly damage the plasma membrane, including treatment with a gentle detergent (Claude-Taupin et al., 2021), overexpressing the N-terminal fragment of GSDMD(Ruhl et al., 2018), or transfection of LPS (Claude-Taupin et al., 2021, Ruhl et al., 2018) which directly activates caspase 11 independent of NLRP3 inflammasome.

Consistent with this prior report, unstimulated IQGAP1-deficient YAMCs appeared more sensitive to plasma membrane damage induced by low-dose saponin, as shown by increased PI uptake and LDH release compared to wild-type cells (**Fig. EV3C**). Notably, the membrane damage induced by low dose saponin did not lead to the release of polyubiquitinated pro-IL-1β in unstimulated YAMCs (**Fig. EV3D**). This result underscores the fact that the exosomal pro-IL-1β release, driven by NLRP3-inflammasome activation, is an active process involving cargo protein selection, rather than a passive discharge of cytoplasmic contents. Furthermore, the data suggest that LPS and ATP stimulation is required to engage the ESCRT system for exosome biogenesis. The differences in cell types and stimuli, which dictate how and where the ESCRT system is engaged, likely account for the divergent observations.

6. Further problems with key data: the 'endosomal localization' (?) in Fig. 4 is based on poor imaging and absence of quantification. (a) The only quantification is with EEA1 (early endosomal marker, not MVBs) in panel A, and not with CD63.

We thank the reviewer for noting the deficiency. We re-analyzed the imaging data and quantify the co-localization with CD63 (**Fig. 4B-E**).

7. Along the same lines, the images in Fig4B with CD63 "show" some overlaps in yellow, but there are so many dots (background fluorescence?) with IQGAP1 that one wonders if the authors were to flip by 180 degrees yellow and green images (randomizing) would the number of yellow dots remain the same... No quantification/statistics of any kind is a big issue here.

We have in fact had a control staining using IQGAP1 knockout cells and verified that the detected signals were indeed specific to IQGAP1 (**Fig. VII** in this document).
We thank the reviewer noting the lack of quantification and statistics. We have re-analyzed the image and quantify accordingly (**Fig. 4B-E**).

8. TSG101 is an upstream ESCRT in ESCRT machinery which recognizes the cargo protein but doesn't directly regulate the membrane remodeling. Did authors check other ESCRT proteins?

The membrane remodeling process is primarily mediated by the ESCRT III complex, which consists of multiple essential and a few non-essential subunits. Knockdown of Vps24, a component of ESCRT-3 that catalyzes the last step of membrane remodeling during ILV formation, suppressed the release of polyubiquitinated pro-IL-1β and extracellular vesicles (**Fig. EV3A-B**), further demonstrating a critical role of the ESCRT system in this process.

9. In the abstract the authors mention that IQGAP1 bridges GSDMD to ESCRT machinery to promote IL-1β containing exosomes in response to NLRP3 inflammasome activation but haven't shown any data on NLRP3 in this study.

We agree with the reviewer wholeheartedly on this matter. Using MCC950, a small molecule that inhibits NLRP3 oligomerization, we found that inhibition of NLRP3 inflammasome activation abolished the production of poly-ubiquitinated pro-IL-1β and sEVs production in response to LPS and ATP stimulation (**Fig. EV1B**). Furthermore, we found that inhibition of caspase 8 but not caspases 1 attenuated the production of poly-ubiquitinated pro-IL-1β and sEVs (**Fig. EV1C**). Taken together, the data indicate the LPS and ATP stimulation activated NLRP3-casapse 8 inflammasome in the YAMCs.

10. Is IL-1β biologically active?

Our data suggest that the IL-1β released through the exosomal pathway is active. Please see our response to point #2.

11. If increased in exosomes, how does IL-1β contact the receptors on cells to achieve its biological activity?

The question the reviewer raised touches upon an understudied aspect of exosome biology in general, namely how exosome delivers its cargo to the destination. We respectfully think this aspect is beyond the scope of the current study. We agree that it would be very important to elucidate this mechanism and we do have plans to address it in the future.

12. There are some known paradoxical relationship with GTP and GDP states known regarding IQGAPs and Rho GTPases. It seems that the authors may not be aware of those and believe that GTP state (typically associated with "active" small GTPases) is the ON state. This needs to be resolved. Also, IQGAPs are calmodcuin binding protins and Ca2+ may play a role...

We thank the reviewer for pointing our inaccurate phrasing in our text when we referred to the literature. Indeed, IQGAP1 does not only interact with GTP-bound small GTPases. For instance, IQGAP1 preferentially binds to GDP-bound Rab27a (Kimura, Yamaoka et al., 2013). However, it is important to point out that the formation of GDP-Rab27a-IQGAP1 is also dependent on GTP-CDC42, highlighting the crucial role of GTP-CDC42 in regulating IQGAP1 effector function. This remains consistent with the well-established concept that GTP-CDC42 alters the conformation of IQGAP1, which has been verified by structural analysis of the complex (LeCour, Boyapati et al., 2016).

In response to the reviewer's suggestion, we explored the interaction between IQGAP1-GSDMD and IQGAP1-calmodulin. We found that while GSDMD co-precipitated with IQGAP1, calmodulin was not detected in the same complex **(Fig VIII in this document)**. Based on this, we deduce that calmodulin is not in the same complex with IQGAP1-GSDMD. This likely reflects the fact that both calmodulin and GSDMD binds to the IQ motifs in IQGAP1, forming mutually exclusive complexes. Therefore, we summarize that calmodulin is probably not directly involved in regulating the exosomal release of pro-IL-1β.
On the other hand, using the chelator BAPTA-AM, we did find that calcium signal is required for the production of polyubiquitinated pro-IL-1β (**Fig. EV3E**). Given the established role of calcium signal in activating the ESCRT system, the data provides additional supportive evidence for our proposed mechanism.

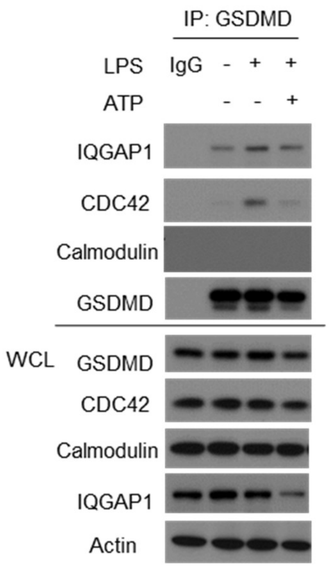

Figure VIII. YAMC cells were treated as indicated and whole cell lysates were subjected to co-immunoprecipitation with anti-GSDMD antibody followed by western blot analysis of indicated proteins.

Minor:
1. Please check the sizes of graphs in figure 3A
We have resized the graphs as requested by the reviewer.

2. Figure 3E and F and figure 4F graphs are represented differently.

We thank the reviewer for noting the style issues and have rectified accordingly.

3. In figure 6A author haven't pointed out specific structure to look.

Arrows now point out the staining pattern in the revised manuscript.

4. On any of the western blot author haven't shown the marker to represent the specific size of the proteins.

We thank the reviewer for noting the minor negligence. Markers are added throughout the figures.

Referee #2

Liao et al. (the authors) identify IQGAP1 as a potential interaction partner of gasdermin-D (GSDMD). They show that interaction between IQGAP1 and the C-terminal region of GSDMD is required for secretion of exosomes from YAMC cells upon treatment with LPS+ATP. These exosomes(or the intracellular complexes preceding secretion) contain IQGAP1, GSDMD and among other components TSG101 and activated CDC42. These complexes assemble in CD63 positive endosomes. Ablation of TSG101 expression or inhibition of CDC42 reduced secretion of exosomes upon LPS&ATP treatment. Furthermore, stable assembly of the CDC42, IQGAP1, GSDMD complex requires presence of both IQGAP1 or GSDMD, suggesting a stabilizing

function for either proteins. The authors finish their study with the demonstration that exosome secretion is reduced in colonic explants from IQGAP1 or GSDMD KO treated with DSS.

I think the data presented are very interesting and propose a non-canonical role for GSDMD in IL-1 secretion (besides pore formation). The paper is well written, and the experiments are very well controlled. I think most of the conclusion presented are substantiated by the data presented (except detailed below).

My major concern is regarding the upstream events leading to IL-1 secretion in exosomes. We know that stimulation of cells with ATP leads to (despite other events) opening of the pannexin-1 channel by P2X7 stimulation and ion fluxes across the plasma membrane, which are responsible for inflammasome activation. The authors show the presence of cleaved IL-1 beta in the supernatant but cleavage of GSDMD can't be observed (or at least not shown in the study). We don't know if YAMC do activate NLRP3 and if all the processes shown are dependent on inflammasome activation, although the authors claim this in the text. Calcium influx across pannexin-1 channels can be envisioned to lead to activation of CDC42. IQGAP1 is, after all, a calcium responsive protein. Some experiment I would suggest to address questions concerning this knowledge gap in the study:

1. Use MCC950 or 90 mM extracellular KCl to check if secretion of exosomes is dependent on NLRP3 activation
2. Use zVAD-fmk or qVD to check if secretion is dependent on caspase-1 activation

In response to points #1 and #2: We agree with the reviewer wholeheartedly on this matter. Because we used classical NLRP3 inflammasome activators as stimuli (LPS plus ATP). We found that MCC950 profoundly blocked the production of poly-ubiquitinated pro-IL-1β and sEVs (**Fig. EV1B**).

Since we only detected active caspase 8 but not active caspase 1 from LPS- and ATP-treated YAMCs (Bulek et al., 2020), we employed more specific caspase inhibitors rather than a pan caspase inhibitor. Consistent with our prior report, we found that inhibition of caspase 8 but not caspases 1 attenuated the production of poly-ubiquitinated pro-IL-1β and sEVs (**Fig. EV1C**). Taken together, the data indicate the LPS and ATP stimulation activated NLRP3-casapse 8 inflammasome in the YAMCs.

3. Use extracellular EDTA or BAPTA-AM to check if exosome secretion or IQGAP1/GSDMD interaction is dependent on calcium influx

Following the reviewer's suggestion, we evaluated the impact of calcium chelation on the formation of IQGAP1-GSDMD complex and exosomal release of pro-IL-1β. We found that BAPTA-AM decreased the production of polyubiquitinated pro-IL-1β (**Fig. EV3E**).

Intracellular calcium mobilization has been long recognized as a trigger for ESCRT activation (Scheffer, Sreetama et al., 2014), which signals through calcium-binding protein-apoptosis-linked gene (ALG). Thus, the data from BAPTA-AM treatment serves to corroborate the involvement of ESCRT system in LPS and ATP-induced exosomal IL-1β release. Additionally, Ca^{2+} surge is also linked to mitochondria dysfunction, which is required for NLRP3 inflammasome activation (Horng, 2014). Therefore, while the data are largely supportive of the involvement of Ca^{2+} in the process, we hesitate to conclude that the impact of BAPTA-AM was due to a direct role of Ca^{2+} on IQGAP1-GSDMD or IQGAP1-CDC42 complex formation. We limit our claim on this data in the manuscript, using it as supportive evidence only.

Minor suggestions:
Figure 3: Can the authors show a PLA assay between IQGAP1 ΔIQ and GSDMD as an independent confirmation of abolished interaction?

We thank the reviewer for this suggestion. We performed the prescribed PLA and include the data in revision (**Fig. 3C**). The PLA confirmed the loss of interaction between IQGAP1 ΔIQ and GSDMD.

Figure 4C/D: Quantification of PLA is missing. Is it possible to stain for pro-IL beta to see colocalization with CD63 in wildtype and IQGAP1 KO cells?

We thank the reviewer for noting the missing quantification. We performed the suggested staining and found that IQGAP1 deficiency alters pro-IL-1β trafficking to CD63+ puncta (**Fig. 4E**).

References:

Bulek K, Zhao J, Liao Y, Rana N, Corridoni D, Antanaviciute A, Chen X, Wang H, Qian W, Miller-Little WA, Swaidani S, Tang F, Willard BB, McCrae K, Kang Z, Dubyak GR, Cominelli F, Simmons A, Pizarro TT, Li X (2020) Epithelial-derived gasdermin D mediates nonlytic IL-1β release during experimental colitis. *The Journal of clinical investigation* 130: 4218-4234

Claude-Taupin A, Jia J, Bhujabal Z, Garfa-Traore M, Kumar S, da Silva GPD, Javed R, Gu Y, Allers L, Peters R, Wang F, da Costa LJ, Pallikkuth S, Lidke KA, Mauthe M, Verlhac P, Uchiyama Y, Salemi M, Phinney B, Tooze SA et al. (2021) ATG9A protects the plasma membrane from programmed and incidental permeabilization. *Nature cell biology* 23: 846-858

Evavold CL, Ruan J, Tan Y, Xia S, Wu H, Kagan JC (2018) The Pore-Forming Protein Gasdermin D Regulates Interleukin-1 Secretion from Living Macrophages. *Immunity* 48: 35-44.e6

Horng T (2014) Calcium signaling and mitochondrial destabilization in the triggering of the NLRP3 inflammasome. *Trends Immunol* 35: 253-61

Karmakar M, Minns M, Greenberg EN, Diaz-Aponte J, Pestonjamasp K, Johnson JL, Rathkey JK, Abbott DW, Wang K, Shao F, Catz SD, Dubyak GR, Pearlman E (2020) N-GSDMD trafficking to neutrophil organelles facilitates IL-1β release independently of plasma membrane pores and pyroptosis. *Nature communications* 11: 2212

Kayagaki N, Stowe IB, Lee BL, O'Rourke K, Anderson K, Warming S, Cuellar T, Haley B, Roose-Girma M, Phung QT, Liu PS, Lill JR, Li H, Wu J, Kummerfeld S, Zhang J, Lee WP, Snipas SJ, Salvesen GS, Morris LX et al. (2015) Caspase-11 cleaves gasdermin D for non-canonical inflammasome signalling. *Nature* 526: 666-671
Kimura T, Yamaoka M, Taniguchi S, Okamoto M, Takei M, Ando T, Iwamatsu A, Watanabe T, Kaibuchi K, Ishizaki T, Niki I (2013) Activated Cdc42-bound IQGAP1 determines the cellular endocytic site. *Molecular and cellular biology* 33: 4834-43

LeCour L, Jr., Boyapati VK, Liu J, Li Z, Sacks DB, Worthylake DK (2016) The Structural Basis for Cdc42-Induced Dimerization of IQGAPs. *Structure (London, England : 1993)* 24: 1499-508

Ruhl S, Shkarina K, Demarco B, Heilig R, Santos JC, Broz P (2018) ESCRT-dependent membrane repair negatively regulates pyroptosis downstream of GSDMD activation. *Science (New York, NY)* 362: 956-960

Scheffer LL, Sreetama SC, Sharma N, Medikayala S, Brown KJ, Defour A, Jaiswal JK (2014) Mechanism of Ca(2)(+)-triggered ESCRT assembly and regulation of cell membrane repair. *Nature communications* 5: 5646
Zanoni I, Tan Y, Gioia MD, Broggi A, Ruan J, Shi J, Donado CA, Shao F, Wu H, Springstead JR, Kagan JC (2016) An endogenous caspase-11 ligand elicits interleukin-1 release from living dendritic cells. 352: 1232-1236

1st Revision - Editorial Decision 16th Aug 2022

Dear Kate,

Thank you for submitting your manuscript to The EMBO Journal. Your study has now been seen by the original two referees and their comments are provided below.

As you can see both referees appreciate that the analysis has been extended and they appreciate the added changes. However, they also feel that some points have not been resolved well enough. I have looked at the comments and I agree with the referees that addressing these last points with experimental data would significantly strengthen the findings. Can we discuss how to best address the last points?

When you submit the revised version will you also please take care of the following editorial points.

- The figures and EV figures need to be uploaded as separate figure files.

- We are missing ORCID ID for Li and Zhao

- Does the LC-MS/MS need to be deposited in a database? See also our guidelines to authors.

- Funding 1S10OD023436-01 is missing in the online submission system.

- We no longer have an author contribution section as we used the information that is provided in the online submission system. You can use the free text box in the system to add more information.

- COI needs to be updated as Disclosure Statement & Competing Interests

- Please check the reference list if more than 10 authors use et al.

- The tables should be re-labelled in the MS text as Table EV1-3

- We don't encourage Data not shown (page 6). Please re-phrase

- We now encourage the publication of source data for electrophoretic blots and quantitative data. See https://www.embopress.org/pb-assets/embo-site/Guide%20for%20SourceData%20Submission-1656066810500.pdf for how to upload and organize it

- We include a synopsis of the paper (see http://emboj.embopress.org/). Please provide me with a general summary statement and 3-5 bullet points that capture the key findings of the paper.

- We also need a summary figure for the synopsis. The size should be 550 wide by [200-400] high (pixels). You can also use something from the figures if that is easier.

- Our publisher has also done their pre-publication check on your manuscript. When you log into the manuscript submission system you will see the file "Data Edited Manuscript file". Please look at the word file and the comments regarding the figure legends and respond to the issues.

Just contact me so that we can set up a time to discuss the needed revisions.

With best wishes

Karin

Karin Dumstrei, PhD
Senior Editor
The EMBO Journal

Use the link below to submit your revision:

https://emboj.msubmit.net/cgi-bin/main.plex

Referee #1:

The authors have done extensive revision in many instances, and should be congratulated on the amount of work. However, on several key issues they chose to argue and maintain their position without further data. Of course, it is up to the editors to decide/adjudicate, but it is the duty of a responsible reviewer to point out such issues - as done below.

This reviewer's opinion remains that, while the manuscript has improved, there are important internal inconsistencies.
Some of the key remaining ones are:
1) If exosomes are the way IL1 gets secreted, as the authors propose, then how come ESCRTs are not important - ESCRTs make instrumental vesicles that generate exosomes.
2) IQGAP1 is not known to function without calmodulin, and yet the authors state that GSDMD binds and displaces calmodulin. Does that mean that GSDM inactivate IQGAP?
3) The issue of "binding" remains unresolved. Many proteins can be found in large complexes, but a claimed (direct) binding needs to be demonstrated and cannot be based on a verbal argument that IQGAP is a large protein (it is not exactly too big).
4) The authors claim that the IL1 they are measuring as released is biologically active, but when asked about how the IL1 if it is trapped inside then exosomes can be biologically active they state that this is beyond the scope of the study. This reviewer's opinion is quite the opposite, that this issue is plumb in the center of the proposed process and should be left right and center within the scope of the study.
5) Given the point 4 above, it is possible if not likely that biologically active IL1 reported in the revision is a leak through pores in membrane (possibly generated by GSDMD, a known poreforming complex) paralleling exosome secretion as an epiphenomenon, and that exosomes are not the source of biologically active IL1.

This reviewer feels that before this work can be published it is important to address these questions, which were posed in the first round of review. This reviewer is willing to re-review the study if the above points can be adequately and whenever possible experimentally addressed. Of course it is up to the editor to decide.

Referee #2:

I thank the authors for addressing my requests raised in the first round of peer review.
I think the data presented is very convincing, however I feel there are some controls missing to fully support the conclusions made by the authors.
1. Biological activity of IL-1: The new data presented in figure EV1F is not well controlled. Yes, the secreted IL-1 has biological activity but we can't judge how much compared to IL-1 secreted by pyroptosis. The way the experiments needs to be performed is that supernatant from stimulated YAMC and BMDMS needs to be collected and the amount of IL-1 needs to be determined by ELISA. If the exosomes of YAMC cells need to be broken up, a low concentration detergent needs to be included in the measurement. Then, equal amounts of supernatant need to be added to the reporter cell line to conclude if the IL-1 secreted via the mechanisms described is bioactive.
2. Calcium: The authors state that they cannot make a definitive conclusion on the role of calcium on NLRP3 mediated shedding of exosomes. If BAPTA-AM really affects NLRP3 activation, as suggested by some publications (referenced by the authors), this should be visible by ASC speck formation as a proxy for NLRP3 activation. I think the authors should check ASC specks in the presence of BAPTA in YAMC cells to determine if calcium is required for assembly and secretion of the GSDMD/IQGAP1/CDC42 complex on exosomes
3. The new data interrogating IL-1 localization is very compelling. One could explain most observations presented with the fact that IL-1 fails to localize to secretory exosomes and that this is the function of IQGAP1 and ESCRT in this context. Do the authors have evidence that exosomes secretion in the absence of IL-1, GSDMD/IQGAP1 complex formation and recruitment to endosomes are unchanged?

We would like to thank the reviewers for their constructive criticism, all the comments and suggestion regarding our manuscript. Please find below our responses in blue font below each point.

Referee #1:

The authors have done extensive revision in many instances, and should be congratulated on the amount of work. However, on several key issues they chose to argue and maintain their position without further data. Of course, it is up to the editors to decide/adjudicate, but it is the duty of a responsible reviewer to point out such issues - as done below. This reviewer's opinion remains that, while the manuscript has improved, there are important internal inconsistencies.

We thank the reviewer for affirming the volume and improvement of our work during revision. We also greatly appreciate the reviewer for the thorough consideration of the data and for raising the remaining concerns. After careful digestion of the reviewer's comment, we feel that some of the remaining issues possibly stem from miscommunication/misunderstanding (we apologize in advance if our data presentation or writing did not clearly convey our message/conclusion). Additionally, we do also hold different opinions with regard to a few conceptual issues from the reviewer. We hope to use this opportunity to advocate for our findings and conclusions.

Some of the key remaining ones are:
1) If exosomes are the way IL1 gets secreted, as the authors propose, then how come ESCRTs are not important - ESCRTs make instrumental vesicles that generate exosomes.

We are claiming that **ESCRT is required** in the process based on data from knocking down of 2 different ESCRT components (Tsg101 and Vps24). To clarify, we are in total agreement with the reviewer that ESCRTs are important for IL-1β release via exosome.

2) IQGAP1 is not known to function without calmodulin, and yet the authors state that GSDMD binds and displaces calmodulin. Does that mean that GSDM inactivate IQGAP?

We agree with the reviewer that calmodulin plays a crucial role as an IQGAP1 modulator. It is **not** our contention to propose a calmodulin-independent function of IQGAP1.

Since we did not detect calmodulin in the same complex with GSDMD, we feel that any connection with calmodulin would likely be an indirect or secondary process that deserves its own dedicated study rather than an add-on to the current manuscript.

Notably, calmodulin was shown to disrupt IQGAP1-CAC42 interaction. Calmodulin clearly changes IQGAP1 conformation and modulates its binding partners in a Ca^{2+} responsive way. But there is no evidence to suggest that IQGAP1-calmodulin complex represents a functionally active complex. https://www.jbc.org/article/S0021-9258(18)37108-4/fulltext In other words, the absence of calmodulin in the GSDMD-IQGAP1 does not mean "inactivation of IQGAP1".

3) The issue of "binding" remains unresolved. Many proteins can be found in large complexes, but a claimed (direct) binding needs to be demonstrated and cannot be based on a verbal argument that IQGAP is a large protein (it is not exactly too big).

Co-immunoprecipitation is a very well-established methodology to detect ligand-induced formation of functional complexes. That is exactly what we have discovered and claimed for IQGAP1-GSDMD interaction. It often takes years and sometimes decades to sort out the direct and indirect interaction

and collaboration with structural biologists to solve the structure of the protein complex. Notably, identification of ligand-dependent functional complexes has led to the discovery of many signaling pathways, including TLR and IL-1 signaling cascades.

With close to 200kDa, the whole protein of IQGAP1 is a huge scaffold that provides binding platform that assembles huge protein complexes. Only fragments of the IQGAP1 structure has been resolved, the shape of the whole protein remains unknown. Importantly, the fragment that is responsible for interaction with GSDMD, the IQ motif, remains the unresolved section of IQGAP1, reflecting the inherent biochemical challenges in protein purification. Without high quality and purity recombinant protein (to which we do not have access), it would be imprudent to conduct biophysical interaction studies.

4) The authors claim that the IL1 they are measuring as released is biologically active, but when asked about how the IL1 if it is trapped inside then exosomes can be biologically active they state that this is beyond the scope of the study. This reviewer's opinion is quite the opposite, that this issue is plumb in the center of the proposed process and should be left right and center within the scope of the study.

5) Given the point 4 above, it is possible if not likely that biologically active IL1 reported in the revision is a leak through pores in membrane (possibly generated by GSDMD, a known poreforming complex) paralleling exosome secretion as an epiphenomenon, and that exosomes are not the source of biologically active IL1.

We strongly disagree with the reviewer's contention that our observation is merely an epiphenomenon and the process is associated with pore formation. In our opinion, we have supplied ample evidence that demonstrates **a pore-forming independent** IL-1β release process.

- We have shown that GSDMD cleavage by caspase 1 (Bulek et al, 2020) and its oligomerization (current manuscript) were both dispensable for GSDMD to mediate IQGAP1-dependent exosome production.
- *Both cleavage by caspase 1 and oligomerization are obligatory biochemical events for pyroptosis.* The fact that exosomal IL-1β release did not require either Caspase 1 cleavage nor GSDMD oligomerization, argues against the involvement of pores in the process.
- Consistently, we did not detect any PI permeability in response to LPS+ATP in YAMCs, further showing that this process does not involve pore formation.
- Lastly, we have also shown (current manuscript) that merely poking holes on plasma membrane is not sufficient to induce IL-1β polyubiquitination, a hallmark of exosome-mediated cargo release.

We also respectively disagree with the reviewer's notion that IL-1β is "trapped" in exosomes. We have provided biochemical, cellular and genetic evidence to indicate IL-1β as a cargo of exosome. Exosomes are emerging as a major conduit for cell-cell communication. Precisely how exosomes cargo is unloaded remains an active area of research. However, growing evidence indicates that the cargos do get unloaded and remain biologically active. Notably, we did provide new data to demonstrate that exosomal IL-1β is biologically active in our revision (Fig. EV1A and EV1F). We do agree with the reviewer that it is important to understand the precise mechanism of cargo unloading allowing IL-1β's recognition of the receptor on the target cells. We have several hypotheses about this, which will be a major undertaken, involving development of multiple new technologies in the lab (beyond the scope of this current study).

This reviewer feels that before this work can be published it is important to address these questions,

which were posed in the first round of review. This reviewer is willing to re-review the study if the above points can be adequately and whenever possible experimentally addressed. Of course it is up to the editor to decide.

Referee #2:

I thank the authors for addressing my requests raised in the first round of peer review.
I think the data presented is very convincing, however I feel there are some controls missing to fully support the conclusions made by the authors.

We thank the reviewer for the positive comment and appreciate the highly constructive comments that helped to advance many key points of our study.

1. Biological activity of IL-1: The new data presented in figure EV1F is not well controlled. Yes, the secreted IL-1 has biological activity but we can't judge how much compared to IL-1 secreted by pyroptosis. The way the experiments needs to be performed is that supernatant from stimulated YAMC and BMDMS needs to be collected and the amount of IL-1 needs to be determined by ELISA. If the exosomes of YAMC cells need to be broken up, a low concentration detergent needs to be included in the measurement. Then, equal amounts of supernatant need to be added to the reporter cell line to conclude if the IL-1 secreted via the mechanisms described is bioactive.

We appreciate the reviewer's suggestion and rationale. However, we have doubts about how such comparison would shed more light on the issue. BMDMs are professional pro-inflammatory cells capable of producing large amount of cytokines and with more robust inflammasome activation. Since ELISA does not discern mature vs pro-IL-1β, even if we normalize assay input based on total IL-1β, we may very well see that IL-1β produced by BMDMs inducing more NFκB activation in the reporter assay. *However, that evidence alone do not negate the fact that exosome preps from YAMC cells also induced IL-1β-dependent NFκB activation.*

2. Calcium: The authors state that they cannot make a definitive conclusion on the role of calcium on NLRP3 mediated shedding of exosomes. If BAPTA-AM really affects NLRP3 activation, as suggested by some publications (referenced by the authors), this should be visible by ASC speck formation as a proxy for NLRP3 activation. I think the authors should check ASC specks in the presence of BAPTA in YAMC cells to determine if calcium is required for assembly and secretion of the GSDMD/IQGAP1/CDC42 complex on exosomes

While we agree with the reviewer that assays evaluating upstream events of inflammasome activation could help to verify whether the process was indeed inhibited by BAPTA-AM, we feel that such information may not provide more substantial insights into the process and advance the current study for a few reasons.

- Firstly, our data show that BAPTA-AM did inhibit exosome production in response to LPS and ATP. It is likely BAPTA-AM treatment inhibited inflammasome activation (whether it's at ASC spec formation or more downstream remains to be investigated). However, such result would not contradict a possible role for Ca^{2+} signaling in regulating IQGAP1-CDC42 activation. Ca^{2+} signaling likely activates both pathways in a coordinated fashion to enable inflammasome assembly and prepare the intracellular vesicle trafficking system for its export.
- Secondly, in an unlikely event that calcium signaling was dispensable for NLRP3 inflammasome in YAMC cells, that observation alone would warrant an independent study.

- Given the profound impact of calcium signaling, especially its well-documented role in inflammasome activation, our preference is not to make broad conclusions based on the evidence.

3. The new data interrogating IL-1 localization is very compelling. One could explain most observations presented with the fact that IL-1 fails to localize to secretory exosomes and that this is the function of IQGAP1 and ESCRT in this context. Do the authors have evidence that exosomes secretion in the absence of IL-1, GSDMD/IQGAP1 complex formation and recruitment to endosomes are unchanged?

In this study we are focusing on inflammasome-induced exosome production. We did not examine in great detail whether baseline exosome production is affected by GSDMD-IQGAP1 deficiency. However, we did not see any difference at baseline within 30 mins. But baseline exosome production in YAMC cells is small and our assays were not designed to capture the difference if there was any.

2nd Revision - Editorial Decision 14th Sep 2022

Dear Kate,

Thank you for submitting your revised manuscript to the EMBO Journal. Your revised manuscript has now been seen by referee #2. The referee appreciates your response but would also like to see some discussion regarding the points raised by referee #1 in the manuscript text - see below.

I think that is a good suggestion and would like to ask to do so in a final revision. I will accept the revised version once it comes back in. When you submit the revision will you also take care of the following pints:

- We are missing ORCID ID for Li

- Please correct the labelling of Tables EV1-3 in the legend as well

- Please upload a "clean" version of the MS when you resubmit

- The source data needs to be organised as one file per figure - see also https://www.embopress.org/pb-assets/embo-site/Guide%20for%20SourceData%20Submission-1656066810500.pdf

- Please also provide a point-by-point response with the revised version.

That should be all! Congratulations on a nice study

With best wishes

Karin

Karin Dumstrei, PhD
Senior Editor
The EMBO Journal

Use the link below to submit your revision:

https://emboj.msubmit.net/cgi-bin/main.plex

Referee #2:

I would appreciate if the authors extend some discussion in their text based on the comments they make in response to both reviewer's comments. Such as:
How could IL-1 exit exosomes, enclosed in a membrane?
What is the relation between function and association of IQGAP1 and Calmodulin?
Is there possible evidence in the literature why IL-1 fails to localize to IQGAP1 (or vesicles in general) in the absence of ESCRT?

Point-by-point response

I would appreciate if the authors extend some discussion in their text based on the comments they make in response to both reviewer's comments.

We have now extended the discussion to incorporate the very illuminative discourse we have had with the reviewers through the revision process. The new text is in blue font.

How could IL-1 exit exosomes, enclosed in a membrane?

Our understanding of exosomal cargo delivery in general is extremely limited, if at all. This remains an ongoing yet underexplored area of research. One tantalizing hypothesis is that extracellular inflammasome activity (Baroja-Mazo et al, 2014; Franklin et al, 2014) along with other enzymes released during inflammatory response, may facilitate the rupture of exosome membrane. Furthermore, it is also plausible that GSDMD will undergo a post-release processing by activated caspase 8 and form pores in exosomes after being released to the extracellular space. Caspase 8 has been shown to exhibit proteolytic activity towards GSDMD at a low efficiency. This may have contributed to a lack of GSDMD processing inside the cells. However, a changing environment, especially an alteration in pH and oxidative stress in the extracellular space as is common in inflamed tissues, may facilitate and expedite GSDMD pore formation, leading to rupture of exosomes.

What is the relation between function and association of IQGAP1 and Calmodulin?

The calcium-responsive protein calmodulin has been shown to inhibit the formation of IQGAP1-CDC42 complex by directly binding to IQGAP1 in response to elevated intracellular Ca^{2+} (Ho et al, 1999; Joyal et al, 1997). We found that Ca^{2+} chelation indeed reduced the production of poly-ubiquitinated pro- IL-1β, implicating a critical role for Ca^{2+} signaling in the process. Intriguingly, calmodulin also binds to the IQ motif in IQGAP1 (Rhoads & Friedberg, 1997), the region required for the formation of IQGAP1-GSDMD complex. Considering that ATP stimulation induces a transient spike followed by oscillation in intracellular Ca^{2+} concentration (Evans & Sanderson, 1999), it is reasonable to speculate that calmodulin may dynamically modulate the formation of GSDMD-IQGAP1-CDC42 complex in tune with Ca^{2+} oscillation. However, whether and how this process indeed occurs during LPS and ATP induced exosome biogenesis requires and warrants further investigation.

Is there possible evidence in the literature why IL-1 fails to localize to IQGAP1 (or vesicles in general) in the absence of ESCRT?

We found that the active GTP-bound form of CDC42 allowed IQGAP1 to bridge GSDMD and the associated inflammasome complex to Tsg101. Tsg101 is part of the ESCRT system that recruits poly-ubiquitinated cargo proteins to late endosome during invagination (Blot et al, 2004; Raiborg & Stenmark, 2002). Consistently, we found that ablation of IQGAP1 prevented IL-1β from relocating to CD63+ vesicles. Taken collectively, our findings reveal a crucial role for IQGAP1 in mediating the activation of ESCRT-mediated cargo loading and membrane remodeling and packaging GSDMD and associated inflammasome into exosome precursor.

Dear Kate,

Thank you for submitting your revised manuscript. I have now looked at everything and all has been sorted out. I am therefore very pleased to accept the manuscript for publication here.

with best wishes

Karin

Karin Dumstrei, PhD
Senior Editor
The EMBO Journal

Please note that it is EMBO Journal policy for the transcript of the editorial process (containing referee reports and your response letter) to be published as an online supplement to each paper. If you do NOT want this, you will need to inform the Editorial Office via email immediately. More information is available here:
https://www.embopress.org/page/journal/14602075/authorguide#transparentprocess

Your manuscript will be processed for publication in the journal by EMBO Press. Manuscripts in the PDF and electronic editions of The EMBO Journal will be copy edited, and you will be provided with page proofs prior to publication. Please note that supplementary information is not included in the proofs.

You will be contacted by Wiley Author Services to complete licensing and payment information. The required 'Page Charges Authorization Form' is available here: https://www.embopress.org/pb-assets/embo-site/tej_apc.pdf - please download and complete the form and return to embopressproduction@wiley.com

EMBO Press participates in many Publish and Read agreements that allow authors to publish Open Access with reduced/no publication charges. Check your eligibility: https://authorservices.wiley.com/author-resources/Journal-Authors/open-access/affiliation-policies-payments/index.html

Should you be planning a Press Release on your article, please get in contact with embojournal@wiley.com as early as possible, in order to coordinate publication and release dates.

If you have any questions, please do not hesitate to call or email the Editorial Office. Thank you for your contribution to The EMBO Journal.

** Click here to be directed to your login page: https://emboj.msubmit.net

EMBO Press Author Checklist

Corresponding Author Name: Xiaoxia Li, Junjie Zhao, Katarzyna Bulek
Journal Submitted to: The EMBO J
Manuscript Number: EMBOJ-2022-110780

USEFUL LINKS FOR COMPLETING THIS FORM
The EMBO Journal - Author Guidelines
EMBO Reports - Author Guidelines
Molecular Systems Biology - Author Guidelines
EMBO Molecular Medicine - Author Guidelines

Reporting Checklist for Life Science Articles (updated January
This checklist is adapted from Materials Design Analysis Reporting (MDAR) Checklist for Authors. MDAR establishes a minimum set of requirements in transparent reporting in the life sciences (see Statement of Task: 10.31222/osf.io/9sm4x). Please follow the journal's guidelines in preparing your
Please note that a copy of this checklist will be published alongside your article.

Abridged guidelines for figures
1. Data
The data shown in figures should satisfy the following conditions:
- the data were obtained and processed according to the field's best practice and are presented to reflect the results of the experiments in an accurate and unbiased manner.
- ideally, figure panels should include only measurements that are directly comparable to each other and obtained with the same assay.
- plots include clearly labeled error bars for independent experiments and sample sizes. Unless justified, error bars should not be shown for technical
- if n<5, the individual data points from each experiment should be plotted. Any statistical test employed should be justified.
- Source Data should be included to report the data underlying figures according to the guidelines set out in the authorship guidelines on Data

2. Captions
Each figure caption should contain the following information, for each panel where they are relevant:
- a specification of the experimental system investigated (eg cell line, species name).
- the assay(s) and method(s) used to carry out the reported observations and measurements.
- an explicit mention of the biological and chemical entity(ies) that are being measured.
- an explicit mention of the biological and chemical entity(ies) that are altered/varied/perturbed in a controlled manner.
- the exact sample size (n) for each experimental group/condition, given as a number, not a range;
- a description of the sample collection allowing the reader to understand whether the samples represent technical or biological replicates (including how many animals, litters, cultures, etc.).
- a statement of how many times the experiment shown was independently replicated in the laboratory.
- definitions of statistical methods and measures:
  - common tests, such as t-test (please specify whether paired vs. unpaired), simple χ2 tests, Wilcoxon and Mann-Whitney tests, can be unambiguously identified by name only, but more complex techniques should be described in the methods section;
  - are tests one-sided or two-sided?
  - are there adjustments for multiple comparisons?
  - exact statistical test results, e.g., P values = x but not P values < x;
  - definition of 'center values' as median or average;
  - definition of error bars as s.d. or s.e.m.

Please complete ALL of the questions below.
Select "Not Applicable" only when the requested information is not relevant for your study.

Materials

Newly Created Materials	Information included in the manuscript?	In which section is the information available? (Reagents and Tools Table, Materials and Methods, Figures, Data Availability Section)
New materials and reagents need to be available; do any restrictions apply?	Not Applicable	

Antibodies	Information included in the manuscript?	In which section is the information available? (Reagents and Tools Table, Materials and Methods, Figures, Data Availability Section)
For **antibodies** provide the following information:<br>- Commercial antibodies: RRID (if possible) or supplier name, catalogue number and or/clone number<br>- Non-commercial: RRID or citation	Yes	Materials and Methods

DNA and RNA sequences	Information included in the manuscript?	In which section is the information available? (Reagents and Tools Table, Materials and Methods, Figures, Data Availability Section)
**Short novel DNA or RNA including primers, probes**: provide the sequences.	Yes	Materials and Methods

Cell materials	Information included in the manuscript?	In which section is the information available? (Reagents and Tools Table, Materials and Methods, Figures, Data Availability Section)
**Cell lines:** Provide species information, strain. Provide accession number in repository **OR** supplier name, catalog number, clone number, and/**OR** RRID.	Yes	Materials and Methods
**Primary cultures:** Provide species, strain, sex of origin, genetic modification status.	Yes	Materials and Methods
Report if the cell lines were recently **authenticated** (e.g., by STR profiling) and tested for mycoplasma contamination.	Not Applicable	

Experimental animals	Information included in the manuscript?	In which section is the information available? (Reagents and Tools Table, Materials and Methods, Figures, Data Availability Section)
**Laboratory animals or Model organisms:** Provide species, strain, sex, age, genetic modification status. Provide accession number in repository **OR** supplier name, catalog number, clone number, **OR** RRID.	Yes	Materials and Methods
**Animal observed in or captured from the field:** Provide species, sex, and age where possible.	Not Applicable	
Please detail **housing and husbandry conditions**.	Yes	Materials and Methods

Plants and microbes	Information included in the manuscript?	In which section is the information available? (Reagents and Tools Table, Materials and Methods, Figures, Data Availability Section)
**Plants:** provide species and strain, ecotype and cultivar where relevant, unique accession number if available, and source (including location for collected wild specimens).	Not Applicable	
**Microbes:** provide species and strain, unique accession number if available, and source.	Not Applicable	

Human research participants	Information included in the manuscript?	In which section is the information available? (Reagents and Tools Table, Materials and Methods, Figures, Data Availability Section)
If collected and within the bounds of privacy constraints report on age, sex and gender or ethnicity for all study participants.	Not Applicable	

Core facilities	Information included in the manuscript?	In which section is the information available? (Reagents and Tools Table, Materials and Methods, Figures, Data Availability Section)
If your work benefited from core facilities, was their service mentioned in the acknowledgments section?	Yes	Materials and Methods

Design

Study protocol	Information included in the manuscript?	In which section is the information available? (Reagents and Tools Table, Materials and Methods, Figures, Data Availability Section)
If study protocol has been **pre-registered, provide DOI in the manuscript**. For clinical trials, provide the trial registration number **OR** cite DOI.	Not Applicable	
Report the **clinical trial registration number** (at ClinicalTrials.gov or equivalent), where applicable.	Not Applicable	

Laboratory protocol	Information included in the manuscript?	In which section is the information available? (Reagents and Tools Table, Materials and Methods, Figures, Data Availability Section)
Provide DOI OR other citation details if **external detailed step-by-step protocols** are available.	Not Applicable	

Experimental study design and statistics	Information included in the manuscript?	In which section is the information available? (Reagents and Tools Table, Materials and Methods, Figures, Data Availability Section)
Include a statement about **sample size** estimate even if no statistical methods were used.	Not Applicable	
Were any steps taken to minimize the effects of subjective bias when allocating animals/samples to treatment (e.g. **randomization procedure**)? If yes, have they been described?	Not Applicable	
Include a statement about **blinding** even if no blinding was done.	Not Applicable	
Describe **inclusion/exclusion criteria** if samples or animals were excluded from the analysis. Were the criteria pre-established? If sample or data points were omitted from analysis, report if this was due to attrition or intentional exclusion and provide justification.	Not Applicable	
For every figure, are **statistical tests** justified as appropriate? Do the data meet the assumptions of the tests (e.g., normal distribution)? Describe any methods used to assess it. Is there an estimate of variation within each group of data? Is the variance similar between the groups that are being statistically compared?	Yes	Figures

Sample definition and in-laboratory replication	Information included in the manuscript?	In which section is the information available? (Reagents and Tools Table, Materials and Methods, Figures, Data Availability Section)
In the figure legends: state number of times the experiment was **replicated** in laboratory.	Yes	Figures
In the figure legends: define whether data describe **technical or biological replicates**.	Yes	Figures

Ethics

Ethics	Information included in the manuscript?	In which section is the information available? (Reagents and Tools Table, Materials and Methods, Figures, Data Availability Section)
Studies involving **human participants**: State details of **authority granting ethics approval** (IRB or equivalent committee(s), provide reference number for approval.	Not Applicable	
Studies involving **human participants**: Include a statement confirming that **informed consent** was obtained from all subjects and that the experiments conformed to the principles set out in the WMA Declaration of Helsinki and the Department of Health and Human Services Belmont Report.	Not Applicable	
Studies involving **human participants:** For publication of **patient photos**, include a statement confirming that consent to publish was obtained.	Not Applicable	
Studies involving experimental **animals**: State details of **authority granting ethics approva**l (IRB or equivalent committee(s), provide reference number for approval. Include a statement of compliance with ethical regulations.	Yes	Materials and Methods
Studies involving **specimen and field samples:** State if relevant **permits** obtained, provide details of authority approving study; if none were required, explain why.	Not Applicable	

Dual Use Research of Concern (DURC)	Information included in the manuscript?	In which section is the information available? (Reagents and Tools Table, Materials and Methods, Figures, Data Availability Section)
Could your study fall under dual use research restrictions? Please check biosecurity documents and list of **select agents and toxins** (CDC): https://www.selectagents.gov/sat/list.htm	Not Applicable	
If you used a select agent, is the security level of the lab appropriate and reported in the manuscript?	Not Applicable	
If a study is subject to dual use research of concern regulations, is the name of the **authority granting approval and reference number** for the regulatory approval provided in the manuscript?	Not Applicable	

Reporting

The MDAR framework recommends adoption of discipline-specific guidelines, established and endorsed through community initiatives. Journals have their own policy about requiring specific guidelines and recommendations to complement MDAR.

Adherence to community standards	Information included in the manuscript?	In which section is the information available? (Reagents and Tools Table, Materials and Methods, Figures, Data Availability Section)
State if relevant guidelines or checklists (e.g., **ICMJE, MIBBI, ARRIVE, PRISMA**) have been followed or provided.	Not Applicable	
For **tumor marker prognostic studies,** we recommend that you follow the **REMARK** reporting guidelines (see link list at top right). See author guidelines, under 'Reporting Guidelines'. Please confirm you have followed these guidelines.	Not Applicable	
For **phase II and III randomized controlled trials**, please refer to the **CONSORT** flow diagram (see link list at top right) and submit the CONSORT checklist (see link list at top right) with your submission. See author guidelines, under 'Reporting Guidelines'. Please confirm you have submitted this list.	Not Applicable	

Data Availability

Data availability	Information included in the manuscript?	In which section is the information available? (Reagents and Tools Table, Materials and Methods, Figures, Data Availability Section)
Have **primary datasets** been deposited according to the journal's guidelines (see 'Data Deposition' section) and the respective accession numbers provided in the Data Availability Section?	Not Applicable	
Were **human clinical and genomic datasets** deposited in a public access-controlled repository in accordance to ethical obligations to the patients and to the applicable consent agreement?	Not Applicable	
Are **computational models** that are central and integral to a study available without restrictions in a machine-readable form? Were the relevant accession numbers or links provided?	Not Applicable	
If publicly available data were reused, provide the respective **data citations in the reference list**.	Not Applicable	
